# Overcoming the “Dark Side” of Technology—A Scoping Review on Preventing and Coping with Work-Related Technostress

**DOI:** 10.3390/ijerph19063625

**Published:** 2022-03-18

**Authors:** Elisabeth Rohwer, Joelle-Cathrin Flöther, Volker Harth, Stefanie Mache

**Affiliations:** Institute for Occupational and Maritime Medicine (ZfAM), University Medical Center Hamburg-Eppendorf (UKE), Seewartenstr. 10, 20459 Hamburg, Germany; joelle-cathrin.floether@studium.uni-hamburg.de (J.-C.F.); harth@uke.de (V.H.); s.mache@uke.de (S.M.)

**Keywords:** digitalisation, information technology, technostress, mental health, occupational health, workplace well-being, mitigation, prevention, health promotion, literature review

## Abstract

In the course of the digitalisation of work, the phenomenon of technostress is increasingly being examined. While there is a plethora of research on its causes and consequences, a growing body of research on mitigating work-related technostress is emerging. In order to identify opportunities to overcome this “dark side” of technology, this scoping review aims to provide a comprehensive overview of the current state of research on how to prevent and cope with work-related technostress. The databases PubMed, MEDLINE, PsycInfo, PSYNDEX, and Web of Science were searched in the time period between 2008 and 2021. The studies were screened independently by two authors and selected based on predefined inclusion and exclusion criteria. Sixty-two studies were included and their methodological quality was assessed using standardised checklists. Resources were identified at the technical, organisational, social and personal level, including, e.g., leadership, organisational and technical support as well as self-efficacy and IT mindfulness. Problem- and emotion-focused coping strategies were, e.g., seeking support or distancing from IT. None of the included studies investigated prevention measures, emphasising a dearth of research that needs to be addressed in the future. Nevertheless, the identified resources and coping strategies provide starting points to address adverse work- and health-related consequences and reduce work-related technostress.

## 1. Introduction

In the working context, information and communication technologies (ICT) have become widely adopted over the past few years and have recently experienced a further boost from the coronavirus disease 2019 (COVID-19) pandemic, which necessitated remote, and thus, digital working arrangements [1,2]. While ICT often entail beneficial qualities and facilitate our work, they may also be stressful or even harmful to our health [3,4]. Although technostress, like stress in general, is a process depending on an individual’s experience and appraisal [5], it has often been referred to as the “dark side” of technology [3,4,6,7]. Since the term was coined by Craig Brod in 1984, technostress is widely understood as the “inability to adapt or cope with new computer technologies in a healthy manner” [8] (p. 16). Based on this definition, Ragu-Nathan and colleagues (2008) elaborated five technostress creators which cause this specific type of stress [9,10]. Firstly, techno-overload refers to the technology-related demand to work longer and faster, whereas constant connectivity and, consequently, a diffusion of work into private life are defined as techno-invasion [10]. Techno-complexity implies an individual’s difficulty to understand certain tasks or conditions [10,11]. Moreover, techno-insecurity can be triggered, e.g., when employees feel threatened with losing their jobs due to their perceived insufficient understanding of technologies or as a consequence of automation. Lastly, techno-uncertainty refers to stressful situations with ambiguous expectations or outcomes [10]. Ragu-Nathan et al. thus conclude that technostress extends other stress-related theoretical frameworks [10]. In support of this, a more recent scientometric analysis has found that most of the examined studies on work-related technostress were based on the transactional stress model by Lazarus [4,12,13]. In this vein, technostress is associated with health- and work-related outcomes, such as exhaustion [12], satisfaction and performance [13].

### 1.1. Theoretical Framework

**Figure 1:**
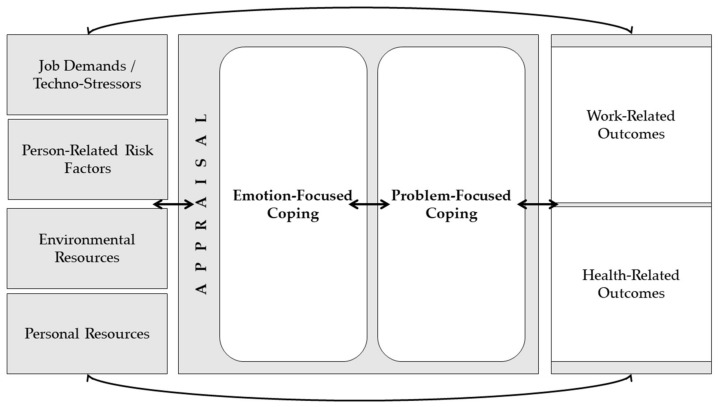
Occupational psychological stress model (adapted based on Bamberg et al. [17]).

As Tarafdar et al. [5] have described and Bondanini et al. [4] have recently shown, the technostress literature is predominantly based on Lazarus’ approach to the transactional stress model [14,15]. Although this theoretical framework seems appropriate for the technostress concept, its insufficient consideration of stress-inducing conditions has been criticised in the literature [16,17]. The occupational psychological stress model (German: Arbeitspsychologisches Stressmodell) by Bamberg and colleagues [16,17] offers a suitable extension. This model adapts key elements of the stress-and-strain concept by Rohmert and Rutenfranz [18] and the transactional stress model by Lazarus and Folkman [14]. It also combines the transactional approach including appraisal and coping strategies of Lazarus and Folkman [14] with the key elements job demands, job and personal resources of the job demands–resources model of Bakker and Demerouti [19,20], which has recently been used as a theoretical framework in the technostress literature as well e.g., [6,21,22,23,24]. Therefore, the occupational psychological stress model considers job demands or stressors, person-related risk factors, environmental and personal resources as well as primary and secondary appraisal, and problem- or emotion-focused coping. The consequences of stress are divided into short- and long-term consequences on somatic, cognitive–emotional, and behavioural levels [16,17]. These consequences can affect the individual and social environments and organisations, potentially triggering a spiral of stress [17], comparable to the job demands–resources model’s gain and loss spirals proposed by Bakker and Demerouti [19,20]. Due to the more comprehensive consideration of external and internal factors and their interaction, the scoping review was based on this occupational psychological stress model [16,17], as depicted in Figure 1.

### 1.2. Study Aim

Recently, the COVID-19 pandemic boosted working from home and, thus, private and work-related ICT use [2]. In this light, counteracting technostress becomes even more vital considering mental health issues [4]. However, remote work environments during the COVID-19 pandemic represent different circumstances that need to be distinguished from digital work outside the pandemic context [25]. Recent growing research on work-related technostress outside this specific context has already elaborated the causes and effects of technostress on an extensive scale [4,9,26,27], mostly focusing on the so-called “dark side”, the negative aspects, or techno-distress as characterised by Tarafdar and colleagues [5]. Previous literature reviews in this context have focused, e.g., on remote e-work and well-being [26], the psychological impacts of new ways of working [27] or the effects of technological developments on work [28]. With regard to technostress, previous literature reviews have addressed associated symptoms and risks [29], causes, strains, inhibitors and impacts [30], or provided a more general overview of technostress in organisations [31] as well as its relation to mental health and work outcomes [32,33,34]. Yet, far less research has investigated how to address these causes or deal with adverse effects resulting from technostress [5,9]. Specifically, there is considerably less scientifically substantiated evidence on adequate coping strategies [5,22,27,35] or prevention measures [36,37]. Apart from more specific reviews which could not identify strategies to prevent technostress among nurses [37] or focused on coping with discrepant information technology events [35], there is no comprehensive systematic overview of research on how to prevent and cope with technostress yet. Due to the previous research focus on causes and consequences technostress and fewer studies on coping and prevention, the scoping review method was chosen to explore and include a broader extent of the current literature and to filter out relevant results. Thus, the diversity of available research from heterogeneous sources and methodological approaches could be addressed [38]. Thereby, this scoping review aims at gathering existing empirical findings and at providing an overview of the current literature. For this purpose, the findings will be mapped to explore and systematically summarise the current state of research outside the pandemic context of COVID-19 [38,39]. Considering the topicality of the COVID-19 pandemic and related changes in remote work, the review results will be discussed by taking initial pandemic-related studies into account.

## 2. Materials and Methods

As stated in the first section, a scoping review was conducted to examine the extent and nature of the current state of research as well as to summarise findings and identifying research gaps in the existing literature on preventing and coping with work-related technostress [40,41]. For this purpose, this scoping review followed the methodological framework suggested by Arksey and O’Malley [40], its extension by Levac et al. [42] and the recent recommendations by Peters et al. [43].

### 2.1. Identifying the Research Questions

Based on the theoretical background described above, this scoping review addresses the following research questions:

1. What kind of techno-stressors, job demands, and person-related risk factors have already been identified?
2. Which environmental and personal resources (including coping strategies) help employees and managers to cope with work-related technostress?
3. Which behavioural and structural prevention measures have already been examined and have proven to be effective in counteracting adverse effects of work-related technostress on employees and managers?
4. How do these different resources, coping strategies and prevention measures mitigate adverse health- and work-related effects of technostress among employees and managers?

### 2.2. Identifying Relevant Studies

A search string with various search terms for the research questions was iteratively formed and tested. Considering the interdisciplinarity of the technostress concept [5,22,44] as well as the research questions’ focus on occupational (mental) health, coping and prevention, both medical and psychological as well as interdisciplinary databases were selected. The initial search string was adapted individually for each database. Relevant studies were identified by searching the following five electronic databases in November 2020 and August 2021: PubMed, MEDLINE, PsycInfo, PSYNDEX and Web of Science. In addition, further eligible studies were identified through a manual search. All five search strings are provided as Supplementary Materials (Tables S1–S5).

### 2.3. Study Selection

Predefined eligibility criteria based on the extended scheme considering participants, concept and context (PCC scheme) were used to decide on the inclusion and exclusion of studies [43]. To be included, the studies had to contain at least one variable that could be assigned to one of the five techno-stressors from the technostress concept of Ragu-Nathan et al. [10] or examine them in a qualitative approach. Furthermore, the study had to address work-related technostress among employees or managers. Studies among self-employed workers, with non-work-related and student samples, or examining technostress in the private context were excluded. Additionally, studies had to include either an intervention or prevention measure, coping strategy or an environmental or personal resource to mitigate technostress. Moreover, the outcomes examined in the studies had to be health- or work-related, i.e., measures of physical and mental health and well-being (e.g., stress, exhaustion, burnout, work–life balance) or, e.g., satisfaction, commitment and engagement, productivity and performance at work (see Supplementary Materials Table S6). Studies conducted during the COVID-19 pandemic were excluded from the analysis unless they explicitly described that the participants’ work remained unaffected by the pandemic. An exclusive examination of personality traits in dealing with technostress also led to exclusion. From a methodological perspective, empirical field studies following a qualitative, quantitative or mixed-methods approach published in scientific journals, conference papers, research reports, theses or dissertations were included. Non-empirical studies, such as conceptual papers, commentaries, editorials, or opinions, as well as reviews, meta-analyses and experimental studies in laboratory settings were excluded. For two studies [45,46], the authors were contacted and partly provided additional information on the methodology of their study [45]. Considering the authors’ language skills, studies had to be published in English or German and were excluded otherwise due to limitations of further resources. An initial search in the databases and a manual search were performed on 26 November 2020. To represent the most current research possible, an update was carried out on 20 August 2021 and the same search string was re-run in all of the five databases. Additionally, further sources were identified in a manual search on the same date. Table 1 provides an overview of the eligibility criteria.

The study selection was carried out in two steps: first, one author (E.R.) screened the titles and abstracts of all identified studies for eligibility criteria, then two authors (E.R. and J.-C.F.) independently screened the full texts of the remaining studies for eligibility criteria. The inter-rater reliability was calculated using Cohen’s kappa. Disagreements in screening were discussed among the authors until a consensus was reached.

### 2.4. Charting the Data

The charting of the data was based on Arksey and O’Malley’s [40] guidelines for scoping reviews. Accordingly, Table S6 provided in the Supplementary Materials includes the following information: authors(s), year of publication, study location(s), methodology (i.e., publication type, methodological approach, study design(s)), study population and sample size, aim(s) of the study, outcome measures (i.e., main measurements), and important results. Furthermore, the included studies were coded and categorised in a deductive approach based on the theoretical framework (job demands, person-related risk factors, environmental resources, personal resources, coping strategies, health-related outcomes and work-related outcomes) [16,17] with MAXQDA software (version 12, VERBI Software) [47].

### 2.5. Collating, Summarising, and Reporting the Results

According to Levac et al.’s [42] recommendation and addition to Arksey and O’Malley [40], qualitative data analytical techniques were used to conduct the thematic analysis of the data. To link the results of this scoping review to its aim, purpose and research questions, the results are reported based on the structure of the theoretical framework presented in Section 2.1. The discussion part provides broader implications stemming from the results for further research and practice, as suggested by Levac et al. [42].

### 2.6. Quality Assessment

While methodological quality assessment was challenged by Arksey and O’Malley [40] as well as Levac et al. [42], more recent literature recommends conducting a methodological quality assessment in scoping reviews [41]. As it contributes to the aim of this scoping review to map the literature and identifying gaps in the current state of research, a quality assessment was conducted to evaluate the methodological quality of the current state of research on preventing and coping with work-related technostress. For this purpose, two of the authors (E.R. and J.-C.F.) used the checklists provided by the Joanna Briggs Institute’s critical appraisal tool for analytical cross-sectional studies [48], cohort studies [49] and qualitative research [50]. To assess the methodological quality of mixed-methods studies, a combination of the respective checklists [48,50] was applied for the qualitative and quantitative components of the study.

## 3. Results

**Figure 2:**
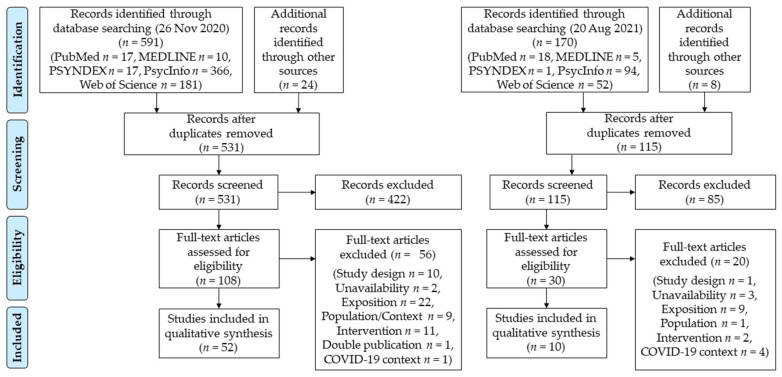
PRSIMA flow diagram depicting the study selection process.

Searching the databases resulted in an initial total of 591 identified records. A further 25 records were identified through a manual search and based on the references of the included studies. Of these 616 identified records, 531 records were screened based on their titles and abstracts by one author (E.R.) after duplicates were removed. A total of 108 studies were identified as eligible for full-text screening, which was conducted by two authors (E.R. and J.-C.F.). Finally, 52 studies were identified and included. According to Landis and Koch [51], the inter-rater reliability among the authors was substantial based on Cohen’s kappa (κ = 0.65). During the update in August 2021, 115 further records were identified, of which 85 were excluded after screening titles and abstracts (E.R.). The remaining 30 records were included in the same full-text screening procedure (E.R. and J.-C.F.), resulting in an inclusion of ten additional records for the qualitative synthesis of this review. Cohen’s kappa of κ = 0.65 indicates a substantial inter-rater reliability for the update process as well [51]. All deviations were discussed individually between the authors until agreement was reached. As a result, a total of 62 studies were included in the qualitative synthesis of this scoping review. A visualisation of the study selection process based on the Preferred Reporting Items for Systematic Reviews and Meta-Analyses (PRISMA) [52] is provided in Figure 2.

### 3.1. Study Characteristics

The 62 included studies were published between 2008 and 2021, with the majority of studies published in 2020 (*n* = 18) and 2019 (*n* = 9). The studies were distributed internationally across 20 different countries and five continents. The majority of the studies were conducted in or published by authors from the United States of America (*n* = 17). At continental level, most of the studies were conducted in Europe (*n* = 24). Table 2 provides further details regarding the international distribution of the included studies.

Among the included studies, there was a large majority of quantitative cross-sectional studies (*n* = 48) [6,9,10,13,21,22,24,46,53,54,55,56,57,58,59,60,61,62,63,64,65,66,67,68,69,70,71,72,73,74,75,76,77,78,79,80,81,82,83,84,85,86,87,88,89,90,91,92] as can be seen in Table S6 in the Supplementary Materials. In total, 53 studies followed a quantitative methodological approach [6,9,10,13,21,22,23,24,46,53,54,55,56,57,58,59,60,61,62,63,64,65,66,67,68,69,70,71,72,73,74,75,76,77,78,79,80,81,82,83,84,85,86,87,88,89,90,91,92,93,94,95,96], of which 5 studies in a longitudinal design were included [23,93,94,95,96]. Eight studies were of qualitative nature [45,97,98,99,100,101,102,103] and one mixed-methods study [7] was included. One paper included a cross-sectional and a longitudinal study [23]. There were no intervention studies among the included studies.

The included studies examined a total of 40,940 participants. Gender information was provided for 36,949 participants, of whom 17,996 were female and 18,894 were male. For 59 participants, it was explicitly stated that they did not give any information about their gender. It becomes apparent that many studies did not provide (complete) information on the participants’ gender, while in seven studies [6,7,48,79,94,100,102], no demographic information was provided regarding gender. In the 52 studies reporting the age of participants, it ranged from 17 to 75 years, with a mean of 40.62 years [6,7,9,10,21,22,23,24,46,53,54,55,56,57,58,59,60,61,62,63,64,65,66,67,68,69,70,71,72,73,74,77,79,80,81,82,83,84,85,86,87,88,89,90,91,92,93,94,95,96,97,101]. Ten studies did not provide any information regarding the age of participants [13,45,75,76,78,98,99,100,102,103].

Many studies lacked more detailed information on the occupational setting, population or investigated sample. However, all included studies were conducted with employees from different industries who use or rely on ICT in their daily work. Thus, most of the included samples were simply categorised as “employees using ICT at work” (*n* = 30) [7,10,23,46,58,59,60,61,63,65,66,67,68,71,73,74,77,79,81,82,84,85,88,90,92,93,94,95,96,101]. Other larger groups were employees at educational and research institutions (*n* = 6) [48,58,59,73,76,91], salespeople or sales professionals (*n* = 6) [6,9,24,56,83,87], knowledge workers (*n* = 5) [7,22,66,97,98], and public sector employees (*n* = 3) [15,55,64]. The mean work experience among those studies reporting it (*n* = 28) was 9.15 years [6,9,13,21,24,54,56,57,59,60,65,66,67,68,71,72,75,80,83,84,86,87,89,91,93,95,97,103]. More study characteristics are provided in the Supplementary Materials in Table S6.

### 3.2. Quality Assessment

After assessing the quality of the included studies independently, the inter-rater reliability based on Cohen’s kappa (κ = 0.53 for the initial search, κ = 0.45 for the update) indicated a moderate agreement according to Landis and Koch [51] between the two authors (E.R. and J.-C.F.). All of the included qualitative studies fulfilled at least 50% (5 out of 10) of the checklist’s quality criteria. Regarding the included longitudinal studies (*n* = 5), they all fulfilled at least six out of eleven criteria (55%). Among the included cross-sectional quantitative studies, ten (20%) fulfilled all of the eight criteria. Forty-five cross-sectional studies (92%) fulfilled at least half of the quality criteria. Following the study aim to provide a holistic overview of the existing literature, no studies were excluded based on their quality assessment. Rather, their methodological quality will be reflected upon in the discussion section. The results of the methodological quality assessment of the included studies are provided in detail in the Supplementary Materials in Tables S7–S9.

### 3.3. Job Demands and Person-Related Risk Factors

With regard to the first research question, what kind of job demands and techno-stressors as well as person-related risk factors have already been described in terms of work-related technostress in the literature, job demands were identified at the organisational and technical level, including the five technostress creators by Ragu-Nathan et al. [10].

#### 3.3.1. Job Demands: Organisational Level

Twelve studies examined organisational-level demands [48,55,58,63,66,75,86,88,94,97,98,103]. These included response pressure [58,94,103], a competitive climate [53], power centralisation in an organisation [88] and an organisational climate or culture of innovation [63,66,88]. However, the latter was also assumed to be a resource in one study [74]. Task interdependence [86] and task complexity [74], need for redistribution of work and administrative support [103], social conflicts [48,66,103], poor communication [94,103], necessity of emails and confusion of responsibilities [98] were mentioned as well. Lack of support [45] and of sense of achievement [64] were further described. In addition, Andreou found that negative opinions of colleagues concerning technology shaped how new workers thought about it as negative vicarious experiences [97].

#### 3.3.2. Job Demands: Technical Level

All of the included studies analysed technostress based on the definition or including technostress creators based on Ragu-Nathan et al. [10]. One study analysed technostress, personal resources and work-related outcomes with an adaption of the original scale [10], but lacked more detailed information on the included techno-stressors [46]. The authors did not provide further information upon request either. Besides this study, 52 (83.9%) of all included studies examined techno-overload, 43 (69.4%) techno-invasion, 42 (67.7%) techno-complexity, 34 (54.8%) techno-insecurity and 35 (56.5%) techno-uncertainty. Given that qualitative studies usually do not examine relationships between variables, overlapping codings of the included quantitative studies were cross-referenced to illustrate how often relationships between techno-stressors and outcomes were analysed.

Table 3 provides an overview of how many of the included quantitative studies examined the individual technostress creators in total and with regard to work- and health-related outcomes. It demonstrates that techno-overload, techno-invasion and techno-complexity were also most frequently analysed among the quantitative studies, whereas work- and health-related outcomes were examined almost equally often. The most frequently investigated work-related outcomes with regard to techno-stressors included different measures of satisfaction [10,15,21,56,63,64,66,72,73,80,83] and performance [6,15,26,72,73,76,87,96]. The most commonly examined health-related consequences of technostress creators were various forms of exhaustion [9,22,54,55,56,64] as well as strain [62,80,82,86].

Further technology-related stressors which did not refer to the techno-stressors as defined by Ragu-Nathan et al. [10] were mentioned in 20 studies [21,22,45,54,60,61,64,68,70,76,79,85,86,94,97,98,99,100,102,103]. Some of them are technology-induced but may be reinforced by organisational conditions, for example role stress [70], i.e., role ambiguity [21,86], which can be amplified by a high intensity of telework [86], and role overload [21,66,71,80], but also invasion of privacy [66,86,99]. Similarly, IT presenteeism, i.e., being reachable and able to access others [104], was examined in connection with invasion of privacy and especially affected employees with a low intensity of teleworking [86]. Occasionally, specific demands were stated such as terminology misfit between ICT systems and healthcare [103], challenging nature of data [102] and lack of control over dealing with emails [98]. At the same time, a fear of missing out important information was mentioned in qualitative studies [98,103]. Moreover, Kwanya et al. referred to technolust, the continuous desire for brand new technology regardless of whether it is needed and which respondents associated, inter alia, with pressure, frustration and dissatisfaction [45]. Among salespeople, the use of social media for sales activity was examined as an antecedent of technostress [54]. Other technology-related demands included, e.g., performance monitoring [60,64], unreliability of technology [22,48,63,66,103] due to different kinds difficulties [48,62,94,97,103] such as interruptions [22,66,100,103] or not being provided with adequate technology [48,66,69,103] or being dependent on technology [69,77,85]. However, one study could not provide support for significant associations of perceived reliability and technology dependence with technostress [76].

Several studies indicated that relationships between technostress creators and resources as well as work-related outcomes may not be linear, but rather inverted U-shaped [6,79,96]. Accordingly, a moderate level of technostress can contribute to improved performance, whereas low or high levels of technostress degrade performance [96]. In a similar vein, a curvilinear relationship was found between job design and technostress [78] as well as between system feature overload and salespeople’s effort to use technology, administrative performance and outcome performance, respectively. This indicates that after an initial decrease in effort and performance when confronted with technology, salespersons’ effort and performance increase when they are able to process and handle incoming information [6].

#### 3.3.3. Person-Related Risk Factors

Person-related risk factors refer to an individual’s characteristics that may trigger stress, but do not necessarily lead to stress for every individual. Thus, individual differences are taken into account [17]. Twelve of the included studies identified several person-related risk factors that can potentially increase the risk of experiencing technostress [10,54,62,63,64,70,75,77,95,96,97,100]. Among them were many sociodemographic factors such as gender [10,56,76] and age [10,64,95,96,100]. For both of them, the included studies showed contradictory results: some found (partly) significant gender differences [10,54] while others did not [75]. Most of the studies examining age found significant differences in the perception of technostress [10,64,96,100], except for one [95]. Findings indicated that advanced age was associated with increased perception of technostress [64,96,100]. However, one study found the opposite effect of decreasing technostress with increasing age [10]. Additionally, Maier et al. found that neuroticism was related to higher technostress perception while other personality traits revealed no significant effect on technostress [96]. Gaudioso et al. and Hauk et al. examined gender differences in terms of coping strategies. Older employees seemed to engage in coping, but more effectively through the use functional rather than dysfunctional strategies compared to younger ones [63,95]. Examining prevention focus as a regulatory focus did not show that it would amplify the adverse effects of technostress creators [70]. A higher educational level [10] and working full-time rather than part-time [96] was associated with lower technostress perception, whereas literacy facilitation was more strongly associated with techno-overload and techno-complexity among participants with longer work experience [75]. Moreover, low self-efficacy, negative or too positive states of arousal, individual experiences [97], attitudes and beliefs [84,97] and intensity of ICT use in the sense of the number and frequency of use [64,77] determine and may increase technostress perception. Especially when employees used many different technologies, but only rarely, they reported higher levels of technostress [64].

### 3.4. Environmental and Personal Resources

Following the theoretical framework, environmental and personal resources helping employees and managers to cope with work-related technostress were examined subsequently to answer the second and fourth research question. Many of the included studies examined a wide range of resources. Environmental resources were thus assigned to different levels, i.e., the technical, organisational and social level. Again, overlapping codings were cross-referenced to illustrate how often relationships between techno-stressors and resources at different levels were analysed in quantitative studies. Table 4 displays the distribution of technostress creators analysed in relation to environmental and personal resources. Personal resources were investigated most frequently. Most of the environmental resources examined were located at the organisational level. Resources were particularly less often investigated in combination with techno-uncertainty.

#### 3.4.1. Environmental Resources: Social Level

In total, 11 studies (6 quantitative [9,27,55,57,66,67] and 5 qualitative [48,97,99,100,103] studies) examined resources at the social level. These resources mainly referred to social support and leadership, including, e.g., understanding employee differences [100], sharing ideas and best practices [45], being acknowledged as a new worker and benefitting from positive opinions, mindsets, and social persuasion of colleagues [97]. Friendship opportunities at work improved general health and buffered adverse effects of techno-stressors [65]. Moreover, when learning to use new technology, receiving a short introduction by an instructor and being encouraged to ask questions and able to easily access social support by co-workers and their digital literacy were mentioned as helpful resources in qualitative studies [97,103]. Several studies specifically examined different styles of leadership as a resource in dealing with technostress. A good relationship of employees with their supervisors [64] and managerial intervention [99] helped to reduce technostress. Although supervisors’ influence on ICT usage did not significantly reduce technostress, leadership in general did [9]. Empowering leadership was found to buffer the relationship between techno-invasion, but not techno-overload or techno-complexity and emotional exhaustion [55]. Moreover, a positive leadership climate buffered the effect of techno-stressors on job distress [53] and high leader–member exchange did the same in the relationship between communication, system feature overload (but not information overload) and work–family conflict [66].

#### 3.4.2. Environmental Resources: Organisational Level

Resources at the organisational level were identified in a total of 29 of the included studies, of which 6 were qualitative studies [45,98,99,100,101,103] and 23 were quantitative studies [10,15,21,24,27,59,60,62,64,66,68,69,73,75,76,79,80,86,87,89,91,93,96]. Involvement facilitation [10,15,64,73,76,80,87,96] and literacy facilitation [10,59,64,73,75,76,80,87] were most frequently studied, as already considered by Ragu-Nathan et al. [10]. However, in a few of these studies, no or only partially significant effects were found [60,64,96]. Other types of organisational support were also commonly mentioned and found to be valuable resources to reduce technostress or buffer its adverse effects [60,62,68,89]. More specifically, they included, e.g., innovation support, which in turn was positively related to involvement facilitation [13], administrative support [103], provision of adequate resources [45] and organisational support for strengths use [65]. With regard to techno-invasion, high perceived organisational support in work–home boundary management amplified the relationship between daily positive affect and diminished the relationship between daily negative affect and daily partnership satisfaction [93]. Among employees with a high intensity of telework, techno-invasion was not significantly related to strain, whilst a high intensity of teleworking also buffered the negative effect of strain on job satisfaction [86].

More generally, health and well-being programmes were perceived to reduce technostress [100]. Several organisational resources were mentioned by librarians, such as keeping pace with the developments in the market, making prompt decisions and having effective change management plans and considering staff planning while implementing new technologies, realistic time scheduling to avoid multitasking, providing time to implement and learn how to use new technologies and developing and maintaining comprehensive technology standards, effective communication and continuous staff training [45]. Communication, knowledge sharing and training were also described to reduce technostress by logistic managers [100]. A quantitative study among salespeople supported that continuous training programmes for technologies reduced technostress and had a positive effect on the participants’ beliefs about technology [24]. Communication measures were mentioned as valuable resources in several other qualitative studies including different occupational groups [98,100,101,103]. In particular, email culture was emphasised, such as informal, universally known rules about the use of adequate media depending on the situation (e.g., email or phone call) were perceived as helpful [98], or meeting in person instead of writing emails as well as communicating about digital communication with co-workers, e.g., discussing ways to reduce the number of emails [103]. Identifying best practices was further described as a measure to address techno-overload in addition to improved communication with executives and providing ways to find information and support more efficiently. Regulating after-work email traffic and communicating such regulations allow employees to end their working day and thus reduce techno-invasion [101]. Although internal communication was not found to moderate the relationships between techno-overload, techno-invasion, techno-complexity or techno-insecurity and commitment to change, high internal communication buffered the negative effect of techno-uncertainty on commitment to change [91].

Moreover, increased scope for action and a hierarchical, i.e., process-oriented organisational culture [64], human resource management effectiveness [67], transparency and fairness in the distribution of work and a reduced workload [99] were identified as organisational-level resources in dealing with work-related technostress. While perceived technostress was significantly negatively associated with customer satisfaction [21], customer satisfaction was on the other hand perceived to reduce technostress [99]. Against the authors’ expectations, job design, including job autonomy, skill variety, task identity, task significance and task feedback, was found to increase technostress, which again indicate an inverted U-shaped relationship rather than the assumed linear one [78]. Job autonomy was found to be able to reduce strain by reducing perceived invasion of privacy [86]. However, job control, stress management training and individual rewards could neither reduce job stress nor buffer adverse effects of technostress creators on job stress [68].

#### 3.4.3. Environmental Resources: Technical Level

At the technical level, which was addressed by 13 studies, among them 5 qualitative studies [48,97,98,100,103] and 8 quantitative studies [10,64,73,74,76,77,80,87], different kinds of resources were identified. Some of them are also influenced by organisational circumstances or implementation by the organisation. These included improving the technological infrastructure [45] or being able to rely on IT experts [100]. Technical support provision was investigated most frequently [10,64,73,74,76,80,87,103] and the majority of results clearly supported its significant influence as a technostress reducer or mitigator [64,73,74,80,87,103]. In addition, usability and benefits of technologies, e.g., enabling flexibility and automation [97] or facilitating communication and documentation [98], as well as back-up routines [103] were mentioned as resources in dealing with technostress. Reliability of technology, in contrast, was not found to significantly reduce technostress [76].

#### 3.4.4. Personal Resources

Thirty studies covered personal resources of different kinds; most of them were quantitative studies [6,10,21,23,24,46,54,64,70,71,73,74,76,77,80,81,82,83,85,87,89,90,93,94,96]. Only four of them were qualitative studies [97,98,99,103] and one was a mixed-methods study [7]. In particular, different types of self-efficacy [6,23,24,26,56,74,75,77,85,87,89,97] were frequently examined in the studies. Andreou differentiated different sources of self-efficacy that mitigated technostress among new knowledge workers. Being new to the organisation was described to come along with eagerness to learn new things and, thus, positive psychological arousal. Although it took more time to understand it completely, learning to use a new technology individually on their own could therefore be helpful. Thus, mastery experiences helped to mitigate techno-complexity, techno-insecurity and techno-invasion (as long as ICT were not overused and created work–home conflicts). In a similar manner, psychological arousal mitigated techno-overload, techno-complexity and techno-uncertainty and positively impacted self-efficacy. However, too much positive arousal and eagerness caused concentration problems and work–home conflicts. While negative experiences of colleagues impacted new workers negatively, positive vicarious experiences reduced techno-complexity. Likewise, social persuasion by other colleagues could raise existing self-efficacy by creating a positive mindset and psychological arousal, and reduced techno-uncertainty, but when lacking self-efficacy, social persuasion could even create techno-uncertainty [97]. Other studies provided further support that (technology- or job-related) self-efficacy can mitigate negative effects of technostress [6,23,26,56,74,77,85,89]. In addition, continuous techno-training was significantly positively associated with techno-efficacy [24] and technology self-efficacy was significantly positively related to sales performance [87].

Moreover, (IT) mindfulness was found to decrease technostress [72,81,96] and increase user satisfaction [71] as well as decrease job burnout, but it did not significantly buffer the relationship between techno-stressors and job burnout [80]. Job commitment did not buffer the negative relationship between technostress creators and job satisfaction, but the positive association of technostress creators and role stress [83].

High IT control mostly helped to reduce the adverse moderating effects of emotion-focused coping strategies (i.e., distress venting and distancing from IT) on the techno-stressors–strain relationship [81,82]. Likewise, empowerment through control over reachability reduced technostress [99]. IT use autonomy reduced the negative effect of techno-stressors on IT-enabled productivity and simultaneously increased productivity [7]. Having a high degree of control over the boundaries of work and leisure time significantly reduced work–family conflict created by extended availability [94] and mitigated negative effects of high after-hours availability expectations and frequent work-related smartphone use after work on psychological detachment [77]. However, work–home integration was also found to significantly reinforce both, the effect of daily positive and negative affect on partnership satisfaction [93].

Further personal resources reducing or mitigating negative effects of perceived technostress included promotion focus [70], optimism towards technology [21], personal innovative in IT (i.e., the willingness to try out new technologies [105]) [96] or being interested in technology [97], trust in people and processes [99] as well as computer confidence [10], a confident attitude [103] or confidence in dealing with ICT [64]. Moreover, digital literacy was described as a helpful individual competence to deal with technostress in a qualitative study [103], but, just as information literacy, was not found to significantly reduce adverse effects of technostress in a quantitative study [46]. A similarly contradictory result was found by Gimpel et al. in whose study increased digital media literacy was associated with lower perceptions of techno-complexity, but with increased perceptions of other technology-related stressors [64]. Another quantitative study found that technology competence was positively related to technology-enabled innovation and productivity [87].

### 3.5. Appraisal

Only five studies [58,84,92,93,98] addressed appraisal, i.e., the process that decides whether potential stressors are actually perceived as threatening or not [17]. The knowledge of available resources influences the appraisal. Similarly, coping strategies are also dependent on resources [16]. Several authors followed this approach and examined how employees appraised stress caused by email traffic [56,98] and found that framing technology as an opportunity or a threat shaped the consequences of being exposed to technostress creators [84]. Challenge appraisals were associated with problem-focused coping strategies and positive outcomes, while hindrance appraisals were associated with emotion-focused coping strategies and negative outcomes [92,93].

### 3.6. Coping Strategies

In accordance with the theoretical framework, problem- and emotion-focused coping strategies were investigated. Both were examined with comparable frequency. Table 5 displays the frequencies of analysed relationships between coping strategies and techno-stressors for the quantitative studies based on cross-referenced codings.

#### 3.6.1. Problem-Focused Coping Strategies

Twelve of the included studies examined problem-focused coping strategies, of which six were quantitative studies [22,65,66,70,92,95], five were qualitative studies [48,97,100,102,103] and one was a mixed-methods study [7]. They included proactive behaviours such as confronting stressful situations head-on, which was associated with increased productivity and buffered the negative relationship between communication overload and productivity [69]. Other proactive behaviours were coming up with a plan [64] and preparation [103] as well as active actions and efforts to improve the situation [64,95], of which the latter was only partially supported by the data [64]. Another common problem-focused coping strategy was seeking support from others, either instrumental support [92] or support from family and friends [64] or social support from colleagues [95]. However, the latter one could not be supported by quantitative data [95]. These active-functional strategies were also jointly examined (active coping and social support [22] or active coping, planning and seeking instrumental support [63]). Both of these combinations significantly reduced exhaustion [22,63] and buffered [22] or mediated [63] the technostress-exhaustion relationship. Interestingly, older employees seemed to use these strategies more than younger ones and hours of work per day were significantly negatively related to these coping strategies [63].

Moreover, using digital solutions to deal with ICT demands were used to cope with technostress [102,103]. Learning and skill development as successful coping strategies included developing IT use skills [7], learning by doing [103], persevering and learning from mistakes [97]. Structuring and organising were described to be helpful, in particular, time management and prioritisation strategies [100], replying flexibly [103] or only to necessary emails and keeping a record of passwords [45]. Additionally, setting aside time for specific tasks and switching off ICT while working on them were mentioned, which also buffered negative effects of technostress and increased productivity [7]. When dealing with an overwhelming amount of information, looking for summaries and trends, developing dashboards, filtering and being selective about data sources were identified as successful coping strategies [102]. Apart from establishing routines and structures, improvisation was also highlighted as a coping strategy for technostress [103]. Lastly, several studies identified separating work and private life by using separate devices [7,103] and even limiting ICT use outside work [97] as helpful problem-focused coping strategies.

#### 3.6.2. Emotion-Focused Coping Strategies

Emotion-focused coping strategies were addressed by 11 studies [7,22,35,61,65,66,70,82,92,95,102], including only one qualitative study [102] and one mixed-methods study [7]. Four of them examined distress venting [7,35,82] or distress venting and psychological distancing [92] and their results mostly supported the hypothesis that blowing off steam would mitigate adverse outcomes caused by techno-stressors [7,81], but also decreased productivity [7,92]. Distancing from IT was also examined separately in quantitative studies [81,82] and a mixed-methods study [7] and significantly reduced technostress or related adverse outcomes in most of these studies [7,82]. As another type of emotion-focused coping strategies, reframing situations was identified in the included studies, such as looking at the bright side, taking things with humour [64], being optimistic [7] and reinterpreting situations positively [82]. Transforming stressful situations into opportunities, however, was only found to increase productivity, but not to buffer the techno-stressor-productivity relationship [69]. Moreover, some dysfunctional coping strategies were examined. These included strategies characterised by withdrawal [102], disengagement or even denial [61,65,95], ranging from learning to live with the situation [64] to alcohol and drug abuse [22]. However, although dysfunctional coping was associated with increased exhaustion [22,63], it did not reinforce adverse effects of technostress creators on exhaustion [22], but mediated the relationship [63]. Similarly, moral disengagement mediated the relationship between techno-stressors and violating information security policies [59]. Age was significantly negatively correlated with behavioural disengagement, which in turn and together with techno-stressors, mediated the positive correlation between age and technology-related strain [95].

### 3.7. Preventing Work-Related Technostress

As mentioned above, at the time of research, no studies which systematically field-tested interventions or scientifically evaluated prevention concepts at the behavioural or structural level could be identified to answer research questions number three and four. The lack of and need for research on techno-training, coping and interventions has also been addressed by other authors of included studies [7,23,24,26,55,94]. However, as described in Section 3.4.2, some studies examined stress management training [68] or continuous techno-training [24] and some participants addressed trainings for technology and well-being and health programmes [100].

## 4. Discussion

The aim of this scoping review was to provide a comprehensive overview and to gather and map existing empirical findings on preventing and coping with work-related technostress based on the theoretical framework of Bamberg et al. the occupational psychological stress model [16,17]. This review of the current state of research shows that although some findings on resources and coping strategies are available, no preventive measures have been scientifically evaluated yet. Moreover, the global distribution of authorships and studies emphasise the international and cross-cultural relevance of the topic. Likewise, the high amount of studies published particularly in the last two years indicate a rapidly growing interest in the scientific community. Both of these results are consistent with findings from recent scientometric [4] and bibliometric [106] analyses on technostress. In line with a recent systematic review on mental health and work outcomes, strain and stress, burnout and exhaustion as well as satisfaction, performance and productivity were identified as the most frequent outcome measures. Moreover, our results support the review’s finding that techno-overload and techno-invasion were the most frequently examined techno-stressors and that many studies did not examine all of the five techno-stressors [33]. This scoping review further demonstrates that previous research was carried out on a wide variety of occupational groups and thus examined work-related technostress in a wide range of conditions.

### 4.1. The “Dark Side”: Challenges of Work-Related ICT Use

While this scoping review focused on the concept of technostress and the five technostress creators defined by Ragu-Nathan et al. [10], the synthesis reveals that techno-overload, techno-invasion and techno-complexity were most frequently studied in the work context. However, many studies also identified further technology-related stressors and some described organisational stressors. Tarafdar et al. refer to them as technology environmental conditions [5]. Stressful organisational circumstances may interact with techno-stressors and thus reinforce their negative effects [53,56]. However, according to transactional stress theories, stressors are not harmful per se, but only perceived as such depending on an individuals’ appraisal [5,13,17]. Depending on an individuals’ person-related risk-factors, appraisal and available resources in a stressful situation, stressors will be perceived as harmful or not [16,17]. As noticed before [64], person-related risk factors, particularly gender and age, revealed contradictory results. Although women seem to be more prone to report stress, they may have different working conditions that may explain their lower technostress levels in some studies. Another possible explanation could be the distribution of influential factors among the samples, e.g., gender differences in the adoption of technology may not apply to younger employees [10]. Differences in perceiving technostress in terms of age may be related to work experience or organisational tenure [10,107]. Younger employees, such as millennials, who are used to dealing with different media on a daily basis, may on the one hand be less prone to technostress as digital natives, whereas media literacy might be lower among older employees [108]. On the other hand, younger employees may be more easily overloaded as they probably consume more media in their free time than older employees who might benefit from more experience and therefore be less susceptible to (techno-)stress [109]. In the same vein, a systematic review did not identify linear trends between age and technostress perception [110].

Moreover, the quality and quantity of a stressor may determine the perception of technostress. For example, the intensity of ICT use indicated different levels of technostress perception [90] or using emails as a means of communication was perceived ambivalently, as facilitating communication but also leading to overload and a lack of control [98]. Thus, it is important to emphasise that digital technologies also have beneficial properties that may even help to reduce perceived technostress. For example, using different technologies may even reduce techno-overload [101]. Moreover, strain may not be automatically caused by technostress, but may rather depends on the scope of functions and how they are implemented within organisations [99]. In this regard, an organisational climate of innovation was also shown to reduce perceived unreliability of technology while positively affecting user satisfaction and job satisfaction. However, competitiveness and perceived uncertainty may downsize this positive effect. The way of implementation in the organisation is therefore crucial [61].

Similarly to the inverted U-shaped relationship of arousal and performance specified in the Yerkes-Dodson law [111], several authors of the included assumed the curvilinear relationship between technostress and work-related outcomes [6,79,96] which is also supported by Srivastava et al. [112]. Therefore, in line with the occupational psychological stress model [16,17], techno-stressors should be understood as not fundamentally negative or harmful. Rather, the degree of techno-stressors in combination with an individual’s perceived resources seems to be decisive for the appraisal and extent of experienced technostress [16,17,79].

### 4.2. Overcoming the “Dark Side” of ICT

#### 4.2.1. Using Resources against Work-Related Technostress

Resources are not only important with regard to coping [16] with work-related technostress, but can also be considered as a starting point for measures to reduce technostress at different levels. Our results point out the important role of leadership, which can reduce and buffer negative effects of technostress at work [9,55,57,67]. Harris et al. explain their unexpected finding of leader-member exchange amplifying the relationship between information overload and work–family conflict by the possibility that supervisors are important in providing employees with information. Therefore, supervisors’ information sharing in combination with increased output expectations could explain their finding [66]. In contrast, when they are new to the organisation, some employees may not have the courage to ask colleagues for help [97]. Providing organisational support can therefore be a key resource [15,27,48,60,62,68,89,93,103].

At the organisational level, communication measures were identified as another important resource [91,98,100,101,103]. The results provide evidence that availability policies can help employees to mentally switch off from work in their leisure time [77]. However, universal rules may also restrict employees in their flexibility and, thus, in a valuable resource and communication measures may also entail negative effects. For example, shutting down email servers overnight could help to prevent employees from emailing in the evening during their free time, but may instead cause a flood of emails the next morning. Thus, techno-invasion would only be averted at the price of techno-overload as another techno-stressor [101]. Comparably, Delpechitre et al. found that some job resources may also entail further job demands and stress [6]. This highlights the importance of providing resources at the technical level as well, particularly technical support provision [64,73,74,80,87,103], e.g., by providing IT experts [100] or a help desk [62,113]. The importance of choosing the most appropriate means of communication, precise email correspondence, avoiding sending emails outside of working hours, hardware and software equipment was also emphasised in a recent qualitative survey [114]. Further study results affirm the positive influence of perceived organisational ICT support on ICT demands and psychological well-being [115], which were also found in the included studies [60,62,68,89].

At the personal level, particularly many findings on self-efficacy [6,23,24,26,56,74,75,77,85,87,89,97], mindfulness [72,81,96] and control [35,82,99] were identified. Although (IT) mindfulness was not able to buffer the effect of technostress creators on job burnout [80], several studies found support that it reduces technostress and burnout while increasing user satisfaction [72,81,96]. Hence, (IT) mindfulness may not lead to successful coping responses [80] but could nonetheless be helpful to reduce technostress. Previous research suggests that mindfulness and self-efficacy can be trained [116,117,118,119], while different sources of self-efficacy may influence each other [24,120]. Moreover, in line with our results, a recent review by Virone et al. has identified, inter alia, autonomy, time pressure, understanding of roles and attitude as relevant factors for coping with technostress among healthcare employees [121]. Other study results affirm our findings that a promotion focus [122] and data literacy [123] can reduce technostress creators.

#### 4.2.2. Coping with Work-Related Technostress

Following the theoretical framework, problem- and emotion-focused coping strategies [17] were differentiated. Problem-focused and emotion-focused coping strategies were almost equally often examined in the included studies. To reduce work-related technostress, seeking support from others seems to be a promising problem-focused coping strategy [22,65,66,92]. Commonly investigated emotion-focused coping strategies reducing technostress included distress venting and distancing from IT [7,35,82,92]. Similar to IT distancing, digital detoxing behaviours can be helpful to reduce overload resulting from work-related ICT use when working remotely, as research from the COVID-19 pandemic shows [124]. Furthermore, positive reframing of situations was also identified as a coping strategy in a multi-organisational case study [125]. However, coping strategies can further be distinguished as problem-focused, emotion-focused and dysfunctional coping strategies. Accordingly, dysfunctional strategies include behavioural and mental disengagement, denial, venting, and substance abuse [126,127]. Such coping strategies may provide short-term relief, but are often not functional in the long term and can, therefore, even be harmful to the individual [22].

Interestingly, employees seem to apply several coping strategies when experiencing increased technostress. Employees, who coped with technostress in different ways, also rated their health and work ability better and reported less difficulties in mentally detaching from work in their free time than those who only used few coping strategies [64]. Findings from a study among adolescents support the assumption of increasing coping with higher levels of technostress [128]. While Saxena and Lamest were startled by the absence of team-based coping strategies in their case study [102], our results demonstrate that coping strategies are usually examined at the individual level. Although some studies identified social support among colleagues as an important resource in coping with technostress, it was inquired at the individual level [97,103]. However, first studies on dyadic coping among colleagues seem to be emerging [129].

#### 4.2.3. Developing Prevention Measures for Work-Related Technostress

As with a recent scoping review on nurses’ strategies to prevent technostress, no studies on prevention measures or strategies were identified in this scoping review [37]. Given the lack of studies addressing primary, secondary or tertiary prevention or technostress interventions, merely first approaches based on the findings presented in this review can be suggested. Interventions should aim at altering appraisal and coping processes [92]. For example, Gaudioso, Turel and Galimberti suggested training employees in adaptive coping strategies and in being aware which coping strategy they use [63]. Rayburn et al. agree that there is a gap in research on the prevention of technostress through training [24]. Some researchers have already made use of the first findings on technostress mitigation through gamification in e-learning [130] and developed a game-based digital training platform [131], which remains to be scientifically evaluated yet. The gamification approach was also suggested as a prevention measure in a recent research report [113]. In this report, Gimpel et al. introduced 24 approaches to strengthen resources and reduce demands from different techno-stressors and technology environmental conditions [113], providing a catalogue of measures to prevent technostress in the workplace.

### 4.3. Strengths and Limitations

This scoping review followed a systematic approach to summarise and map the current state of research, including the recommended screening and methodological assessment processes carried out independently by several authors [43,45,46]. The high quality of this scoping review is further reflected in the inclusion of multidisciplinary databases, study designs and languages. Following the theoretical framework of an extended transactional stress theory [16,17], this review adopts a model used by most of the relevant studies [4] and is thus in line with the prevailing consensus of leading researchers in this field [5]. Moreover, this scoping review exclusively focused on work-related technostress. Although technostress may also arise from private ICT use, using ICT at work is rather bound to a purpose instead of entertainment [132]. Employees may therefore only have limited or no possibilities to influence their exposure or dose of ICT at work, which highlights the importance of researching prevention and coping options in this context. The review was based on the most widely adopted [133] conceptualisation of technostress and technostress creators [10]. Aiming to comprehensively present the current state of research, identified records were carefully examined and included based on the technostress creators’ definitions by Ragu-Nathan et al. [10], including adapted scales or items adhering to these definitions, which were discussed thoroughly among the authors. Overall, this scoping review contributes a comprehensive overview of the current state of research and identifies starting points for further research and practice.

However, some limitations need to be addressed. Not all studies included all of the five technostress creators. Sometimes, only four dimensions seemed to fit the investigated context [53]. Some studies used the items or adapted them to their specific research questions. Others added or combined them with further technostress creators, which are not included in Ragu-Nathan et al.’s concept [10]. The underlying conceptualisation of technostress creators was developed more than a decade ago; therefore, some authors have extended this concept more recently by further technology-related stressors [64,133], which can partly be referred to as technology environmental conditions [5]. Due to their recentness and thus lower prevalence in already published studies, these newly added factors are not primarily considered in this review. Instead, it focused on the most widely established five techno-stressors [10,106,134].

Included studies represent a large period of time (2008–2021), which may limit the comparability of studies considering technological progress that may contribute to increased technostress. The included studies also represent many different occupational groups, potentially limiting the comparability. However, this inclusive approach was chosen since many studies did not clearly state their inclusion criteria for participation, included a wide range of occupations in their samples or described their samples broadly as “employees using ICT at work”. In contrast, from a transactional perspective, technostress is considered highly contextual [5,14]. Apart from situational specificity, individual perception and appraisal, it may also differ among occupational groups [134] or cultures [53,92]. The examination of different occupational groups, including diverse tasks and job demands, may therefore explain divergent results among the included studies [55]. Moreover, all included studies relied on self-reported data measuring technostress. Despite many of them using reliable and validated scales, using other data measuring technology-induced stress, e.g., bio-physiological or observational data could additionally support and confirm the validity of the data in a mixed-methods approach [63,135]. This, however, is often difficult to realise in terms of feasibility.

While the inter-rater reliability in the screening processes was substantial, indicating well-defined inclusion criteria, it was only moderate in the quality assessment. The partially low degree of criteria fulfilment, especially among the cross-sectional studies, also indicates that the selected checklists may not have been appropriate for the context of the included studies under review. This could be because the checklists of the Joanna Briggs Institute originated from the health and medical sciences context [48,49,50]. However, it could also be attributed to the inclusion of conference papers, assuming they are limited in length and, thus, provide less information compared to journal articles. Ultimately, we strived to provide a comprehensive overview, yet knowing that even the combination of search strings, searching different databases and manual search cannot possibly identify all relevant records or map the state of research exhaustively.

### 4.4. Theoretical and Practical Implications

#### 4.4.1. Implications for Further Research

As our results strongly point out, there is an urgent need for research on specific prevention approaches or the development and evaluation of interventions [53]. The distribution of how often the different techno-stressors were examined in the included studies indicates a need for further research, particularly on techno-insecurity and techno-uncertainty. Similarly, according to the frequencies of the cross-referenced techno-stressors, resources and coping strategies, further resources can be explored at the social and technical level. For the development of interventions, further insights on functional coping strategies and their consequences will be particularly useful. However, as several authors already noted, the positive effects of techno-stressors [5,93] and coping strategies [7,26,27] also need to be further illuminated. Since many of the coping strategies identified in this review included dysfunctional coping strategies, future research should focus on functional coping strategies to promote a healthy approach to technostress. The dearth of research on work-related technostress prevention or particular interventions is reflected in the framework developed by Tarafdar et al. (2019). Presenting their research agenda, they advocate investigating the positive effects of technostress as well as mitigating its negative effects through appropriate technology design. Information systems design features may be applied to support coping and positive, or to diminish negative, aspects of techno-stressors and outcomes [5]. Whether technology use may also lead to techno-eustress will need to be further researched in the future [5,22].

As different authors stated before, future research could focus more on organisational mechanisms and approaches to reduce technostress besides the already investigated technostress inhibitors [68,71]. However, as in this scoping review, different levels should therefore be considered [22]. At the organisational level, further resources and opportunities for interventions need to be identified and their effects further explored to foster structural prevention approaches. Regarding work design, not only the consideration of different factors at the respective levels, but also possible interactions should be taken into account [64]. Moreover, the impact of organisational culture should be further examined [79] due to the paucity of research in relation to technostress [63,66,75,88]. At the individual level, more research is needed on employees’ coping with technostress [10,81] and on possible interdependencies of different coping mechanisms [81].

Furthermore, Benlian criticised the static concept of technostress, calling for a more dynamic approach that could also account for within-person processes [93]. Therefore, as supported by the large amount of cross-sectional study designs among the included studies in this scoping review, several authors [22,24,55,79,93], have already called for more longitudinal studies to be conducted in the future. Longitudinal studies could provide insight into the extent to which the use of coping strategies affects individuals’ resources over time [95] and offer important implications for the design of interventions or could be used for pre–post analyses when introducing new technologies [10]. Nevertheless, interdisciplinary research remains important to gain a deeper understanding of positive and negative consequences of technostress as well as how to mitigate adverse effects [5,22]. Since technostress can be considered a “cross-domain phenomenon” [93] (p. 1278), future research should not only examine technostress from multidisciplinary perspectives, but it should also draw on different measurements [5] and sources, such as supervisors or family members in addition to employees [23,93] or group-level analyses [53]. Overall, the findings, particularly on techno-invasion and work–family conflict, provide evidence that work-related technostress and its effects reach far beyond the work sphere. They also impact employees’ private life. Therefore, they need to be examined in both spheres and, consequently, be counteracted with holistic approaches. In this vein, emerging research also considers the influence of personality traits in the context of technostress perception [112,136,137,138].

In the past two years, working conditions have changed significantly due to the COVID-19 pandemic and work with ICT has increased as a result [139]. The now widespread possibility to work remotely puts workplace health promotion, particularly dealing with techno-invasion, in the spotlight. Preliminary study results indicate higher technostress levels during the COVID-19 pandemic compared to before [140], but a decrease among employees who were already accustomed to the use of ICT pre-pandemic [141]. In a study where remote work during the COVID-19 pandemic was negatively related to technostress, remote work was also positively associated with flow at work [142]. Supporting the notion that leadership can serve both, as a potential stressor or resource [53,143], authoritarian leadership was found to have an either enhancing (when high) or protective (when low) effect on the workaholism–technostress relationship among completely remotely working employees during the pandemic, depending on its degree of expression [144]. Job crafting and organisational communication could be further protective factors (i.e., environmental resources) when experiencing technostress while working remotely [145,146]. Significant relations were found between working conditions (i.e., technical equipment) and perceived technostress, which also became apparent in blood cortisol levels [147]. Moreover, while other technology-related stressors such as techno-unreliability gain in importance during remote work in the COVID-19 pandemic [148], other strain reactions, e.g., techno-fatigue, emerge [149,150] and require newly developed behavioural and structural prevention approaches. Due to this unforeseen and substantial change in working conditions, studies related to preventing and coping with work-related technostress due to remote work during COVID-19 should be addressed in a separate review once a sufficient database is provided.

#### 4.4.2. Implications for Practice

The findings further provide some implications for organisations to prevent and support employees in coping with work-related technostress. For this purpose, prevention measures can be subdivided into behavioural and structural prevention measures.

At the behavioural level, an initial important step for organisations is to support employees to adopt functional coping mechanisms [151] by educating them about possible coping behaviour based on the results presented in this review, thus providing them with different options for action to engage in. Starting from there, organisations can offer trainings for employees to develop IT competencies [113] and individual coping behaviours. Regarding personal resources to reduce work-related technostress, trainings could also strengthen mindfulness [80,116], self-efficacy [23,117,118,119] and IT control [35,82,99]. Stress management techniques can help to counteract irritation and stress [72]. As is evident from our results, although it might not address the root of the problem or be beneficial in the long run, emotional coping such as distress venting can reduce technostress effectively [81]. Employees should be encouraged to share their coping strategies and experiences among colleagues to increase benefit and be motivated to try out different strategies. However, dysfunctional coping strategies such as alcohol consumption that reduce technostress in the short term can have serious consequences in the medium or long term that may even exceed the consequences of permanently experienced technostress. In this regard, organisations should support the application of functional coping strategies among employees wherever possible [22].

Assistive technology can also be used to promote healthy behaviours and trainings could support employees to improve their self- and time management, sensitise and promote self-reflection about the causes, effects and consequences of technostress and one’s own way of working and managers to lead digitally [113]. Given that individual employees within an organisation require individual strategies, flexible IT use policies, e.g., email management strategies, might help employees to adopt various coping strategies rather than generalising measures such as shutting down servers [101]. Monotasking and taking breaks during the work day or reducing ICT use in leisure time may help to gain distance from digital demands [78,97,113]. Furthermore, it might be helpful to use ICT selectively, i.e., to use ICT only when it is functionally sensible and appropriate to do so [45]. Overall, employees and managers need to discover their personal healthy boundaries of ICT use [97] and understand that coping with technostress also relates to a sound ICT use in private life [7]. Especially when it comes to availability expectations, perceived techno-invasion may not merely be encouraged by ICT design but also by peer influence [132], i.e., managers’ and colleagues’ expectations and behaviours. Moreover, technostress mitigation requires self-regulation and can therefore impede health-promoting behaviour [132], e.g., resist checking emails after work, knowing colleagues may be doing so. Adjusting expectations regarding email response times, developing and complying with clear corporate guidelines on availability expectations as well as personal rules and guidelines regarding ICT use at and outside of work may help to cope with technostress [114]. Managers should appeal to employees’ self-responsibility in terms of ICT use and reconciling work and private life [114] and properly delegate tasks to reduce their own technostress [88]. In this vein, a balanced combination of autonomy and control based on the individual employee’s needs is required [152]. Regarding distressing work-related social media use, it might be helpful for managers to draft specific policies [58], ideally in cooperation with their employees, e.g., establishing team norms and a shared understanding of when, why and how employees are available for work-related communication [113]. Interventions aiming at improving psychological detachment from work may also be helpful in this context [79].

Within organisations, multipliers who pass on information on preventing and coping with work-related technostress to employees could be managers. At the same time, with regard to health-oriented leadership, managers should always pursue the two directions of leadership, i.e., self-directed health-oriented leadership (SelfCare) and follower-directed health-oriented leadership (StaffCare) [153]. In this dual role, while managers seem to be more susceptible to techno-overload and techno-invasion than employees [154,155], they need to act according to their role model function [156] and as positive social influencers [157] to protect themselves and their subordinates, e.g., when dealing with technology and availability expectations. Through their own understanding and practice of dealing with ICT, managers could support employees and counteract harmful developments [154]. However, dealing with availability can be subject to individual preferences of integration or segmentation of work and private life [158]. Possibilities for availability rules, demarcation and self-organisation should therefore be consciously reflected upon by managers and employees and incorporated into workplace health management [154]. 

Interventions aiming at non-directive leadership styles could promote employees’ resources [55]. Moreover, it should be noted that empowering leadership could also increase the burden of emotional exhaustion in employees. Therefore, managers should be careful not to burden employees when autonomy and responsibility are rather perceived as overburdening [55,159]. This again underlines the individuality of appraisal and coping processes of employees and managers. Managers should provide employees merely with necessary or relevant information to prevent information and communication overload [6] and state clear role expectations for employees to reduce role ambiguity, especially when new technologies are implemented. In this case, adequate IT infrastructure and sufficient information in case of technology breakdowns for employees should be ensured [21]. Organisations should also offer ongoing training, managerial and technological support in digital change processes [113]. Trainings may also serve to develop an understanding and appreciation for (new) technology and to increase the effectiveness of change management processes. In this vein, Kwanya et al. recommend individual trainings [45]. It is also recommended to regularly foster education on new technologies and to prevent resistance to technological change as an emotional process which might counteract efforts to mitigate technostress [24,72]. Moreover, training may improve confidence in using ICT [85]. With regard to involvement facilitation and literacy facilitation, managers could support and reward using newly introduced ICT as well as sharing this knowledge among team members [62]. Organisations should provide training for employees and managers that does not only meet their demands [154], but also aims at compensating deficits (e.g., in digital media literacy), and focuses on individual strengths [55]. This strengthening of resources may counteract negative spirals, as suggested in the occupational psychological stress model [17] or Hobfoll’s conservation of resources theory [160]. Accordingly, a combination of a stressful work environment and low resources can lead to a self-reinforcing stress spiral of stressors and stress consequences [17]. Similarly, initial resource loss of lack of resources may cause a loss cycle. Additionally, while resources are needed to recover from or protect against such a loss, resource loss will be disproportionately more salient than a resource gain [160]. Therefore, the implementation of resource-strengthening interventions should be targeted. In this vein, Goetz and Boehm suggest that teambuilding events could facilitate friendship opportunities among colleagues [65], thus strengthening the environmental resource of social support at work.

While interventions should be implemented at both the individual and the organisational level [79], counteracting some techno-stressors may require a more general, organisational-level approach. In a structural approach to prevention, organisations should seek to keep the demands on their employees as manageable as possible since the literature suggests that both too low and too high levels could be damaging [22]. Moreover, it should be considered that adverse effects of work-related technostress may also negatively impact customer satisfaction and relationships [21,84]. A central approach to avoid technostress in a primary preventive way is the design of the technology [5,113,155]. Therefore, to prevent work-related technostress in its genesis, an adequate IT infrastructure needs to be built and maintained [113]. A technological infrastructure allowing for collaborative teaching and learning could further contribute to technostress reduction within the organisation and among colleagues [72]. Moreover, allowing employees to choose technologies they assume to fit best for their tasks apart from mandatory ICT could increase their perceived control [81]. From an organisational and work design perspective, not only do various factors, e.g., work organisation, work environment and work equipment, need to be considered to prevent technostress, but also the interaction of technological and organisational factors [64]. According to the technology acceptance model, acceptance can be increased through perceived usefulness and ease of use [161]. These can be achieved, for example, through an exchange between software developers and users [103] and clarified in training courses to thwart technostress. Recent study results support that usability, i.e., reliability, usefulness and ease of use, can reduce techno-overload and IT-related strain [162]. Regarding technology acceptance and adoption, task–technology fit [163] and possible interactions between task and organisational processes, attributes of technology and the individual using it should thus be considered in prevention [154,164]. Organisations can therefore use these theories for assessments before implementing new technologies and for evaluations [151]. Especially in digital change processes, procedures should be adapted preventively, necessary competences should be developed and software or experts should be provided to prepare relevant information for employees in a comprehensible and user-friendly way. Thereby, a needs-based competence development can reduce employees’ techno-uncertainty. Jager and Thiemann also highlight the importance of quickly available competent experts for technical problems [154].

When implementing strategies for prevention at the organisational level, the practicability and cost–benefit ratio should be assessed in advance since mitigation strategies may also have adverse effects [101]. This point also includes the fact that organisational-level mitigation or prevention measures further need to take into account different individual needs of employees. However, changing the job design alone might not be sufficient to mitigate technostress, if other factors such as working conditions and technical aspects are not considered. Hence, a more holistic, sociotechnical approach is advisable when redesigning jobs and tasks [78]. Holistic approaches may include peer-to-peer or supervisor-to-employee coaching and mentoring to cope with techno-stressors [93]. Thereby, although mitigation strategies should be implemented techno-stressor-specific [165], technostress countermeasures do not necessarily be technology-specific [60]. Organisational measures such as flexible working times and break sequences as well as opportunities for exchange could further contribute to building social bonds and reducing techno-uncertainty [65,113]. Moreover, resources can be strengthened by developing a cooperative corporate culture and a mission statement on communication [113]. Nevertheless, employees’ private ICT use also needs to be taken into account when taking a holistic approach to technostress prevention. As Pirkkalainen, Salo, Makkonen and Tarafdar [81] stated, “technostress-creating conditions cannot be fully prevented in workplaces” (p. 13). Therefore, personal development should be encouraged and supported [81].

Beyond the individual level, organisational and technological (infra-)structures as well as the legal framework need to be adapted to digital working environments [166,167]. Where laws are not (yet) effective, company regulations are needed to protect employees. It is therefore necessary to incorporate into law that mental stress caused by techno-stressors must be avoided in terms of occupational health and safety (OSH) and that the Working Hours Acts also apply in the digital work context. At the same time, ICT can also support OSH activities [168]. For a comprehensive prevention of technostress, techno-stressors need to be considered in and become an inevitable part of psychosocial risk assessments at the workplace [31,81,155]. Therefore, relevant techno-stressors should be identified and individual prevention measures which meet employees’ demands should be derived. After participatory implementation, the prevention measures should be evaluated and, if necessary, adapted to ensure their sustainable effectiveness [113]. With regard to flexible digital work, raising awareness and involving employees themselves in OSH is becoming more important [168]. In addition to OSH experts, not only should the individual needs of employees be taken into account, but also their expertise with regard to techno-stressors at their workplaces [169]. In light of increasing responsibility for their own health, employees’ health literacy needs to be fostered as part of a sustainable prevention culture [167].

## 5. Conclusions

Given the need for an interdisciplinary investigation of technostress, this scoping review links information systems and psychological stress research. While most studies on technostress examined its causes and (adverse) consequences, this review focused on approaches for preventing and coping with work-related technostress. The review provides a comprehensive overview of the current state of research by mapping environmental resources as well as personal resources, problem- and emotion-focused coping strategies to reduce work-related technostress and its potential work- and health-related consequences. Despite a growing body of research on mitigation of technostress, there are no targeted interventions or evaluations of prevention measures yet. Many of the examined resources and coping strategies provide starting points for behavioural prevention measures. However, to overcome work-related technostress comprehensively, an interaction of both behavioural and structural prevention measures will be necessary. Particularly, techno-stressors should be incorporated in psychosocial risk assessments to derive appropriate prevention measures at different levels. Employees and managers should be supported in developing functional coping strategies to deal with work-related technostress. Therefore, to overcome the “dark side” of technology, future research still needs to focus more on the “bright side” of preventing and coping with adverse consequences technostress and examining its positive effects.

## Figures and Tables

**Table 1:** Eligibility criteria.

	Inclusion Criteria	Exclusion Criteria
Participants	Employees and managers	Self-employed workers Non-work-related samples Student samples
Concept	Technostress based on Ragu-Nathan et al. (2008) [10]	Other stressors not related to concept of technostress [10]
Context	Workplace	Private context (e.g., private use of ICT)
Outcome	Health- and work-related outcomes	Other outcomes than health- or work-related
Types of evidence sources	Primary research studies (qualitative, quantitative, or mixed-methods) Conference papers Research reports Theses and dissertations	Non-empirical publications (e.g., letters, editorials) Reviews and meta-analyses Experimental studies (laboratory settings)
Languages	English, German	Other languages
Period	2008–2021	Before 2008, from 2021 ^1^

^1^ The cut-off date for the search was 20 August 2021.

**Table 2:** Distribution of studies according to countries of implementation or first author location.

Countries	*n*	%
USA	17	27
Germany	11	17
China ^1^	10	10
Korea	5	8
Finland	3	5
UK	3	5
Sweden	2	3
Canada	2	3
The Netherlands	1	2
Italy	1	2
Kenya	1	2
Norway	1	2
India	1	2
Nigeria	1	2
Ireland	1	2
Turkey	1	2
Malaysia	1	2
Australia	1	2
South Africa	1	2
Different European countries ^2^	1	2
Different countries worldwide ^2^	1	2

Note. If no information on the study location was provided, the first author’s location is given. The total amount of *n* = 66 results from four studies containing either two samples or participants from different countries [7,45,53]. ^1^ Including Taiwan (*n* = 4). ^2^ The exact countries were not explicitly mentioned by the authors.

**Table 3:** Frequencies of techno-stressors, work- and health-related outcomes in quantitative studies (*n* = 54) ^1^.

Techno-Stressor	Work-Related Outcomes	Health-Related Outcomes	Total
Techno-Overload	25 (55.6%)	27 (60.0%)	45 (83.3%)
Techno-Invasion	21 (38.9%)	24 (44.4%)	40 (74.1%)
Techno-Complexity	20 (37.0%)	23 (42.6%)	39 (72.2%)
Techno-Insecurity	17 (31.5%)	18 (33.3%)	31 (57.4%)
Techno-Uncertainty	16 (29.6%)	18 (33.3%)	32 (59.3%)
Total	35 (64.8%)	40 (74.1%)	54 (100.0%)

^1^ Including the quantitative part of the mixed-methods study [7], excluding one study which did not provide sufficient information, not on request either [46].

**Table 4:** Frequencies of techno-stressors and resources in quantitative studies (*n* = 54) ^1^.

Techno-Stressor	Social Level Resources	Organisational Level Resources	Technical Level Resources	Personal Resources	Total
Techno-Overload	3 (5.6%)	13 (24.1%)	6 (11.1%)	13 (24.1%)	45 (83.3%)
Techno-Invasion	2 (3.7%)	12 (22.2%)	5 (9.3%)	15 (27.8%)	40 (74.1%)
Techno-Complexity	2 (3.7%)	12 (22.2%)	6 (11.1%)	14 (25.9%)	39 (72.2%)
Techno-Insecurity	2 3.7%)	10 (18.5%)	6 (11.1%)	12(22.2%)	31 (57.4%)
Techno-Uncertainty	1 (1.9%)	10 (18.5%)	5 (9.3%)	9 (16.7%)	32 (59.3%)
Total	6 (11.1%)	23 (42.6%)	8 (14.8%)	26 (48.1%)	54 (100.0%)

^1^ Including the quantitative part of the mixed-methods study [7], excluding one study which did not provide sufficient information, not on request either [46].

**Table 5:** Frequencies of techno-stressors and coping strategies in quantitative studies (*n* = 54) ^1^.

Techno-Stressor	Problem-Focused Coping Strategies	Emotion-Focused Coping Strategies	Total
Techno-Overload	5 (9.3%)	7 (13.0%)	45 (83.3%)
Techno-Invasion	4 (7.4%)	5 (9.3%)	40 (74.1%)
Techno-Complexity	3 (5.6%)	5 (9.3%)	39 (72.2%)
Techno-Insecurity	3 (5.6%)	4 (7.4%)	31 (57.4%)
Techno-Uncertainty	3 (5.6%)	5 (9.3%)	32 (59.3%)
Total	7 (13.0%)	10 (18.5%)	54 (100.0%)

^1^ Including the quantitative part of the mixed-methods study [7], excluding one study which did not provide sufficient information, not on request either [46].

## Supplementary Materials

The following are available online at https://www.mdpi.com/article/10.3390/ijerph19063625/s1, Table S1: Search strategy for the medical database PubMed; Table S2: Search strategy for the medical database MEDLINE; Table S3: Search strategy for the psychological database PSYNDEX; Table S4: Search strategy for the psychological database PsycInfo; Table S5: Search strategy for the interdisciplinary database Web of Science; Table S6: Charting the data: information on included studies based on Arksey and O’Malley (2005); Table S7: Critical Appraisal of Qualitative Studies; Table S8: Critical Appraisal of Longitudinal Studies; Table S9: Critical Appraisal of Cross-Sectional Studies.
