# Supplementary material for: Overcoming the “Dark Side” of Technology—A Scoping Review on Preventing and Coping with Work-Related Technostress
Source: Int J Environ Res Public Health. 2022 Mar 18;19(6):3625. doi: 10.3390/ijerph19063625 (PMC8954507; doi:10.3390/ijerph19063625)
Supplement: Supplementary file 1 [file ijerph-19-03625-s001.zip › Supplementary Table S6.pdf]

Table S6. Charting the data: information on included studies based on Arksey and O'Malley (2005).

Author(s), Year of Publication, Study Location(s)	Publication Type, Methodological Approach, Study Design(s)	Sample Size and Population	Study Aim(s)	Main measurements	Important Results
Alnakhli (2019), USA	Dissertation, quantitative, cross-sectional survey	381 salespeople	• to explain why some salespeople perceive technostress and how they react to it	Independent variables:<br>• role overload<br>• use of social media<br>• social media-induced technostress<br>Moderators:<br>• technological self-efficacy<br>• gender<br>Mediators:<br>• work exhaustion<br>• role overload<br>Outcomes:<br>• work exhaustion<br>• social media-induced technostress<br>• job satisfaction<br>• role overload	• role overload was positively related to work exhaustion (β = .45; $p < .001$)<br>• role overload was negatively related to job satisfaction (β = -.24; $p < .001$)<br>• social media technology use was positively related to social media-induced technostress (β = .17; $p < .001$)<br>• social media-induced technostress was positively related to role overload (β = .48; $p < .001$)<br>• social media-induced technostress was positively related to work exhaustion (β = .36; $p < .001$)<br>• there was a significant difference among males and females in perceiving role overload due to social media incused-technostress (β = .19; $p < .001$)<br>• there was no significant difference between males and females in perceiving work exhaustion due to social media-induced technostress (β = .088; n.s.)<br>• salesperson technology self-efficacy negatively moderated the relationship between social media-induced technostress and work exhaustion (β = -.15; $p < .05$)

Author(s), Year of Publication, Study Location(s)	Publication Type, Methodological Approach, Study Design(s)	Sample Size and Population	Study Aim(s)	Main measurements	Important Results
Andreou (2019), Finland	Master's Thesis, qualitative, semi-structured interviews	16 (new) knowledge workers	• to examine the relationship between technostress and perceived self-efficacy in new knowledge workers and how it is manifested in the beginning of the employment	• benefits of technology mitigate technostress<br>• usability of technology mitigates technostress<br>• acknowledgement of being a new worker<br>• vicarious experiences (positive opinions of colleagues shape own)<br>• positive social persuasion by colleagues<br>• positive mind-sets of colleagues<br>• social support (providing help, being accessible, getting short introduction when learning new technology, learning new technology by doing with instructor, encouragement to ask questions)<br>• learning to use new technology individually<br>• being interested in technology<br>• perceived self-efficacy (positive vicarious experiences, positive	• simply being a new worker had a major positive effect → eager to learn new things and found new things very exciting, which resulted to a positive boost, and generally made employees more resistant and create perseverance in them when faced with difficult tasks → this positive psychological arousal reduced techno-complexity, techno-uncertainty, and techno-overload<br>• higher perceived self-efficacy levels with technology in new knowledge workers was associated with lower levels of technostress<br>• visuality, quickness and pleasant user experience of a technology had an impact on technostress mitigation: the more pleasant, easy to use and quick the technology was, the less technostress it created<br>• being a new worker was more forgiving by having looser requirements and more time for learning: the interviewees felt, for example, that they did not necessarily need to know how to use a certain system yet fully because of their novelty in the organisation

Author(s), Year of Publication, Study Location(s)	Publication Type, Methodological Approach, Study Design(s)	Sample Size and Population	Study Aim(s)	Main measurements	Important Results
				psychological arousal, mastery experiences)<br>• setting limits to technology use outside work<br>• perseverance / learning from failures<br>• instructions to new technologies are presented too fast<br>• timely restrictions when learning how to use new technology<br>• technical problems caused by third partly / no influence<br>• vicarious experiences: negative opinions of colleagues shape own<br>• too much positive arousal / eagerness<br>• negative psychological arousal/state<br>• negative vicarious experiences<br>• negative attitude towards technology<br>• low self-efficacy	• a short introduction to a technology, where an instructor used the technology first explaining their use simultaneously, and then the interviewee would independently get to use the technology without supervision → quicker and better way to learn a technology than the first way and generated a better mastery experience<br>• vicarious experiences can serve as a great technostress mitigating factor if the new technology taught is simple enough to be learned only by observing → in these cases, vicarious experiences effectively mitigated techno-complexity<br>• vicarious experiences were not effective enough in some cases, when technology is complicated in nature → mastery experience was needed in addition to get an assurance of your abilities and raise the level of perceived self-efficacy (solely relying on vicarious experiences in cases where it is not enough can lead to techno-uncertainty)<br>• a simultaneous combination of vicarious and mastery experiences: the instructor would instruct the interviewee on how to use a technology while the interviewee

Author(s), Year of Publication, Study Location(s)	Publication Type, Methodological Approach, Study Design(s)	Sample Size and Population	Study Aim(s)	Main measurements	Important Results
				• dissatisfaction with information systems<br>• decreased innovation<br>• reduced productivity while using technology<br>• reduced commitment to goals and values<br>• job dissatisfaction<br>• work-home conflict	themselves would be using the system simultaneously → best way to gain mastery experiences, given time and the chance to copy the instructor's actions in the system simultaneously while the instructor is present and keeps the control of the introduction<br>• positive social persuasion helped in creating a positive mind-set and psychological arousal and helped in raising the level of perceived self-efficacy and mitigating technostress<br>• especially in situations where the interviewees had experienced small failures or setbacks in using technology → social persuasion acted as a positive boost in these situations helping in recovering from the setback<br>• when the new worker had a reason to believe that they were capable of doing a task, social persuasion was found to be helpful in some cases raising their perceived self-efficacy → mitigation against techno-uncertainty because the employee might be uncertain of their capability of performing the task<br>• if the new worker did not have any reason to believe that they would be able

Author(s), Year of Publication, Study Location(s)	Publication Type, Methodological Approach, Study Design(s)	Sample Size and Population	Study Aim(s)	Main measurements	Important Results
					to perform the task, social persuasion had the opposite effect in some cases, it created techno-uncertainty → social persuasion can be an empowering experience, help recover from failures and setbacks and create a positive psychological arousal further reinforcing perceived self-efficacy<br>• other people's positive mind-set made the interviewees feel more positive as well and helped them in entering the organisational culture → 'Fake it till you make it' thinking was explained as actively keeping up a positive mind-set which then subconsciously drives the interviewee to be more positive as well and look for alternative solutions when faced with setbacks<br>• being very interested in technology in general was brought up by three interviewees as a mitigating factor: their understanding of technology and its nature of not always working helped in lessening the negative effect experienced<br>• positive psychological arousal made them less prone to technostress by creating a feeling of being more

Author(s), Year of Publication, Study Location(s)	Publication Type, Methodological Approach, Study Design(s)	Sample Size and Population	Study Aim(s)	Main measurements	Important Results
					resourceful, patient, receptive and generally happier<br>• the positive psychological arousal energised them to try different things in the new systems and encouraged them to ask questions when faced with problems → encouraged them to gain mastery and vicarious experiences<br>• too much positivity might go too far, one can become too reckless with work → might negatively affect concentration ability, which may lead to mistakes with technology use or other problems, or emerge as too much eagerness, which lead work-home conflicts<br>• negative psychological arousal (caused by factors happening outside of work, other people's attitudes, or the feeling of hurry) showed to mainly make interviewees more prone to technostress → they were less resourceful, patient, felt unhappier and were far less perseverant: small setback or failure can trigger negative stress reaction when in negative psychological state<br>• combining multiple perceived self-efficacy creators at once was much more efficient in creating perceived self-

Author(s), Year of Publication, Study Location(s)	Publication Type, Methodological Approach, Study Design(s)	Sample Size and Population	Study Aim(s)	Main measurements	Important Results
					efficacy, than any single creator on its own: first provide the employee with vicarious experiences and mastery experiences combined, and then provide social support and persuasion, if seen necessary, while the whole process is boosted by a positive psychological arousal<br>• mastery experiences showed to have a mitigating relationship towards technostress: employees who had mastery experiences from a certain technology versus employees who did not have or had very little mastery experiences from the same technology, comparably were less prone to technostress from that technology<br>• social support in the context of new workers means that the instructor is able to provide help when asked and makes themselves easily accessible (i.e. being next to the employee ready to help) → threshold was low for the new workers to ask for help when faced with technological challenges<br>• social support provided a mastery or vicarious experience, depending on how the help was then actually provided

Author(s), Year of Publication, Study Location(s)	Publication Type, Methodological Approach, Study Design(s)	Sample Size and Population	Study Aim(s)	Main measurements	Important Results
					• getting to know a technology without any introduction on how to use it (i.e. mastery experience) helped in learning the technology better than if someone would have only told them how to use it without letting them try it for themselves, but it also took more time to fully comprehend the technology<br>• knowing how to use a technology might raise the level of perceived self-efficacy with the technology, but ultimately not using a technology in a healthy manner results in technostress → discovering the individual's healthy limits of using a technology is beneficial and provides more information from which technostress mitigation actions can be based on → mastery experiences possibly create techno-invasion but can also help in mitigating it<br>• work-home conflicts caused by technostress were solved by setting limits to technology use outside of work environment<br>• if a technical problem was caused by a third party, like unreliable internet service provider, and could not be fixed

Author(s), Year of Publication, Study Location(s)	Publication Type, Methodological Approach, Study Design(s)	Sample Size and Population	Study Aim(s)	Main measurements	Important Results
					by the interviewee themselves, it caused more technostress<br>• if the technical problem was out of the employees' realm to fix, it caused much less technostress<br>• when the interviewees had generated a low self-efficacy and negative attitude towards a technology, they did not want to use it, only did it reluctantly and kept the negative attitude towards the technology → then reported technostress<br>• the negative effect of reduced job satisfaction did not appear in the study → no previous experience in job position as new knowledge workers and/or potential positive boost a new job position<br>• the most significant negative effects included dissatisfaction with the information systems used (~88%) and reduced productivity (~81%)
Bauwens, Denissen, Beurden, & Coun (2021), Netherlands	Published article, quantitative, cross-sectional survey	339 childcare workers	• to investigate how technostress affects quality of care delivered while considering emotional exhaustion and	Independent variables:<br>• techno-overload<br>• techno-invasion<br>• techno-complexity<br>• emotional exhaustion<br>Mediator:<br>• emotional exhaustion	• techno-overload predicted emotional exhaustion ($B = .175$; $p \leqslant .001$)<br>• techno-invasion predicted emotional exhaustion ($B = .218$; $p \leqslant .001$)<br>• techno-complexity did not significantly predict emotional exhaustion ($B = .062$; $p = .051$)

Author(s), Year of Publication, Study Location(s)	Publication Type, Methodological Approach, Study Design(s)	Sample Size and Population	Study Aim(s)	Main measurements	Important Results
			empowering leadership	Moderator:<br>• empowering leadership<br>Outcome:<br>• emotional exhaustion<br>• quality of care delivered	• emotional exhaustion predicted quality of care delivered (*B* = −.837; $p \leqslant .001$)<br>• the interaction between techno-invasion and empowering leadership was significantly related to emotional exhaustion (*B* = −.129; $p \leqslant .001$).<br>• the interaction between techno-overload and empowering leadership was not found in the hypothesized direction (*B* = .065; $p \leqslant .050$)<br>• no significant interaction effect was found for techno-complexity and empowering leadership (*B* = .062; *p* = .051)
Becker, Derra, Regal, & Kühlmann (2021), Germany	Published article, quantitative, cross-sectional survey	3,362 knowledge workers	• to examine how employees mitigate technostress by analysing active-functional and dysfunctional coping strategies	Independent variables:<br>• techno-complexity<br>• techno-insecurity<br>• interruptions<br>• techno-invasion<br>• techno-overload<br>• techno-uncertainty<br>• unreliability<br>• active-functional coping<br>• dysfunctional coping<br>Mediator:<br>• exhaustion<br>Moderators:<br>• active-functional coping	• job demands (techno-overload, techno-invasion, techno-complexity, techno-insecurity, techno-uncertainty, interruptions and unreliability) were not significantly associated with employees' productivity (*c* = .01 (.03); *z* = .57; *p* = .568)<br>• exhaustion significantly mediated the relationship between job demands and productivity ((*ab* = −.11 (.02); *z* = −7.61; *p* < .001))<br>• active-functional coping was significantly negatively related to exhaustion (β = -.05; $p < .05$)

Author(s), Year of Publication, Study Location(s)	Publication Type, Methodological Approach, Study Design(s)	Sample Size and Population	Study Aim(s)	Main measurements	Important Results
				• dysfunctional coping<br>Outcome:<br>• exhaustion<br>• productivity	• dysfunctional coping was significantly positively related to exhaustion (β = .31; p < .001)<br>• active-functional coping significantly moderated (buffered) the relationship between job demands and exhaustion (β = -.05; p < .01)<br>• dysfunctional coping exhaustion significantly moderated (buffered) the relationship between job demands and exhaustion (β = -.12; p < .001)
Benlian (2020), Germany	Published article, quantitative, longitudinal daily survey	115 employees	• to propose a broader conceptualization of technology-related stressors, referred to as technology-driven stressors, which in turn combine technology-driven challenge and hindrance stressors	Independent variables:<br>• negative affect<br>• positive affect<br>• technology-driven hindrance stressors<br>• technology-driven challenge stressors<br>Moderators:<br>• perceived organisational support in work-home boundary management<br>• work-home role integration<br>Mediators:<br>• negative affect<br>• positive affect<br>Outcomes:	• on days when employees encountered more technology-driven challenge stressors at work, they experienced higher positive affect compared to days when they experienced less technology-driven challenge stressors (γ = .59; p < .01)<br>• on days when employees encountered more technology-driven hindrance stressors at work, they experienced higher negative affect compared to days when they experienced less technology-driven hindrance stressors (γ = .40; p < .01)<br>• on days when employees experienced high positive affect at work, they reported higher partnership satisfaction

Author(s), Year of Publication, Study Location(s)	Publication Type, Methodological Approach, Study Design(s)	Sample Size and Population	Study Aim(s)	Main measurements	Important Results
				• partnership satisfaction<br>• negative affect<br>• positive affect	at home for that day compared to days when they experienced low daily positive affect ($\gamma = .17$; $p < .01$)<br>• daily positive affect mediated the relationship between technology-driven challenge stressors and daily partnership satisfaction, such that technology-driven challenge stressors were positively and indirectly related to daily partnership satisfaction through daily positive affect ($\gamma = .09$; $p < .05$)<br>• on days when employees experienced high negative affect at work, they reported lower partnership satisfaction at home for that day compared to days when they experienced low daily negative affect ($\gamma = -.17$; $p < .01$)<br>• daily negative affect mediated the relationship between technology-driven hindrance stressors and daily partnership satisfaction, such that technology-driven hindrance stressors were negatively and indirectly related to daily partnership satisfaction through daily negative affect ($\gamma = -.11$; $p < .05$)<br>• work-home role integration strengthened the effect of daily positive affect on daily partnership satisfaction such that the

Author(s), Year of Publication, Study Location(s)	Publication Type, Methodological Approach, Study Design(s)	Sample Size and Population	Study Aim(s)	Main measurements	Important Results
					effect of daily positive affect on daily partnership satisfaction was stronger for employees who were more rather than less work–home role-integrated (γ = .05; p < .01)<br>• work-home role integration strengthened the effect of daily negative affect on daily partnership satisfaction such that the effect of daily negative affect on daily partnership satisfaction was stronger for employees who were more rather than less work–home role-integrated (γ = -.05; p < .05)<br>• perceived organisational support strengthened the effect of daily positive affect on daily partnership satisfaction such that the effect of daily positive affect on daily partnership satisfaction was stronger under high perceived organisational support than under low perceived organisational support (γ = .04; p < .05)<br>• perceived organisational support weakened the effect of daily negative affect on daily partnership satisfaction such that the effect of daily negative affect on daily partnership satisfaction was weaker under high perceived

Author(s), Year of Publication, Study Location(s)	Publication Type, Methodological Approach, Study Design(s)	Sample Size and Population	Study Aim(s)	Main measurements	Important Results
					organisational support than under low perceived organisational support (γ = .04; p < .05)
Brown, Duck, and Jimmieson (2014), Australia	Published article, quantitative, cross-sectional survey	215 university employees	• to provide a clearer explanation of how normative pressure to respond may contribute directly to employee strain and moderate the effects of the e-mail stressors on such outcomes<br>• to study the effects of potential e-mail stressors on emotional exhaustion as mediated and moderated by person and situation variables	Independent Variables:<br>• appraisals of email overload and email uncertainty<br>• normative response pressure<br>• high email quantity<br>• poor email quality<br>Moderators:<br>• normative response pressure<br>Mediators:<br>• appraisals of email overload and email uncertainty<br>Outcomes:<br>• emotional exhaustion<br>• appraisals of email overload and email uncertainty	• handling more email (β = .14; p < .05) and more ambiguous emails (β = .15; p < .05) was significantly associated with higher emotional exhaustion, but there was no significant unique effect of email emotionality and emotional exhaustion (β = .08; n.s.)<br>• higher perceived pressure to respond promptly to emails was significantly associated with higher emotional exhaustion (β = .19; p < .01)<br>• normative response pressure did not add significantly to the explanation of appraised email overload (β = .11; n.s.)<br>• there was a significant email ambiguity × normative response pressure interaction (β = .13; p < .05)<br>• the positive effect of email ambiguity on emotional exhaustion was significant under high normative response pressure, (b = .54; $t(209)$ = 3.03; p < .001), but not under low normative response pressure, (b = .01; $t(209)$ = .081; p = .936), the block of interactions did not contribute significant variance

Author(s), Year of Publication, Study Location(s)	Publication Type, Methodological Approach, Study Design(s)	Sample Size and Population	Study Aim(s)	Main measurements	Important Results
					• the inclusion of the stress appraisals added significantly to prediction of emotional exhaustion, with email overload (β = .23; $p < .01$) but not email uncertainty (β = -.03; n.s.) as a significant unique predictor<br>• the relationship between email quantity and emotional exhaustion was fully mediated by appraised email overload (effect size estimate = .749; 95% CI: .002 - .009)<br>• the relationship between email ambiguity and emotional exhaustion was fully mediated by email overload (effect size estimate = .372; 95% CI: .026 - .220)<br>• there was no significant increment in the explanation of email uncertainty appraisal with the inclusion of the Email Stressor × Normative response pressure interactions (β = -.02; n.s.)
Califf and Brooks (2020), USA	Published article, quantitative, cross-sectional survey	416 K-12 teachers	• to investigate what teachers as a community can do to help mitigate the negative impacts of the five techno-stressors	Independent Variables:<br>• literacy facilitation<br>• techno-overload<br>• techno-invasion<br>• techno-complexity<br>• techno-insecurity<br>• techno-uncertainty<br>Outcomes:	• techno-complexity was not significantly positively related to burnout (β = .04; n.s.)<br>• techno-insecurity was positively related to burnout (β = .25; $p < .001$)<br>• techno-invasion was positively related to burnout (β = .22; $p < .001$)

Author(s), Year of Publication, Study Location(s)	Publication Type, Methodological Approach, Study Design(s)	Sample Size and Population	Study Aim(s)	Main measurements	Important Results
			• to recognize and investigate the role that technology-related stressors play in burnout and turnover intention experienced by K-12 teachers, as well as how to curb the effect of the techno-stressors and burnout	• techno-overload<br>• techno-invasion<br>• techno-complexity<br>• techno-insecurity<br>• techno-uncertainty<br>• burnout	• techno-overload was positively related to burnout (β = .16; $p < .01$)<br>• techno-uncertainty was not significantly positively related to burnout (β = .02; n.s.)<br>• literacy facilitation was negatively related to techno-complexity (β = .23; $p < .001$)<br>• literacy facilitation was negatively related to techno-insecurity (β = .28; $p < .001$)<br>• literacy facilitation was negatively related to techno-invasion (β = .30; $p < .001$)<br>• literacy facilitation was negatively related to techno-overload (β = .24; $p < .001$)<br>• literacy facilitation was not significantly negatively related to techno-uncertainty (β = .04; n.s.)<br>• literacy facilitation was negatively related to burnout (β = .10; $p < .05$)<br>• burnout was positively related to turnover intention (β = .36; $p < .001$)
Cho, Kim, Chin, & Ahmad (2020), USA	Published article, quantitative, longitudinal survey	98 employees using ICT	• to test an integrative model of spillover effects and role conflict	Independent Variables:<br>• ICT demands during work (response expectations,	• response expectations (*B* = .01; n.s.) and poor communication (*B* = .08; n.s.) were not significantly positively related to work-family conflict in the evening

Author(s), Year of Publication, Study Location(s)	Publication Type, Methodological Approach, Study Design(s)	Sample Size and Population	Study Aim(s)	Main measurements	Important Results
			for diverse types of work-related ICT demands	poor communication, hassles)<br>• ICT demands after work (extended availability)<br>• end-of-work negative affect<br>Moderator:<br>• boundary control<br>Mediator:<br>• end-of-work negative affect<br>Outcomes:<br>• end-of-work negative affect<br>• work-family conflict	• technology-related hassles were positively related to work-family conflict in the evening (B = .19; p < .05)<br>• end-of-work negative affect mediated the relationships between response expectations (c = .045; 95% CI: .014 - .082) and poor communication (c = .131; 95% CI: .008 - .270), but not the relationship between technology-related hassles (c = .02; 95% CI: -.02 - .06) with work-family conflict in the evening<br>• extended availability was positively related to work-family conflict in the evening (γ = .70; p < .001)<br>• boundary control moderated (buffered) the relationship between extended availability and work-family conflict in the evening (γ = −.53; p = .014)
Choi (2020), South Korea	Published article, quantitative, cross-sectional survey	305 workers	• to identify the factors of the psychological attitude related to social media based on empirical analysis	Independent Variables:<br>• social media techno-overload<br>• social media techno-invasion<br>• social media techno-uncertainty<br>Moderators:<br>• perceived organisational support	• female employees were more likely to feel psychological distress than male employees<br>• social media techno- invasion (β = .131; p < .01) and social media techno-uncertainty (β = .088; p < .01) were positively associated with psychological distress

Author(s), Year of Publication, Study Location(s)	Publication Type, Methodological Approach, Study Design(s)	Sample Size and Population	Study Aim(s)	Main measurements	Important Results
				Outcomes:<br>• psychological distress	• social media techno-overload was shown to have no significance with psychological distress<br>• perceived organisational support had a moderating effect on the relationship (β = -.104; $p < .05$) between social media techno-uncertainty and psychological distress<br>• perceived organisational support had no effect on the relation between social media techno-invasion and psychological distress<br>• when employees had higher perceived organisational support in workplace, uncertainty they felt from social media use had weaker impact on their psychological distress
Christ-Brendemühl and Schaarschmidt (2020), Germany	Published article, quantitative, cross-sectional survey (dyadic design)[1]	147 front line service employees (and 373 customers)[1]	• investigating to which extent technological demands arouse front line service employees' technostress and how the latter affects customer satisfaction and delight with the	Independent Variables:<br>• role ambiguity<br>• optimism towards technology<br>• customer satisfaction<br>• role overload<br>• technostress (strain)<br>Moderator:<br>• optimism towards technology<br>Outcomes:	• technostress (strain) influenced both customer satisfaction (β = −.242; $p < .01$) and delight with the front line service employees (β = −.243; $p < .01$) negatively, accounting for 6% of the variance explained of each customer satisfaction and delight with the front line service employees<br>• role overload (β = .228; $p < .05$) and role ambiguity (β = .299; $p < .01$) positively

Author(s), Year of Publication, Study Location(s)	Publication Type, Methodological Approach, Study Design(s)	Sample Size and Population	Study Aim(s)	Main measurements	Important Results
			front line service employees as well as electronic word-of-mouth<br>• to apply the JD-R Model to service encounters in which the personal interaction between front line service employees and customers is augmented by technology	• customer delight<br>• customer satisfaction<br>• technostress (strain)	influenced front line service employees' level of technostress (strain)<br>• optimism towards technology had a direct negative effect on technostress ($\beta = -.300$; $p < .05$), accounting for 23% of the variance explained in technostress (strain)<br>• optimism towards technology moderated the relationship between technology-induced role ambiguity and technostress ($b = -.15$; $p < .05$), but not the relationship between role overload and technostress (strain) (n.s.)
D'Arcy, Hearth, and Shoss (2014), USA	Published article, quantitative, cross-sectional survey	539 employed, computer-using professionals	• to address the gap in the information systems literature of a systematic, theory-driven investigation of the potential adverse effects of organizational information security on user behaviour	Independent Variables:<br>• security-related technostress<br>Mediators:<br>• moral disengagement from information security policy violations<br>Outcomes:<br>• information security policy violation intention<br>• moral disengagement from information security policy violations	• security-related technostress was positively associated with moral disengagement from information security policy violations (model explained approximately 46% of the variance in information security policy violation intention and approximately 44% of the variance in moral disengagement<br>• security-related technostress had a significant positive relationship with moral disengagement ($\beta = .358$; $p < .001$)

Author(s), Year of Publication, Study Location(s)	Publication Type, Methodological Approach, Study Design(s)	Sample Size and Population	Study Aim(s)	Main measurements	Important Results
					• moral disengagement played a mediating role between security-related technostress and information security policy violation intention ($z = 7.98$; $p < .001$)
Day, Paquet, Scott, and Hambley (2012), Canada	Published article, quantitative, cross-sectional survey	258 employees (random sample of occupations)	• to address the lack of validated measures designed to assess the ICT factors that may impact employee well-being by developing and validating a measure of ICT demands and supports	Independent Variables:<br>• hassles<br>• learning expectations<br>• ICT demands<br>• ICT organisational support<br>Moderators:<br>• ICT organisational support<br>Outcomes:<br>• perceived stress<br>• strain<br>• burnout	• ICT demand subscales accounted for a significant increase in variance in strain ($R^2 = .07$; $p < .05$) and perceived stress ($R^2 = .23$, $p < .001$)<br>• ICT personal assistance and resource support were negatively related to increased strain and burnout (r_s ranged from $r = -.14$; $p < .05$ to $r = -.29$; $p < .001$)<br>• when employees experienced low levels of learning expectations, strain was relatively low for all employees, regardless of the degree of ICT resource support they received<br>• there was a significant positive relationship between learning expectations and strain for employees who have a low degree of ICT resource support at work, indicating that strain was significantly higher for employees who experienced demands to learn new technologies and when they had low levels of ICT resource support<br>• professional efficacy was significantly lower when employees experience high

Author(s), Year of Publication, Study Location(s)	Publication Type, Methodological Approach, Study Design(s)	Sample Size and Population	Study Aim(s)	Main measurements	Important Results
					learning expectations and low levels of support<br>• ICT hassles accounted for a significant increase in explained variance in all of the outcomes (R^2_{change} ranged from R^2_{change} = .05 to R^2_{change} = .10; $p < .001$), with the exception of professional efficacy (R^2_{change} = .01; p = n.s.)<br>• the support variables accounted for increased variance in all of the outcomes (R^2_{change} ranged from .03; $p < .05$ to .05, $p < .001$), with the exception of stress (R^2_{change} = .01; p = n.s.) and efficacy (R^2_{change} = .02; p = n.s.)<br>• in the fourth step, the interaction terms only accounted for a significant increase in explained variance in strain (R^2_{change} = .03; $p < .01$), although they accounted for ≥ 1% of the variance in all outcomes<br>• personal assistance moderated the relationships between hassles and strain ($\beta = -.19$; $p < .01$) and between hassles and cynicism ($\beta = -.14$; $p < .05$)<br>• strain was moderate at low levels of assistance, regardless of the amount of hassles experienced<br>• there was a negative relationship between hassles and strain at high levels

Author(s), Year of Publication, Study Location(s)	Publication Type, Methodological Approach, Study Design(s)	Sample Size and Population	Study Aim(s)	Main measurements	Important Results
					of personal assistance, such that strain was lowest when employees reported high hassles and high assistance<br>• ICT resource support moderated the relationship between hassles and strain (β = -.15; $p < .05$)<br>• hassles were unrelated to strain at low levels of resource support (i.e. strain was relatively moderate for employees who received little ICT resource support, regardless of the degree of hassles they experienced)<br>• for employees who received a high degree of resource support, there was a significant relationship between hassles and strain, such that strain was significantly lower when they experience few ICT hassles
Delpechitre, Black, and Farrish (2019), USA	Published article, quantitative, cross-sectional survey	218 salespeople	• to understand the impact of technology overload on salespeople's role stress, technology self-efficacy, the amount of effort they put forth to integrate	Independent Variables:<br>• technology overload (system feature overload, information overload and communication overload)<br>Moderators:<br>• technology self-efficacy<br>Outcomes:<br>• administrative performance	• information overload and communication overload had a negative linear relationship with salesperson's administration performance (information overload: β = -.147; $p < .05$; communication overload: β = -.088; $p < .05$)<br>• system feature, information and communication overload had a negative relationship with salesperson's outcome

Author(s), Year of Publication, Study Location(s)	Publication Type, Methodological Approach, Study Design(s)	Sample Size and Population	Study Aim(s)	Main measurements	Important Results
			technology into their work routines and its impact on salesperson's job performance	• outcome performance<br>• effort in using technology<br>• role ambiguity<br>• role conflict	performance (system feature overload: β = -.121; *p* < .05; information overload: β = -.298; *p* < .001; communication overload: β = -.148; *p* < .05)<br>• salesperson's technology self-efficacy strengthened the relationship technology overload (system feature overload, information overload and communication overload) had on salesperson's effort to use the technology<br>• salesperson's technology self-efficacy weakened the negative relationship between technology overload and salesperson's administrative performance and outcome performance<br>• findings did not provide evidence to support the moderating effect technology efficacy has on the relationships between technology overload and salesperson's role stress<br>• technology overload (system feature overload, information overload and communication overload) was found to have a significant positive linear relationship with role ambiguity (system feature overload: β = .423; *p* < .001; information overload: β= .199; *p* < .001; communication overload: β = .220; *p* <

Author(s), Year of Publication, Study Location(s)	Publication Type, Methodological Approach, Study Design(s)	Sample Size and Population	Study Aim(s)	Main measurements	Important Results
					.001) and role conflict (system feature overload: β = .485; $p < .001$; information overload: β = .285; $p < .001$; communication overload: β = .399; $p < .001$)
Fieseler, Grubenmann, Meckel, and Müller (2014), different European countries[2]	Conference paper, quantitative, cross-sectional survey	491 salespeople	• to extend research into the role of management to balance the negative consequences of technostress	Independent Variables:<br>• work exhaustion<br>• leadership<br>• supervisor influence on ICT usage<br>• technostress (ICT strain)<br>• techno-overload<br>• techno-invasion<br>• techno-complexity<br>• techno-insecurity<br>• techno-uncertainty<br>Moderators:<br>• leadership<br>Outcomes:<br>• job satisfaction<br>• work exhaustion<br>• technostress (ICT strain)	• job satisfaction was mostly affected by work exhaustion (β = -.600; $p < .01$) and leadership (β = .293; $p < .01$)<br>• techno-overload and techno-complexity affected work exhaustion and job satisfaction indirectly<br>• work exhaustion was mostly influenced by the perceived technostress (ICT strain) (β = .722)<br>• leadership also showed a significant impact on work exhaustion (β = -.157)<br>• in conjunction with leadership, technostress (ICT strain) accounted for approximately 57% of the observed variance in work exhaustion<br>• no significant impact could be found for the influence of the supervisor on work exhaustion (β = .036; n.s.) or technostress (ICT strain) (β = -.018; n.s.)<br>• techno-overload (β = .577; $p < .01$) and techno-complexity (β = .342; $p < .01$) did impact technostress (ICT strain), but there was no significant direct influence

Author(s), Year of Publication, Study Location(s)	Publication Type, Methodological Approach, Study Design(s)	Sample Size and Population	Study Aim(s)	Main measurements	Important Results
					from techno-invasion (β = .064; n.s.) and techno-uncertainty (β = -.076; n.s.) on technostress (ICT strain)<br>• techno-overload and techno-complexity accounted for approximately 68% of the observed variance in perceived technostress (ICT strain)
Fischer and Riedl (2020), Germany	Published article, quantitative, cross-sectional survey	1,115 employees	• pursuing the idea that an organisational climate that encourages and supports the implementation of technological innovations and therefore entails organisational disruption can also have negative side effects	Independent Variables:<br>• perceived unreliability (of ICT)<br>• organisational climate of innovation<br>• perceived uncertainty<br>Outcomes:<br>• perceived uncertainty<br>• perceived unreliability (of ICT)<br>• user satisfaction<br>• job satisfaction	• organisational climate of innovation was positively related to perceived uncertainty (β = .228; $p < .001$; C. R. = 4.193; $f^2 = .055$)<br>• organisational climate of innovation was negatively related to perceived unreliability (β = −.231; $p < .001$; C. R. = −4.607; $f^2 = .085$)<br>• perceived uncertainty was not significantly negatively related to user satisfaction (β = .009; $p = .876$; C. R. = .156)<br>• perceived uncertainty was not significantly negatively related to job satisfaction (β = .037; $p = .499$; C. R. = .676)<br>• perceived unreliability was negatively related to user satisfaction (β = −.303; $p < .001$; C. R. = −5.720; $f^2 = .064$)<br>• perceived unreliability was not significantly negatively related to job satisfaction (β = −.087; $p = .104$; C. R. = −1.625)

Author(s), Year of Publication, Study Location(s)	Publication Type, Methodological Approach, Study Design(s)	Sample Size and Population	Study Aim(s)	Main measurements	Important Results
					• organisational climate of innovation was positively related to user satisfaction (β = .422; $p < .001$; C. R. = 8.117; $f^2 = .189$)<br>• organisational climate of innovation was positively related to job satisfaction (β = .515; $p < .001$; C. R. = 7.831; $f^2 = .336$)<br>• perceived uncertainty was positively related to perceived unreliability (β = .537; $p < .001$; C. R. = 9.384; $f^2 = .389$)<br>• user satisfaction was positively related to job satisfaction (β = .277; $p < .001$; C. R. = 5.192; $f^2 = .007$)
Fuglseth and Sørebø (2014), Norway	Published article, quantitative, cross-sectional survey	216 employees of local government administration	• to investigate direct and moderating effects of technostress variables on employee use of ICT at work, particularly in order to identify specific measures to cope with negative effects of technostress, both to support the employees and to improve	Independent Variables:<br>• involvement facilitation<br>• technical support provision<br>• literacy facilitation<br>• techno-overload<br>• techno-invasion<br>• techno-complexity<br>• techno-insecurity<br>• techno-uncertainty<br>Moderators:<br>• technical support provision<br>• involvement facilitation<br>• literacy facilitation<br>Mediators:	• employee perceptions of the existence of technostress creators in their organisational environment were negatively associated with their level of satisfaction with ICT use (β = -.42; $p < .001$)<br>• employee perceptions of the existence of technostress inhibitors in their organisational environments were positively associated with their level of satisfaction with ICT use (β = .18; $p < .05$)<br>• employee perceptions of the existence of technostress inhibitors in their organisational environments were not significantly positively associated with

Author(s), Year of Publication, Study Location(s)	Publication Type, Methodological Approach, Study Design(s)	Sample Size and Population	Study Aim(s)	Main measurements	Important Results
			organisational performance<br>• to demonstrate that technostress creators and inhibitors as they have been measured in previous research should be treated as formative variables	• employee satisfaction with ICT use<br>Outcomes:<br>• satisfaction with ICT use<br>• intention to extend ICT use	their level of intention to extend their use of ICT (β = .03; n.s.)<br>• employee perceptions of the existence of technostress inhibitors in their organisational environments did not moderate the relationship between technostress creators and satisfaction with the use of ICT<br>• technostress creators mediated the relationship between employee satisfaction with the use of ICT and employee intentions to extend the use of ICT (z = -.37; p < .001)
Gaudioso, Turel, and Galimberti (2017)	Published article, quantitative, cross-sectional survey	242 employees	• to explain how techno-stressors, such as techno-invasion and techno-overload, translate through strain facets and coping strategy choices into negative workplace outcomes, such as work exhaustion	Independent Variables:<br>• maladaptive coping strategies<br>• adaptive coping strategies<br>• job distress<br>• work-family conflict<br>• work exhaustion<br>• techno-invasion<br>• techno-overload<br>Mediators:<br>• strain<br>• maladaptive coping strategies<br>• adaptive coping strategies<br>Outcomes:	• techno-invasion levels are positively associated with work-family conflict (β = .66; p < .01)<br>• techno-overload levels were positively associated with job distress (β = .51; p < .01)<br>• adaptive (problem-focused) coping strategies related to work technologies were negatively associated with work exhaustion levels (β = -.63; p < .05)<br>• the effects of techno-overload (95% CI: .154 - .338; p < .004) and techno-invasion (95% CI: .128 - .356; p < .012 for the indirect effect of techno-invasion) on

Author(s), Year of Publication, Study Location(s)	Publication Type, Methodological Approach, Study Design(s)	Sample Size and Population	Study Aim(s)	Main measurements	Important Results
				• job distress<br>• work exhaustion<br>• work-family conflict<br>• maladaptive coping strategies<br>• adaptive coping strategies	work exhaustion were mediated through strain facets and coping strategies<br>• maladaptive strategies exerted stronger positive (promotion) effect on work exhaustion (β = 1.11; $p < .01$), compared with the negative (prevention) effect of adaptive coping strategies (β = -.63; $p < .05$), while both families of strategies influenced work exhaustion, maladaptive strategies had significantly stronger influence on this aversive outcome<br>• work-family conflict was positively associated with adaptive (β = .16; $p < .05$) and maladaptive coping strategies (β = .40; $p < .01$)<br>• perceived distress on the job was positively associated with adaptive (β = .21; $p < .01$) and maladaptive coping strategies (β = .50; $p < .01$)
Gimpel, Lanzl, Regal, Urbach, Wischnewski, et al. (2019), Germany	Research report, quantitative, cross-sectional survey	5,005 (knowledge) workers	• to analyse the changes in the stress and strain profile due to digitalisation in Germany<br>• to develop specific prevention	Independent Variables:<br>• digital stressors (performance monitoring, transparency/presenteeism of ICT, unreliability, interruption, techno-overload, techno-uncertainty, unavailability,	• job demands included performance monitoring, lacking a sense of achievement, perceived unreliability of ICT, interruptions, unavailability of required technology or media, culture of innovation, social conflicts, techno-overload, techno-invasion, role ambiguity, invasion of privacy, techno-

Author(s), Year of Publication, Study Location(s)	Publication Type, Methodological Approach, Study Design(s)	Sample Size and Population	Study Aim(s)	Main measurements	Important Results
			measures to use the potential of modern technologies and media for a humane work design and to avoid adverse stresses and strains	role ambiguity, techno-invasion, techno-complexity, job insecurity, lacking sense of achievement)<br>Outcomes:<br>• turnover intention<br>• intention to quit the job<br>• productivity<br>• job satisfaction<br>• work ability<br>• neurological-sensory diseases<br>• diseases of the digestive system<br>• cardiovascular diseases<br>• musculoskeletal disorders<br>• work exhaustion<br>• cognitive irritation<br>• emotional irritation	complexity, techno-insecurity, techno-uncertainty and an autonomous choice of technologies<br>• environmental resources included a bureaucratic organisational culture, increased scope for action and good relationship with supervisors<br>• personal resources included confidence in dealing with digital technologies and media<br>• coping strategies included learning to live with the situation, looking at the bright side of things and taking them with humour on the emotion-focused side as well as coming up with plans, taking active actions to improve the situation and proactively asking friends or family for support on the problem-focused side<br>• technostress was associated with health-related consequences (greater (work) exhaustion, greater cognitive irritation, greater emotional irritation, ill health, such as increased musculoskeletal disorders, diseases of the digestive system, neurological-sensory disorders, cardiovascular diseases)

Author(s), Year of Publication, Study Location(s)	Publication Type, Methodological Approach, Study Design(s)	Sample Size and Population	Study Aim(s)	Main measurements	Important Results
					• technostress was associated with work-related consequences (decreased work ability, reduced productivity, lower job satisfaction, higher turnover intention, higher intention to quit the job)<br>• perceived technostress was rated the lowest among employees in small and medium-sized enterprises (1.33 compared to an average of 1.48 in larger enterprises on a scale from 0 = "very low" to 4 = "very high")<br>• technostress was perceived the highest in the ICT industry (1.68 on a scale from 0 = "very low" to 4 = "very high") and lowest in the public and private service sector (1.29 on a scale from 0 = "very low" to 4 = "very high")<br>• the degree of digitalisation among German federal states was highest in Saarland (index of 15.73) and lowest in Thuringia (index of 11.14)<br>• the degree of technostress among German federal states was highest in Baden-Wuerttemberg (1.46 on a scale from 0 = "very low" to 4 = "very high") and lowest in Bremen (1.28 on a scale from 0 = "very low" to 4 = "very high")

Author(s), Year of Publication, Study Location(s)	Publication Type, Methodological Approach, Study Design(s)	Sample Size and Population	Study Aim(s)	Main measurements	Important Results
					• the degree of digitalisation and the intensity of use of digital technologies and media had an amplifying influence on the reported techno-stressors, i.e. employees who do not work with ICT very often perceived the higher technostress
Goetz and Boehm (2020), Germany[2]	Published article, quantitative, cross-sectional survey	8,019 employees	• investigate to which extent the negative effect of technological insecurity on perceived health depends upon the availability of non-technology-related resources in the workplace<br>• To jointly address research gaps, by integrating the Conservation of Resources theory with Information Systems-related work on technostress	Independent Variables:<br>• technological insecurity<br>Moderators:<br>• friendship opportunities at work<br>• organisational support for strengths use<br>Outcomes:<br>• employees' perceived general health	• technological insecurity was negatively related to employees' perceived general health ($B = -.27$; $p < .001$; $F = 81.47$; $df = 3$; $p < .01$)<br>• organisational support for strengths use moderated the relationship between technological insecurity and general health such that the negative relationship between the two is weaker when organisational support for strengths use is higher ($B = .12$; $p < .01$; $F = 9.94$; $df = 1$; $p < .01$)<br>• friendship opportunities moderated the relationship between technological insecurity and general health such that the negative relationship between the two is weaker when friendship opportunities are higher ($B = .18$; $p < .01$; $F = 9.94$; $df = 1$; $p < .01$)<br>• the combination of both job resources, strengths use support and friendship

Author(s), Year of Publication, Study Location(s)	Publication Type, Methodological Approach, Study Design(s)	Sample Size and Population	Study Aim(s)	Main measurements	Important Results
					opportunities per se, in absence of the stressful job condition technological insecurity, did not have a buffering effect on general health beyond the simple direct effects caused by the individual resources (*B* = .05; *p* > .05; *F* = 1.45; *df* = 1; *p* > .05)<br>• there was a three-way interaction among technological insecurity, organisational support for strengths use and friendship opportunities in explaining general health n (*B* = .14; *p* < .01; *F* = 5.42; *df* = 4; *p* < .01)<br>• the negative relationship between technological insecurity and general health was stronger when both organisational support for strengths use (I) and friendship opportunities (II) were low or when organisational support for strengths was low and friendship opportunities were high, or vice versa (I↓II↓: *B* = .42; *SE* = .06; *p* < .01; I↑II↓: *B* = .37; *SE* = .09; *p* < .01; I↓II↑: *B* = .40; *SE* = .10; *p* < .01). The only insignificant moderating condition was when both, organisational support for strengths use and friendship opportunities were high (I↑II↑: *B* = .06; *SE* = .06; *p* > .05).

Author(s), Year of Publication, Study Location(s)	Publication Type, Methodological Approach, Study Design(s)	Sample Size and Population	Study Aim(s)	Main measurements	Important Results
Harris, Lambert, and Harris (2013), USA	Published article, quantitative, cross-sectional survey	219 employees	• to examine an interpersonal stressor (abusive supervision) and a contextual stressor (technology work overload) for technology end users and their relationships with job strains and perceived organisational support<br>• to investigate how Human Resource Management effectiveness interacts with the relationships between either abuse and technology work overload and job strains and perceived organisational support	Independent Variables:<br>• technology-related overload<br>Moderators:<br>• Human Resource Management effectiveness<br>Outcomes:<br>• perceived organisational support<br>• job strain	• technology work overload was positively related to job strain (β = .21; $p < .01$), but not to perceived organisational support (β = .01; n.s.)<br>• Human Resource Management effectiveness did not moderate the relationship between technology-related overload and perceived organisational support such that the negative relationship is less strong when Human Resource Management effectiveness is high (β = .00; n.s.)<br>• the technology work overload × Human Resource Management effectiveness interaction was significantly related to only job strain (β = -.13; $p < .05$), i.e. Human Resource Management effectiveness was able to buffer the negative impacts of technology overload on job strain, but when human resource management effectiveness was lacking, technology overload was more strongly associated with job strain

Author(s), Year of Publication, Study Location(s)	Publication Type, Methodological Approach, Study Design(s)	Sample Size and Population	Study Aim(s)	Main measurements	Important Results
			• to employ the conservation of resources theory framework as our theoretical perspective, which to this point has not been utilized as a lens for examining technology work overload		
Harris, Harris, Carlson, and Carlson (2015), USA	Published article, quantitative, cross-sectional survey	219 employees	• to investigate how the three different types of technology overload are associated with work-family conflict for technology end-users<br>• to examine Leader-Member exchange as a potential moderator of the technology	Independent Variables:<br>• information overload<br>• communication overload<br>• system feature overload<br>Moderators:<br>• leader-member exchange<br>Outcomes:<br>• work-family conflict	• information overload (β = .13; p < .05), communication overload (β = .12; p < .05), and system feature overload (β = .27; p < .01) were positively related to work-family conflict, all together explaining 24% of the variance in work-family conflict<br>• leader–member exchange quality did not moderate the positive relationship between information overload and work-family conflict, such that the relationship was weakest when Leader–member exchange quality is highest; the opposite effect was found (t = 3.79; p < .01)<br>• leader–member exchange quality moderated the positive relationship

Author(s), Year of Publication, Study Location(s)	Publication Type, Methodological Approach, Study Design(s)	Sample Size and Population	Study Aim(s)	Main measurements	Important Results
			overload-work-family conflict relationship		between communication overload and work-family conflict, such that the relationship was weakest when leader–member exchange quality is highest (t = 2.90; $p < .05$)<br>• leader–member exchange quality moderated the positive relationship between system feature overload and work-family conflict, such that the relationship was weakest when leader–member exchange quality is highest (t = 3.99; $p < .01$)
Hauk, Göritz, and Krumm (2019), Germany, Austria, and Siwtzerland[3]	Published article, quantitative, longitudinal survey	1,216 employees	• to evaluate how workers across the age-span cope with technostress in a digitalised work environment	Independent Variables:<br>• age<br>Mediators:<br>• techno-overload<br>• techno-invasion<br>• techno-complexity<br>• techno-insecurity<br>• techno-uncertainty<br>• seeking instrumental support<br>• behavioural disengagement<br>• active coping<br>Outcomes:<br>• active coping	• age was not significantly positively correlated with techno-stressors at t1, t2, or t3 (r_{T1} = -.05; r_{T2} = -.10; r_{T3} = -.06)<br>• chronological age and the use of active coping were not positively but negatively related to active coping at t1 (r_{T1} = -.10) and not related to active coping at t2 and t3 (r_{T2} = -.02; r_{T3} = -.02)<br>• chronological age and the use of social coping were not positively but negatively related to social coping at t1 and t2 (r_{T1} = -.13; r_{T2} = -.07), and unrelated at t3 (r_{T3} = -.05)<br>• chronological age and the use of dysfunctional coping strategies, such as behavioural disengagement, were

Author(s), Year of Publication, Study Location(s)	Publication Type, Methodological Approach, Study Design(s)	Sample Size and Population	Study Aim(s)	Main measurements	Important Results
				• seeking instrumental support<br>• behavioural disengagement<br>• techno-overload<br>• techno-invasion<br>• techno-complexity<br>• techno-insecurity<br>• techno-uncertainty<br>• techno-strain	negatively related at all measurement points (r_{T1} = -.10; r_{T2} = -.14; r_{T3} = -.14)<br>• age was significantly negatively correlated with technology-related strain at all three measurement points (r_{T1} = -.08; r_{T2} = -.14; r_{T3} = -.13)<br>• there was no significant indirect effect of age on technology-related strain via social coping (β = -.00; p = .394), techno-stressors (β = -.03; p = .222), or both mediators (β = .00; p = .447)<br>• there was a significant indirect effect of age on technology-related strain via behavioural disengagement (β = -.04; p < .001), but not via techno-stressors (β = -.02; p = .226), or via both mediators (β = -.01; p = .245)<br>• age effects were driven by negative direct effects of age on behavioural disengagement (β = -.11; p < .001)<br>• the negative association between age and behavioural disengagement buffered from techno-stressors to increased behavioural disengagement (β = .78; p < .001), and from behavioural disengagement to increasing technology-related strain (β = .33; p < .001)

Author(s), Year of Publication, Study Location(s)	Publication Type, Methodological Approach, Study Design(s)	Sample Size and Population	Study Aim(s)	Main measurements	Important Results
					• the positive relationship between techno-stressors and technology-related strain (β = .30; *p* < .001) was partly mediated by active coping (β = .01; *p* < .05), social coping (β = .01; *p* < .05), and behavioural disengagement (β = .03; *p* < .001)
Hung, Chang, and Lin (2011), Taiwan	Conference paper, quantitative, cross-sectional survey	622 employees	• to identify the ubiquitous technostress creators<br>• to test how they impact on job stress and individual productivity	Independent Variables:<br>• organisational rewards<br>• stress management training<br>• job control<br>• techno-accessibility<br>• techno-dependency<br>• techno-invasion<br>• techno-overload<br>Moderators:<br>• stress management training<br>• job control<br>• organisational rewards<br>Outcomes:<br>• individual productivity<br>• job stress	• ubiquitous technostress creators had a positive effect on job stress (β = .60; *p* < .001)<br>• ubiquitous technostress creators had a positive effect on individual productivity (β = .26; *p* < .001)<br>• stress inhibitors did not have a negative effect on job stress (β = .034; *t* = -.976; *p* = .329)<br>• stress inhibitors had a positive effect on individual productivity (β = .71; *p* < .001)<br>• stress inhibitors did not have a negative moderating effect on the relationship between the ubiquitous technostress and job stress (β = 1.358; *p* = .108)<br>• job stress had a negative effect on productivity (β = -.24; *p* < .001)
Hung, Chen, and Lin (2015), Taiwan	Published article, quantitative, cross-sectional survey	601 work-related mobile phone users	• to study technostress at all user experience levels, which extends the	Independent Variables:<br>• confronting situations head-on<br>• transforming situations into opportunities	• communication overload had a negative relationship with productivity (β = -.299; *p* < .001)

Author(s), Year of Publication, Study Location(s)	Publication Type, Methodological Approach, Study Design(s)	Sample Size and Population	Study Aim(s)	Main measurements	Important Results
			existing research that has focused primarily on technological unfamiliarity as the main source of technostress<br>• to uncover insights on the effect of the role of the proactive personality on the relationship between technostress and productivity	• accessibility<br>• communication overload<br>• techno-overload<br>Moderators:<br>• confronting situations head-on<br>• transforming situations into opportunities<br>Outcomes:<br>• communication overload<br>• techno-overload<br>• individual productivity	• techno-overload had a positive relationship with productivity (β = .317; $p < .001$)<br>• accessibility had a positive relationship with productivity (β = -.125; $p < .001$)<br>• accessibility had a positive relationship with communication overload (β = .486; $p < .001$)<br>• accessibility had a positive relationship with techno-overload (β = .566; $p < .001$)<br>• transformation of situations had a positive relationship with productivity (β = .188; $p < .001$)<br>• confrontation of situations had a positive relationship with productivity (β = .178; $p < .001$)<br>• transformation of situations did not moderate the negative relationship with communication overload on productivity (β = .063; n.s.)<br>• transformation of situations did not moderate the negative relationship with techno-overload on productivity (β = -.019; n.s.)<br>• confrontation of situations moderated the negative relationship with communication overload on productivity (β = -.118; $p < .05$)

Author(s), Year of Publication, Study Location(s)	Publication Type, Methodological Approach, Study Design(s)	Sample Size and Population	Study Aim(s)	Main measurements	Important Results
					• confrontation of situations did not moderate the negative relationship with techno-overload on productivity (β = -.057; n.s.)
Hwang and Cha (2018), South Korea	Published article, quantitative, cross-sectional survey	346 employees in information security departments	• to introduce the concept of technostress and role stress to understand the circumstances and experiences of employees in an organization in relation to information security<br>• to test how employees' experiences relate to technostress creators and how resulting role stress affects their compliance intention through organisational commitment	Independent Variables:<br>• security-related role stress<br>• security-related techno-stress<br>• organisational commitment<br>Moderators:<br>• promotion focus (regulatory focus)<br>• prevention focus (regulatory focus)<br>Mediators:<br>• security-related role stress<br>Outcomes:<br>• security-related role stress<br>• compliance intention<br>• organisational commitment	• security-related technostress creators negatively affected organisational commitment (β = -.423; $p < .01$)<br>• organisational commitment had a positive influence on compliance intention (β = .490; $p < .01$)<br>• security-related technostress creators significantly increased security-related role stress (β = .233; $p < .05$)<br>• security-related role stress decreased the organisational commitment (β = -.490; $p < .01$)<br>• the moderation effect regarding the promotion focus was statistically significant (β = -.168; $p < .05$), whereas the moderation effect regarding the prevention focus was not (β = -.126; $p > .05$), thus employees with a strong promotion focus experienced less role stress than employees with a weak promotion focus<br>• security-related technostress creators negatively affected information security

Author(s), Year of Publication, Study Location(s)	Publication Type, Methodological Approach, Study Design(s)	Sample Size and Population	Study Aim(s)	Main measurements	Important Results
			• to explore the regulatory focus (i.e. promotion focus and prevention focus) as a moderating variable determining the strength of the relationship between technostress creators and role stress		compliance through organisational commitment
Ioannou and Papazafeiropoulou (2017), United Kingdom	Conference paper, quantitative, cross-sectional survey	440 employees	• to explore the role of IT mindfulness as a buffer to technostress stressors as well as a mechanism that can mitigate the negative consequences arising from extended ICT usage within organizational settings	Independent Variables: • IT mindfulness • techno-overload • techno-invasion • techno-complexity • techno-insecurity • techno-uncertainty Outcomes: • techno-overload • techno-invasion • techno-complexity • techno-insecurity • techno-uncertainty • end user performance	• IT mindfulness decreased the impact of technostress (i.e. techno-overload, techno-invasion, techno-complexity, techno-insecurity, techno-uncertainty) on individuals ($b = -.541$; $p < .001$) • technostress creators (i.e. techno-overload, techno-invasion, techno-complexity, techno-insecurity, techno-uncertainty) affected an end user's performance while using ICT applications ($b = -.273$; $p = .001$) • a significant negative correlation between technostress creators (i.e. techno-overload, techno-invasion,

Author(s), Year of Publication, Study Location(s)	Publication Type, Methodological Approach, Study Design(s)	Sample Size and Population	Study Aim(s)	Main measurements	Important Results
			• to propose a theoretical model that suggests IT mindfulness as mechanism that can act as a buffer to the exposure of technostress stressors and on the outcome strain, alleviate the adverse effects arising from extended ICT usage within occupational settings and ultimately contribute to employee well-being	• end user satisfaction	techno-complexity, techno-insecurity, techno-uncertainty) and end user satisfaction was found (*b* = -.170; *p* = .027)<br>• a significant positive relationship between IT mindfulness and end user satisfaction was found (*b* = .456; *p* < .001)
Jena (2015), India	Published article, quantitative, cross-sectional survey	216 academicians	• to find out the cause and effect of technostress among Indian academicians<br>• to identify the important	Independent Variables:<br>• involvement facilitation<br>• literacy facilitation<br>• technical support provision<br>• techno-overload<br>• techno-invasion	• technostress inhibitors (involvement facilitation, literacy facilitation, technical support provision) negatively influenced technostress creators (techno-overload, techno-invasion, techno-complexity, techno-insecurity, techno-uncertainty) (β = -.34; *p* < .05)

Author(s), Year of Publication, Study Location(s)	Publication Type, Methodological Approach, Study Design(s)	Sample Size and Population	Study Aim(s)	Main measurements	Important Results
			inhibitor to reduce the impact of technostress in collaborative teaching and learning environment	• techno-complexity<br>• techno-insecurity<br>• techno-uncertainty<br>Outcomes:<br>• techno-overload<br>• techno-invasion<br>• techno-complexity<br>• techno-insecurity<br>• techno-uncertainty<br>• negative affectivity<br>• technology-enabled performance<br>• organisational commitment<br>• job satisfaction	• participants with a higher degree of technostress (techno-overload, techno-invasion, techno-complexity, techno-insecurity, techno-uncertainty) had a greater likelihood of less Technology enabled performance (β = -.34; $p < .05$)<br>• participants with higher values of technostress inhibitors (involvement facilitation, literacy facilitation, technical support provision) had a greater likelihood of more technology enabled performance (β = .32; $p < .05$)<br>• technostress Inhibitors positively affected the organisational commitment (β = .29; $p < .05$)<br>• participants with higher values of technostress inhibitors were more likely to have more job satisfaction (β = .31; $p < .05$)<br>• there was a significant relationship between technostress inhibitor and negative affectivity (β = -.35; $p < .05$)
Kim & Lee (2021), South Korea	Published journal article, Quantitative, cross-sectional survey	620 employees from different industries	• to test the effect of technostress on counter-productivity<br>• to examine the moderating effect	Independent Variables:<br>• techno-overload<br>• techno-invasion<br>• techno-complexity<br>• techno-insecurity<br>• techno-uncertainty	• techno-invasion (β = .318; $p < .001$), techno-complexity (β = .162; $p < .001$), and techno-insecurity (β = .237; $p < .001$) were significantly positively related to counterproductive work behaviour

Author(s), Year of Publication, Study Location(s)	Publication Type, Methodological Approach, Study Design(s)	Sample Size and Population	Study Aim(s)	Main measurements	Important Results
			of self-efficacy and technical support in the relationship between technostress and counter-productivity.	Moderators:<br>• self-efficacy<br>• technical support<br>Outcomes:<br>• counterproductive work behaviour<br>• innovation resistance	• techno-overload (β = .063; *p* = .093) and techno-uncertainty (β = .018; *p* = .632) were not significantly positive related to counterproductive work behaviour<br>• techno-overload (β = .129; *p* = .001), techno-invasion(β = .087; *p* = .031), techno-complexity (β = .099; *p* = .013), and techno-insecurity (β = .140; *p* = .001) were significantly positively related to innovation resistance<br>• techno-uncertainty (β = .005; *p* = .905) was not significantly positively related to innovation resistance<br>• self-efficacy was significantly negatively related to counterproductive work behaviour (β = -.189; *p* < .001) and to innovation resistance (β = -.406; *p* < .001)<br>• self-efficacy significantly moderated (buffered) the relationships between techno-overload (β = -.084; *p* = .006), techno-invasion (β = -.153; *p* < .001) and techno-insecurity (β = -.082; *p* = .036) and counterproductive work-behaviour<br>• the moderating (buffering) effect of self-efficacy between techno-complexity (β = -.034; *p* = .241) and techno-uncertainty (β = -.024; *p* = .538) and counterproductive work behaviour was not significant

Author(s), Year of Publication, Study Location(s)	Publication Type, Methodological Approach, Study Design(s)	Sample Size and Population	Study Aim(s)	Main measurements	Important Results
					• self-efficacy significantly moderated (buffered) the relationship between techno-complexity and innovation resistance (β = -.082; *p* = .005)<br>• the moderating (buffering) effect of self-efficacy between techno-overload (β = -.001; *p* = .984), techno-invasion (β = -.015; *p* = .696), techno-insecurity (β = -.034; *p* = .389), techno-uncertainty (β = -.003; *p* = .943) and innovation resistance was not significant<br>• technical support was significantly negatively associated with counterproductive work behaviour (β = -.340; *p* < .001) and innovation resistance (β = -.229; *p* < .001)<br>• technological support significantly moderated (buffered) the relationship between techno-insecurity and counterproductive work behaviour (β = -.075; *p* = .031), but not between techno-overload (β = -.017; *p* = .567), techno-invasion (β = -.054; *p* = .152), techno-complexity (β = -.049; *p* = .151) or techno-uncertainty (β = -.053; *p* = .155) and counterproductive work behaviour<br>• technological support significantly moderated (buffered) the relationships

Author(s), Year of Publication, Study Location(s)	Publication Type, Methodological Approach, Study Design(s)	Sample Size and Population	Study Aim(s)	Main measurements	Important Results
					between techno-overload (β = -.197; p < .001), techno-invasion (β = -.088; p = .032), techno-uncertainty (β = -.098; p = .015) and innovation resistance, but not between techno-complexity (β = -.002; p = .962) or techno-insecurity (β = -.035; p = .350) and innovation resistance
Koo and Wati (2011), South Korea	(Edited) Book chapter, quantitative, cross-sectional survey	98 employees	• to investigate the influence of innovation culture, self-efficacy, and task complexity on technostress	Independent Variables:<br>• task complexity<br>• self-efficacy<br>• innovation culture<br>Moderator:<br>• literacy facilitation<br>Outcomes:<br>• techno-overload<br>• techno-invasion<br>• techno-complexity<br>• techno-insecurity<br>• techno-uncertainty	• innovation culture negatively influenced technostress level (β = -.164; p < .05)<br>• self-efficacy had no significant effect on technostress (β = -.069; n.s.)<br>• task complexity significantly influenced technostress level (β = .426; p < .001)<br>• literacy facilitation had a moderating (negative i.e. reducing) effect on the relationship between task complexity and technostress (β = -.219; p < .05)
Kwanya, Stilwell, and Underwood (2012), Kenya and South Africa[4]	Conference paper, qualitative semi-structured survey	25 academic librarians	• to unravel the mechanisms academic librarians in Eastern and Southern Africa employ to cope with the consequences of	• seriousness of technostress<br>• causes of technostress<br>• ways of coping with or minimising technostress<br>• technolust	• demands included perceived technostress, techno-overload, techno-complexity, techno-insecurity, techno-uncertainty, changing needs of customers, pressure to retain customers, demands from users or stakeholders, not getting expected results from technology, inadequate technology standards, heavy workload, spam or irrelevant emails, lack

Author(s), Year of Publication, Study Location(s)	Publication Type, Methodological Approach, Study Design(s)	Sample Size and Population	Study Aim(s)	Main measurements	Important Results
			technostress and technolust		of support, using obsolete systems, many usernames, passwords and profiles to master, inadequate technological infrastructure, techno-accessibility, perceived unreliability and social conflicts<br>• suggestions for improvement included providing adequate resources to support technology projects, sharing idea and best practices in communities, keeping record of passwords, keeping pace with the market, staff planning when implementing new technologies, continuous training of staff, effective communication and change management plans, realistic time scheduling to avoid multitasking, improving technological infrastructure, time to learn and implement new systems, responding only to useful and necessary emails and alerts, developing and maintaining comprehensive technology standards, and prompt decisions
Li and Wang (2020), China	Published article, quantitative, cross-sectional survey	312 university teachers	• to contribute to current debates over the effects of working experience and	Independent Variables:<br>• techno-overload<br>• techno-complexity<br>• techno-insecurity<br>• techno-uncertainty	• literacy facilitation was positively related to technostress creators (techno-overload, techno-complexity, techno-insecurity, techno-uncertainty) (β = .34; $p < .001$; β =

Author(s), Year of Publication, Study Location(s)	Publication Type, Methodological Approach, Study Design(s)	Sample Size and Population	Study Aim(s)	Main measurements	Important Results
			gender on the development of technostress<br>• to explore how the two factors take effect on this issue in higher education<br>• to bridge the gap how specific technostress inhibitors affect specific technostress creators and how they create impact on university teachers' work performance in higher education	• involvement facilitation<br>• technical support provision<br>• literacy facilitation<br>Outcomes:<br>• techno-overload<br>• techno-complexity<br>• techno-insecurity<br>• techno-uncertainty<br>• work performance	.17; $p < .05$; β = .24; $p < .001$; β = .44; $p < .001$)<br>• technical support provision was negatively related to techno-overload (β = -.21; $p < .01$), but not significantly related to techno-complexity, techno-insecurity or techno-uncertainty (β = -.03; n.s.; β = .05; n.s.; β = -.06; n.s.)<br>• involvement facilitation was negatively related to techno-overload (β = -.21; $p < .01$), techno-complexity (β = -.25; $p < .001$), techno-insecurity (β = -.27; $p < .001$), but not techno-uncertainty (β = -.07; n.s.)<br>• techno-overload had a positive negative effect on work performance (β = .16; $p < .05$)<br>• techno-complexity and techno-insecurity were negatively associated with university teachers' work performance (β = -.33; $p < .001$ and β = -.20; $p < .01$)<br>• techno-uncertainty did not have a significant effect on university teachers' work performance (β = .08; n.s.)<br>• literacy facilitation and involvement facilitation were positively related to university teachers' work performance (β = .15; $p < .05$ and β = .18; $p < .01$), while

Author(s), Year of Publication, Study Location(s)	Publication Type, Methodological Approach, Study Design(s)	Sample Size and Population	Study Aim(s)	Main measurements	Important Results
					technical support provision was not (β = .09; n.s.)
Liu, Cheng and Chen (2019), Taiwan	Published article, quantitative, cross-sectional survey	158 physicians	• to explore the influencing factors toward physicians' technostress within mobile electronic medical records settings	Independent Variables:<br>• perceived reliability<br>• perceived usefulness<br>• mobile self-efficacy<br>• technology dependence<br>• perceived complexity<br>Outcomes:<br>• techno-overload<br>• techno-invasion<br>• techno-insecurity<br>• techno-uncertainty	• physicians' perceptions of technology dependence (β = .298; p < .001), mobile self-efficacy (β = -.111; p < .05), and perceived complexity (β = .646; p < .001) were significantly associated with their technostress using mobile electronic medical records, whereas perceived usefulness (β = .083; n.s.) and perceived reliability (β = -.027; n.s.) were not significantly associated with technostress (i.e. techno-overload, techno-invasion, techno-insecurity, techno-uncertainty)
Lorrain (2020), USA	Dissertation, qualitative case study (semi-structured interviews and focus groups)	24 knowledge workers	• to explore how knowledge workers' perceive stress from the use of email<br>• to explore how the appraisal of email affected knowledge worker's perception of eustress and distress within a support	• emails as an easy form of communication<br>• emails reducing phone calls<br>• emails as documenting communication<br>• informal rules about media channels / communication decisions<br>• trust in people and processes<br>• confusion of responsibilities<br>• necessity of emails	• email as an easy form of communication with customers (quick, at convenient times)<br>• business critical: email as a vital form of communication (e.g. reducing phone calls)<br>• email as a method for documenting communication that builds trust that the information has been relayed as described<br>• communication decisions: trust that team members know when not to email was perceived as minimising pressure to check email constantly → phone calls for

Author(s), Year of Publication, Study Location(s)	Publication Type, Methodological Approach, Study Design(s)	Sample Size and Population	Study Aim(s)	Main measurements	Important Results
			organisation that is a division of a large, publicly traded geospatial organization in the United States	• lack of control over dealing with emails<br>• missing out important information<br>• email overload: volume and threads<br>• spam / irrelevant emails<br>• eustress<br>• distress<br>• anxiety	urgent matters, meetings for high amount of required responses<br>• email could be difficult when there is no control over how to manage work along with incoming emails<br>• irrelevant email copies are stressful due to obligation to read them<br>• overload to due difficulty to prioritise between emails, calls, and cases<br>• email overload and difficulty to identify relevant emails<br>• fear of missing out relevant emails leads to checking emails on vacation (techno-invasion) and anxiety over not checking emails often enough<br>• chaos and confusion due to unorganised emails: long email threads make it difficult to follow, ownership/responsibility unclear or whether task is already done/completed
Luoma, Penttinen, and Rinta-Kahila (2020), Finland	Conference paper, qualitative case study, longitudinal semi-structured interviews	10 bank employees	• to improve understanding on human perceptions related to technological change in pre- and	• transparency<br>• workload<br>• customer satisfaction<br>• managerial intervention<br>• empowerment<br>• information overload<br>• techno-invasion	• transparency reduced stress despite surveillance → positively contributed to fairness<br>• reduced workload reduced stress → easier to contact customers (e.g. saving numbers)<br>• customer satisfaction reduced stress → improved ambience of and positive

Author(s), Year of Publication, Study Location(s)	Publication Type, Methodological Approach, Study Design(s)	Sample Size and Population	Study Aim(s)	Main measurements	Important Results
			post-change settings		feedback from customer interactions due to improved availability<br>• managerial intervention reduced stress → organisation of technical training sessions helped to encounter technostress stemming from the inability to master new software<br>• empowerment reduced stress → negotiators could make promises to customers with stronger confidence instead of feeling constrained by old processes and technologies
Ma, Ollier-Malaterre, & Lu (2021), China	Published article, quantitative, cross-sectional (study 1) and longitudinal survey (study 2)	Study 1: 316 IT workers<br>Study 2: 646 female employees using ICT	• to fill a research gap by focussing on the impact of techno-stressors on employees' work-life balance	Study 1 and Study 2:<br>Independent Variables:<br>• techno-stressors (techno-overload, techno-invasion, techno-complexity, techno-insecurity, techno-uncertainty)<br>Moderator:<br>• job self-efficacy<br>Mediator:<br>• emotional exhaustion<br>Outcome:<br>• work-life balance	Study 1:<br>• techno-stressors were significantly negatively associated with work-life balance (β = -.116; $p < .05$)<br>• job self-efficacy significantly moderated (buffered) the relationship between techno-stressors and work-life balance (β = .139; $p < .01$), i.e. the impact of techno-stressors on work-life balance was weaker for employees with higher job self-efficacy<br>• emotional exhaustion significantly mediated the moderation effect for employees with lower levels of job self-efficacy (estimated effect = -.150; 95% CI: -.268 - -.036), but not for employees with

Author(s), Year of Publication, Study Location(s)	Publication Type, Methodological Approach, Study Design(s)	Sample Size and Population	Study Aim(s)	Main measurements	Important Results
					higher levels of job self-efficacy (estimated effect = -.028; 95% CI: -.136 - .061)<br>Study 2:<br>• techno-stressors were significantly negatively associated with work-life balance (β = -.179; $p < .001$)<br>• job self-efficacy significantly moderated (buffered) the relationship between techno-stressors and work-life balance (β = .121; $p < .01$), i.e. the impact of techno-stressors on work-life balance is weaker for employees with higher job self-efficacy<br>• emotional exhaustion significantly mediated the moderation effect for employees with lower levels of job self-efficacy (estimated effect = -.062; LLCI: -.105 - -.020), but not for employees with higher levels of job self-efficacy (estimated effect = -.003; LLCI: -.043 - .037)
Maier, Laumer, Wirth, and Weitzel (2019), Germany and USA	Published article, quantitative, longitudinal survey	Sample 1: 126 German employees and sample 2: 408 US employees (two samples)	• to identify which traits are frequently used in related and recent Information Systems research	Independent Variables:<br>• user involvement<br>• personal innovativeness in IT<br>• neuroticism<br>• IT mindfulness	• the higher the individual's neuroticism, the higher the techno-stressors (sample 1: β = .125; $p < .05$, sample 2: β = .116; $p < .05$)<br>• the higher the personal innovativeness in IT, the lower the techno-stressors

Author(s), Year of Publication, Study Location(s)	Publication Type, Methodological Approach, Study Design(s)	Sample Size and Population	Study Aim(s)	Main measurements	Important Results
			and related to stress<br>• to provide a theoretical explanation for how user personality influences techno-stressors<br>• to explain why some users perceive a certain stimulus as a techno-stressor but others do not<br>• to explain how user personality influences techno-stressors<br>• to study the relative influence of traits from different hierarchical levels on techno-stressors	• age<br>• techno-overload<br>• techno-invasion<br>• techno-complexity<br>• techno-insecurity<br>• techno-uncertainty<br>Mediators:<br>• techno-overload<br>• techno-invasion<br>• techno-complexity<br>• techno-insecurity<br>• techno-uncertainty<br>Outcomes:<br>• neuroticism<br>• personal innovativeness in IT<br>• IT mindfulness<br>• techno-overload<br>• techno-invasion<br>• techno-complexity<br>• techno-insecurity<br>• techno-uncertainty<br>• end user performance<br>• techno-strain<br>• burnout	(sample 1: β = -.159; $p < .05$, sample 2: β = -.142; $p < .05$)<br>• the higher the IT mindfulness, the lower the techno-stressors (sample 1: β = -.388; $p < .001$, sample 2: β = -.239; $p < .01$)<br>• there was no different influence of neuroticism or personal innovativeness in IT on techno-stressors in both samples ($p > .05$)<br>• IT mindfulness had a stronger effect on techno-stressors in both samples ($p < .05$)<br>• the higher the techno-stressors, the higher the likelihood of job burnout (sample 1: β = .360; $p < .01$, sample 2: β = .511; $p < .001$)<br>• techno-stressors had a negative quadratic effect on user performance (effect size of quadratic effect: .11 for sample 1 and .05 for sample 2)<br>• the higher the user involvement, the lower the techno-stressors (sample 1: β = -.063; n.s., sample 2: β = .033; n.s.)<br>• the higher the user involvement, the lower the likelihood of job burnout (sample 1: β = -.179; $p < .05$, sample 2: β = -.054; n.s.)<br>• the higher the user involvement, the higher the user performance (sample 1: β

Author(s), Year of Publication, Study Location(s)	Publication Type, Methodological Approach, Study Design(s)	Sample Size and Population	Study Aim(s)	Main measurements	Important Results
					= .533; $p < .001$, sample 2: $\beta = .452$; $p < .001$)
Mellner (2016), Sweden	Published article, quantitative, cross-sectional survey	2,876 employees	• to investigate the inter-relationships between after-hours availability expectations, work-related smartphone use during leisure, boundary control, and psychological detachment	Independent Variables: • boundary control • frequent work-related smartphone use during leisure • after-hours availability expectations Moderators: • boundary control Outcomes: • psychological detachment	• both after-hours availability expectations and work-related smartphone use during leisure, respectively, were negatively related to psychological detachment ($\beta = -.16$; $p < .001$; $\beta = -.19$; $p < .001$) • boundary control was positively related to psychological detachment ($\beta = .25$; $p < .001$) • boundary control moderated between both after-hours availability expectations ($\beta = .04$; $p < .05$) and psychological detachment • boundary control moderated between work-related smartphone use during leisure and psychological detachment ($\beta = .05$; $p < .05$)
Okolo, Kamarudin, and Ahmad (2018), Nigeria[5]	(Edited) Book chapter, quantitative, cross-sectional survey	319 front desk commercial bank employees	• to examine the how job design (job autonomy, skill variety, task identity, task significance and task feedback) jointly influence banking employees'	Independent Variables: • job design • techno-overload • techno-invasion • techno-complexity • techno-insecurity • techno-uncertainty Mediators: • techno-overload • techno-invasion	• job design was negatively associated with technostress (i.e. techno-overload, techno-invasion, techno-complexity, techno-insecurity, techno-uncertainty) ($t = 11.137$; $p < .001$) • technostress (i.e. techno-overload, techno-invasion, techno-complexity, techno-insecurity, techno-uncertainty) was negatively related to employee engagement ($t = 4.700$; $p < .001$)

Author(s), Year of Publication, Study Location(s)	Publication Type, Methodological Approach, Study Design(s)	Sample Size and Population	Study Aim(s)	Main measurements	Important Results
			technostress and engagement and the influence of job design on employee engagement	• techno-complexity<br>• techno-insecurity<br>• techno-uncertainty<br>Outcomes:<br>• techno-overload<br>• techno-invasion<br>• techno-complexity<br>• techno-insecurity<br>• techno-uncertainty<br>• employee engagement	• job design was positively related to employee engagement ($t = 13.95$; $p < .001$)<br>• the relationship between job design and employee engagement was mediated by technostress (i.e. techno-overload, techno-invasion, techno-complexity, techno-insecurity, techno-uncertainty) ($t = 4.196$; $p < .001$)
Penn (2016), USA	Dissertation, qualitative, semi-structured interviews	6 logistic managers	• to explore the mitigation strategies that logistics managers used to reduce technostress with their employees	• employee well-being and health programmes<br>• understanding employee differences<br>• communication and training for technology proficiency<br>• knowledge sharing<br>• hiring and training temporary IT employees / experts<br>• relying upon specialised IT experts within organisation<br>• leadership<br>• time management and prioritising<br>• adaptive coping strategies	• relying upon specialised IT experts within the organisation: approximately 83% of the sample discussed the support and assistance that their internal IT teams provide employees to help mitigate technostress in the workplace<br>• hiring and training seasonal and temporary IT employees: approximately 83% of the sample shared that hiring temporary, seasonal, part-time, and intern staff to assist regular permanent employees helped to reduce technostress in the workplace; publically available artifacts including documents, such as company newsletters, indicated that added staffers were mitigation strategies for reducing technology-related problems

Author(s), Year of Publication, Study Location(s)	Publication Type, Methodological Approach, Study Design(s)	Sample Size and Population	Study Aim(s)	Main measurements	Important Results
				• techno-overload<br>• techno-invasion<br>• techno-complexity<br>• techno-insecurity<br>• techno-uncertainty<br>• interruptions<br>• work experience<br>• age<br>• knowledge management<br>• work performance<br>• employee absenteeism<br>• job satisfaction<br>• mental health<br>• physical health	• maintaining communications and training for technology proficiency and productivity: all of the participants in the study identified effective training as a key strategy used to reduce technostress in the workplace<br>• communication through regular and special meetings revolved around mitigation strategies to improve productivity through enhancing technology proficiencies<br>• establishing and evaluating progress toward time management and productivity goals; time management was the key strategy that all of the participants in the study claimed could lead to reduction in technostress; publically available artifacts including documents, such as company newsletters and published company updates reflected that mitigation strategies include planning that revolved around productivity and organisation goals<br>• understanding individual differences related to technology proficiency and comfort: all of the participants emphasised the role of comfort and proficiency in the development of

Author(s), Year of Publication, Study Location(s)	Publication Type, Methodological Approach, Study Design(s)	Sample Size and Population	Study Aim(s)	Main measurements	Important Results
					technostress (the differences that participants referenced pertained largely to perceptions about generational differences)<br>• implementing health, fitness, and stress reduction programs for employee: the majority of the participants discussed the need for employees to have healthy ways to reduce the types of technostress they experienced from their work e.g. being able to leave work at work was a way cited by participants in the study for technostress reduction; documentation to support these findings include work schedules, vacation, personal days, and benefit references, but there were no other public artifacts including documents reviewed that included direct references to actual stress reduction programs in the workplace
Pfaffinger, Reif, & Spieß (2020), Germany	Published journal article, quantitative, cross-sectional survey	Study 1 (not included, not relevant for research question of scoping review)<br>Study 2: 142 employees	• to replicate previous findings<br>• to extend existing research on specific aspects of well-being by conceptualising	Study 2:<br>Independent Variables:<br>• techno-overload<br>• techno-invasion<br>• techno-complexity<br>• techno-insecurity<br>• techno-uncertainty	Study 2:<br>• technostress creators (techno-overload, techno-invasion, techno-complexity, techno-insecurity, techno-uncertainty, techno-induced role ambiguity) were significantly positively related to stress and strain n (β = .79; p < .001) and negatively related to engagement and

Author(s), Year of Publication, Study Location(s)	Publication Type, Methodological Approach, Study Design(s)	Sample Size and Population	Study Aim(s)	Main measurements	Important Results
		Study 3: 316 employees	well-being in a more holistic way	• techno-induced role ambiguity<br>Moderators:<br>• literacy facilitation<br>• involvement facilitation<br>• technical support provision<br>Outcomes:<br>• stress and strain<br>• engagement and satisfaction<br>• organisational commitment<br>Study 3:<br>Independent Variables:<br>• techno-overload<br>• techno-invasion<br>• techno-complexity<br>• techno-insecurity<br>• techno-uncertainty<br>• techno-induced role ambiguity<br>Moderators:<br>• literacy facilitation<br>• involvement facilitation<br>• technical support provision<br>Outcomes:	satisfaction ($\beta = -.29$; $p = .002$) as well as organisational commitment ($\beta = -.51$; $p < .001$)<br>• technostress inhibitors (literacy facilitation, involvement facilitation, technical support provision) were significantly negatively related to engagement and satisfaction ($\beta = .20$; $p = .009$) and organisational commitment ($\beta = .54$; $p < .001$), while the relationship with stress and strain was not ($\beta = -.19$; $p = .058$)<br>• technostress inhibitors significantly moderated (buffered) the relationship between technostress creators and stress and strain ($\beta = -.42$; $p = .039$)<br>• technostress inhibitors significantly moderated (amplified) the relationship between technostress creators and engagement and satisfaction ($\beta = .33$; $p = .034$) as well as between technostress creators and organisational commitment ($\beta = .61$; $p = .014$)<br>Study 3:<br>• technostress creators were significantly negatively related to detachment ($\beta = -.37$; $p < .001$) and technostress inhibitors

Author(s), Year of Publication, Study Location(s)	Publication Type, Methodological Approach, Study Design(s)	Sample Size and Population	Study Aim(s)	Main measurements	Important Results
				• detachment	were significantly positively related to detachment (β = .15; *p* = .036)<br>• technostress inhibitors significantly moderated (amplified) the relationship between technostress creators and detachment (β = .24; *p* = .018)
Pflügner, Maier, & Weitzel (2021), Germany	Published journal article, quantitative, cross-sectional survey	134 white-collar workers	• to understand how mindfulness reduces the perception of techno-stressors and whether it mitigates the effect of perceived techno-stressors on job burnout	Independent Variables:<br>• techno-overload<br>• techno-invasion<br>• techno-complexity<br>• techno-insecurity<br>• techno-uncertainty<br>• mindfulness<br>Moderator:<br>• mindfulness<br>Outcome:<br>• techno-overload<br>• techno-invasion<br>• techno-complexity<br>• techno-insecurity<br>• techno-uncertainty<br>• job burnout	• techno-stressors (techno-overload, techno-invasion, techno-complexity, techno-insecurity, techno-uncertainty) were significantly positively associated with job burnout (β = .326; *p* < .001)<br>• mindfulness was significantly negatively associated with techno-stressors (β = -.431; *p* < .001)<br>• mindfulness did not significantly moderate (buffer) the relationship between techno-stressors and job burnout (β = .002; n.s.)<br>• mindfulness was significantly negatively associated with job burnout (β = -.384; *p* < .001)<br>• techno-stressors significantly mediated the relationship between mindfulness and job burnout (*B* = -.144; 95% CI: -.268 - -.054)
Pflügner, Reis, Maier, and	Conference paper, qualitative,	38 white-collar workers	• to work on the research gap of not knowing what	• communication measures<br>• techno-overload<br>• techno-invasion	Communication measures for techno-invasion:

Author(s), Year of Publication, Study Location(s)	Publication Type, Methodological Approach, Study Design(s)	Sample Size and Population	Study Aim(s)	Main measurements	Important Results
Weitzel (2020), Germany	semi-structured interviews		comes along with communication measures that reduce techno-invasion and techno-overload and thus show positive and adverse effects of their introduction for employees		1. communication regulation for after-work usage of business end devices usage<br>2. regulation of after-work email traffic<br>• positive effects: clear end of workday, clear separation of work and private life reducing blurring boundaries<br>• negative effects: cut of flexibility, slow-down of work process<br>Communication measures for techno-overload:<br>1. improved communication with executives<br>2. best practices for internal communication<br>3. efficient communication to get support<br>• positive effects: overall amount of demands the employee received is reduced, information infrastructure and organisation is strengthened<br>• negative effects: high dependence on the employees' trust towards the executive, high dependence on the technical capacities of the company

Author(s), Year of Publication, Study Location(s)	Publication Type, Methodological Approach, Study Design(s)	Sample Size and Population	Study Aim(s)	Main measurements	Important Results
Pirkkalainen, Salo, Makkonen, and Tarafdar (2017), Finland	Conference paper, quantitative, cross-sectional survey	1,091 workers	• to take the initial steps to validating such emotional coping responses as means to mitigate technostress<br>• to shed light on the potentially mixed outcomes of emotional coping responses	Independent Variables:<br>• techno-overload<br>• techno-invasion<br>• techno-complexity<br>• techno-insecurity<br>Moderators:<br>• distancing from IT<br>• distress venting<br>Mediators:<br>• IT control<br>Outcomes:<br>• strain	• higher levels of stressors (i.e. techno-overload, techno-invasion, techno-complexity, techno-insecurity) were associated with higher levels of strain ($\beta = .957$; $p < .001$)<br>• IT control was negatively related to strain ($\beta = -.163$; $p < .001$)<br>• distress venting was positively directly related to strain ($\beta = .145$; $p < .05$)<br>• distress venting negatively moderated the relationship between stressors and strain such that the higher the level of distress venting, the weaker the relationship between stressors and strain ($\beta = -.118$; $p < .05$)<br>• distancing from IT positively did not moderate the relationship between stressors and strain such that the higher the level of distancing from IT, the stronger the relationship between stressors and strain ($\beta = .013$; n.s.)<br>• IT control helped in minimizing the effect of distress venting such that the higher the level of IT control, the weaker the moderating effect of distress venting on the relationship between stressors and strain ($\beta = .188$; $p < .05$)

Author(s), Year of Publication, Study Location(s)	Publication Type, Methodological Approach, Study Design(s)	Sample Size and Population	Study Aim(s)	Main measurements	Important Results
					• IT control did not help in minimizing the effect of distancing from IT such that the higher the level of IT control, the weaker the moderating effect of distancing from IT on the relationship between stressors and strain (β = -.139; n.s.)
Pirkkalainen, Salo, Tarafdar, and Makkonen (2019), USA	Published article, quantitative, cross-sectional survey	846 employed full-time IT users	• to examine how proactive and reactive coping behaviours, individually and together, enable organisational IT users to cope with technostress	Independent Variables:<br>• IT control<br>• positive reinterpretation<br>• techno-overload<br>• techno-invasion<br>• techno-complexity<br>• techno-insecurity<br>• techno-uncertainty<br>Moderators:<br>• IT control<br>• positive reinterpretation<br>• distancing from IT<br>• distress venting<br>Outcomes:<br>• IT-enabled productivity	• technostress creators had a negative relationship with IT-enabled productivity such that the higher are the levels of technostress creators (i.e. Techno-overload, Techno-invasion, Techno-complexity, Techno-insecurity, Techno-uncertainty), the lower are the level of IT-enabled productivity (β = -.105; $p < .01$)<br>• distress venting did not moderate the relationship between the technostress creators and IT-enabled productivity such that the higher is the level of distress venting, the less negative is the relationship between the technostress creators and IT-enabled productivity (β = .011; n.s.)<br>• distancing from IT moderated the relationship between the technostress creators and IT-enabled productivity such that the higher is the level of distancing from IT, the less negative is

Author(s), Year of Publication, Study Location(s)	Publication Type, Methodological Approach, Study Design(s)	Sample Size and Population	Study Aim(s)	Main measurements	Important Results
					the relationship between the technostress creators and IT-enabled productivity (β = .082; $p < .01$)<br>• positive reinterpretation had a positive relationship with IT-enabled productivity such that the higher is the level of positive reinterpretation, the higher is the level of IT-enabled productivity (β = .211; $p < .001$)<br>• IT control had a positive relationship with IT-enabled productivity such that the higher is the level of IT control, the higher is the level of IT-enabled productivity (β = .425; $p < .001$)<br>• positive reinterpretation moderated the effect of distress venting on the relationship between the technostress creators and IT-enabled productivity such that the higher is the level of positive reinterpretation, the weaker is the effect of distress venting on the relationship between the technostress creators and IT-enabled productivity (β = -.182; $p < .01$)<br>• positive reinterpretation moderated the effect of distancing from IT on the relationship between the technostress creators and IT-enabled productivity

Author(s), Year of Publication, Study Location(s)	Publication Type, Methodological Approach, Study Design(s)	Sample Size and Population	Study Aim(s)	Main measurements	Important Results
					such that the higher is the level of positive reinterpretation, the stronger is the effect of distancing from IT on the relationship between the technostress creators and IT-enabled productivity (β = .166; *p* < .01)<br>• IT control moderated the effect of distress venting on the relationship between the technostress creators and IT-enabled productivity such that the higher is the level of IT control, the stronger is the effect of distress venting on the relationship between the technostress creators and IT-enabled productivity (β = .155; *p* < .01)<br>• IT control moderated the effect of distancing from IT on the relationship between the technostress creators and IT-enabled productivity such that the higher is the level of IT control, the weaker is the effect of distancing from IT on the relationship between the technostress creators and IT-enabled productivity (β = -.289; *p* < .001)
Pullins, Tarafdar, and Pham (2020), USA	Published article, quantitative, cross-sectional survey	237 sales professionals	• to examine the effect that conditions creating	Independent Variables:<br>• techno-overload<br>• techno-invasion<br>• techno-complexity	• the positive relationship between technostress creators (i.e. Techno-overload, Techno-invasion, Techno-complexity, Techno-insecurity) and role

Author(s), Year of Publication, Study Location(s)	Publication Type, Methodological Approach, Study Design(s)	Sample Size and Population	Study Aim(s)	Main measurements	Important Results
			technostress due to implementation of sales technologies have on sales professionals' role stress and job satisfaction and the role of mitigating factors on these relationships<br>• to measure the change in the levels of role stress, job satisfaction and job commitment after implementation of technologies	• techno-insecurity<br>Moderators:<br>• job commitment<br>• technical support provision<br>• involvement facilitation<br>• literacy facilitation<br>Outcomes:<br>• role stress change<br>• job satisfaction change	stress change was supported (β = .396; $p < .01$)<br>• the negative relationship between technostress creators and job satisfaction change was partially supported (β = -.133; $p < .10$)<br>• the interaction between technostress inhibitors (i.e. technical support provision, involvement facilitation, literacy facilitation) and technostress creators had a positive effect on job satisfaction change, indicating that the higher the level of technostress inhibitors, the stronger the negative effect of technostress creators on the increase in job satisfaction (β = .273; $p < .05$)<br>• the interaction between technostress inhibitors and technostress creators had a negative effect on role stress change (β = -.127; $p < .10$)<br>• the moderating effect of job commitment on the relationship between technostress creators and job satisfaction change was found insignificant (Δ(1) = 3.503; $p > .05$)<br>• sales professionals with high job commitment (β = .413) had a higher positive effect of technostress creators on

Author(s), Year of Publication, Study Location(s)	Publication Type, Methodological Approach, Study Design(s)	Sample Size and Population	Study Aim(s)	Main measurements	Important Results
					the increase of role stress than the low job commitment (β = .219) (Δ(1) = 3.916; $p < .05$)<br>• the negative effect of technostress creators on the increase of job satisfaction was found not significant for people with high job commitment, but the negative effect of technostress creators on the increase in job satisfaction (β = -.248; $p < .05$) was significant for people with low job commitment
Ragu-Nathan, Tarafdar, and Ragu-Nathan (2008), USA	Published article, quantitative, cross-sectional survey	608 white-collar employees	• to develop and empirically validate two second order constructs: technostress<br>• creators and technostress inhibitors<br>• to test the conceptual model using data from the responses of end users of ICTs from multiple organizations to a	Independent Variables:<br>• techno-overload<br>• techno-invasion<br>• techno-complexity<br>• techno-insecurity<br>• techno-uncertainty<br>• organisational commitment<br>• literacy facilitation<br>• involvement facilitation<br>• technical support provision<br>Outcomes:<br>• continuance commitment<br>• organisational commitment<br>• job satisfaction	• technostress creators (i.e. techno-overload, techno-invasion, techno-complexity, techno-insecurity, techno-uncertainty) were negatively related to job satisfaction (β = -.13; $p < .01$)<br>• technostress inhibitors (i.e. literacy facilitation, involvement facilitation, technical support provision) were positively related to job satisfaction (β = .34; $p < .01$)<br>• technostress inhibitors were positively related to organisational commitment (β = .39; $p < .01$)<br>• technostress inhibitors were positively related to continuance commitment (β = .13; $p < .01$)

Author(s), Year of Publication, Study Location(s)	Publication Type, Methodological Approach, Study Design(s)	Sample Size and Population	Study Aim(s)	Main measurements	Important Results
			survey questionnaire		• technostress inhibitors did not moderate (i.e. weaken) the relationship between technostress creators and job satisfaction (n.s.)
Rayburn, Badrinarayanan, Anderson, & Gupta, (2021), USA and Canada	Published article, quantitative, cross-sectional survey	134 business-to-business salespeople	• To fill the gap considering techno-training and salespeople's beliefs about technology, sales-efficacy and performance	Independent Variables: • continuous techno-training • techno-expectancy • techno-efficacy • technostress (i.e. techno-complexity) • sales-efficacy Outcomes: • techno-expectancy • techno-efficacy • technostress (i.e. techno-complexity) • sales-efficacy • sales effort • sales performance	• continuous techno-training was significantly positively related to techno-expectancy (β = .59; $p < .001$) and to techno-efficacy (β = .45; $p < .001$ / β = .17; $p < .05$ in the revised model), and significantly negatively related to technostress/techno-complexity (β = -.21; $p < .05$) • techno-efficacy was significantly positively related to sales-efficacy (β = .25; $p < .01$ / β = .33; $p < .001$ in the revised model) • techno-expectancy (β = .05; $p > .05$) and technostress (β = -.14; $p > .05$) were not significantly related to sales-efficacy • sales-efficacy was significantly positively related to sales effort (β = .21; $p < .05$ / β = .20; $p < .05$ in the revised model) and sales performance (β = .23; $p < .001$ / β = .22; $p < .001$ in the revised model) • techno-expectancy (β = .37; $p < .001$) and technostress/techno-complexity (β = -.26; $p < .001$) were significantly positively related to techno-efficacy

Author(s), Year of Publication, Study Location(s)	Publication Type, Methodological Approach, Study Design(s)	Sample Size and Population	Study Aim(s)	Main measurements	Important Results
					• techno-efficacy significantly mediated the relationship between techno-expectancy and sales-efficacy (β = .12; $p < .05$) and between technostress/techno-complexity and sales-efficacy (β = -.08; $p < .05$)
Saxena and Lamest (2018), Ireland	Published article, qualitative case study, semi-structured interviews, documentation and direct observation	5 hotel industry managers	• to fill the gap on how managers and organisations deal with this information overload and to provide empirical evidence from the hotel industry within the hospitality sector	• development of dashboards<br>• looking for summary and trends<br>• filtering / being selective about data and sources<br>• moving towards web-based data<br>• withdrawal<br>• qualitative nature of data<br>• information overload	• relationships between platforms interactive dashboard systems that established a link between different online platforms encompassing the quantitative, qualitative and the web traffic data available (constantly updated metrics, providing benchmarking values and actual elaborations on the cause of performance the numerical value, allowing managers to integrate publicly available attitude data about their own company with competitor data)→ necessary reaction to the information overload experienced by managers that resulted in operational decisions e.g. new room-card systems and room restyling<br>• manual summarising activity: striving for clarity and comparability in the context of unstructured data, counting instances to quantify them and make them comparable and valuable →

Author(s), Year of Publication, Study Location(s)	Publication Type, Methodological Approach, Study Design(s)	Sample Size and Population	Study Aim(s)	Main measurements	Important Results
					managers used a combination of filtering, withdrawal and summarising strategies to identify trends<br>• being selective about endless data → filtering strategy, only focussing on relevant platforms<br>• increasing power of customer data through shift from traditional data (surveys) to web-based data (platforms) → rich and cheap information for tactical marketing activities<br>• withdrawal strategy where there was a gradual move from traditional forms of data (e.g. comment cards or email surveys) to web-based data<br>• large amount of unstructured information available was perceived as overwhelming<br>• the data available to managers often reached a level of comprehensiveness that exceeded their capacity to incorporate these data into their activities and often appeared overwhelming<br>• one major source of information overload was the volume and pace of the digital data as well as the unsolicited nature of information was a major

Author(s), Year of Publication, Study Location(s)	Publication Type, Methodological Approach, Study Design(s)	Sample Size and Population	Study Aim(s)	Main measurements	Important Results
					distress for managers causing information overload<br>• the qualitative nature of information also helped mangers in drilling down to isolate the root cause of the problems<br>• the public visibility of the information was a major contributor to information overload to the managers because they realised that the publicly available information could be used by other customers for their decision-making → loss of control
Shirish (2021), France	Published article, quantitative, cross-sectional survey	165 ICT-based mobile managers	• to explain the importance of cognitive and affective processes used amongst ICT-based mobile workers when coping with techno-stressors	Independent Variables:<br>• techno-overload<br>• techno-invasion<br>• techno-complexity<br>• techno-insecurity<br>• techno-uncertainty<br>• threat technology frame<br>• opportunity technology frame<br>Mediators:<br>• threat technology frame<br>• opportunity technology frame<br>• expected affect towards ICT use<br>Outcome:	• techno-stressors (techno-overload, techno-invasion, techno-complexity, techno-insecurity, techno-uncertainty) were significantly positively associated with techno-strain ($B = .379$; $p = .001$)<br>• threat technology frame significantly mediated the relationship between techno-stressors and techno-strain ($ab = .264$; $p < .001$)<br>• opportunity technology frame significantly mediated the relationship between techno-stressors and techno-strain ($ab = .084$; $p < .01$)<br>• expected affect towards ICT use significantly mediated the relationship

Author(s), Year of Publication, Study Location(s)	Publication Type, Methodological Approach, Study Design(s)	Sample Size and Population	Study Aim(s)	Main measurements	Important Results
				• techno-strain	between threat technology frame and techno-strain (*ab* = .069; $p < .01$)<br>• expected affect towards ICT use significantly mediated the relationship between opportunity technology frame and techno-strain (*ab* = .070; $p < .01$)
Shu, Tu, and Wang (2011), China	Published article, quantitative, cross-sectional survey	289 employees	• to develop a conceptual model in which computer-related technostress is studied as consequences of computer self-efficacy and technology dependence	Independent Variables:<br>• computer self-efficacy<br>• technology dependence<br>Outcomes:<br>• techno-overload<br>• techno-invasion<br>• techno-complexity<br>• techno-insecurity<br>• techno-uncertainty	• there was a negative relationship between computer self-efficacy and technostress (i.e. techno-overload, techno-invasion, techno-complexity, techno-insecurity, techno-uncertainty) ($\beta = -.37$; $p < .01$; $t = -4.69$) → computer self-efficacy had a significant negative relationship with techno-complexity ($\beta = -.152$) and techno-insecurity ($\beta = -.375$), but not with the other three components of technostress (techno-overload, techno-invasion, and techno-uncertainty)<br>• technology dependence had a direct and positive impact on technostress ($\beta = .35$; $p < .01$; $t = 4.29$)<br>• employees with low computer self-efficacy and high technology dependence (type II) perceived the highest level of technostress, whereas those with high computer self-efficacy and low technology dependence (type III) perceived the lowest technostress

Author(s), Year of Publication, Study Location(s)	Publication Type, Methodological Approach, Study Design(s)	Sample Size and Population	Study Aim(s)	Main measurements	Important Results
					(difference of technostress level between II and III p = .001)<br>• employees with high computer self-efficacy and high technology dependence (type IV) perceived more technostress than those with low computer self-efficacy and low technology dependence (type I), but the difference between type IV and I was not statistically significant
Stadin, Nordin, Fransson, and Broström (2020), Sweden	Published article, qualitative, semi-structured interviews/critical incident technique	20 healthcare managers	• to describe healthcare managers' experience of technostress and their actions for handling it	• efficient communication management / good email culture<br>• best practices for internal communication<br>• technical support provision<br>• back-up routines<br>• administrative support<br>• ICT organisational support<br>• social support<br>• relying on co-workers' digital literacy<br>• digital literacy / competence to manage IT systems<br>• routine and structure<br>• flexibility in replying to digital communication	experiences of technostress:<br>• high workload (high number of digital messages, redundant digital messages, demands for rapid replies to digital messages)<br>• invasion of private life (limitless digital communication management, distressing messages after working hours)<br>• negative feeling (fear of missed out information in the inbox, misunderstandings or frustration in digital communication)<br>• time consuming ICT systems (illogical ICT systems, sequence-ordered ICT systems, login frustration, slow response time)<br>• malfunctioning ICT systems (technical struggle, disturbances

Author(s), Year of Publication, Study Location(s)	Publication Type, Methodological Approach, Study Design(s)	Sample Size and Population	Study Aim(s)	Main measurements	Important Results
				• using digital solutions<br>• using separate devices for work and private life<br>• confident attitude<br>• learning by doing<br>• preparation<br>• improvisation<br>• need for redistribution of work and administrative support<br>• terminology misfit<br>• need for user influence / involvement<br>• fear of missing out important information<br>• email overload<br>• perceived pressure to respond promptly to emails<br>• high quantity of emails and redundant emails<br>• techno-invasion<br>• lack of digital literacy and need for ICT training<br>• high workload<br>• lack of back-up routines for system breakdowns	from notifications, updates that require action)<br>• facilitating digital literacy (lack of general digital literacy, lack of literacy in ICT systems used irregularly, need fo practical training)<br>• facilitating user influence (need for increased user influence, need for increased terminology fit between ICT systems and healthcare, need for increased use of new digital solutions)<br>• redistribution of work/ICT systems (need for increased administrative support, need for redistribution of ICT systems, need for strengthened back-up routines for system breakdowns)<br>actions related to technostress:<br>• good email culture (efficient digital communication management, meetings instead of emails, communication about digital communication with co-workers)<br>• co-worker support (situation-based co-worker support, reliance on co-

Author(s), Year of Publication, Study Location(s)	Publication Type, Methodological Approach, Study Design(s)	Sample Size and Population	Study Aim(s)	Main measurements	Important Results
				• time consuming ICT systems<br>• malfunctioning ICT systems<br>• misunderstanding in digital communication	worker's digital literacy, supporting each other during system failure)<br>• individual strategies (routine and structure, flexible in replying to digital communication, using digital solutions, using separate digital devices for work and private life)<br>• individual competence (digital literacy, learning by doing, preparation, improvisation, confident attitude)<br>• support and assistance (good IT support, back-up routines, administrative support)
Suh and Lee (2017), South Korea	Published article, quantitative, cross-sectional survey	258 teleworkers	• to develop and test a theoretical model that predicts a teleworker's job satisfaction<br>• to provide researchers and practitioners with meaningful insights concerning ways to increase the effectiveness of	Independent Variables:<br>• task interdependence<br>• job autonomy<br>• IT presenteeism<br>• techno-overload<br>• techno-invasion<br>• techno-complexity<br>• techno-uncertainty<br>Moderators:<br>• intensity of teleworking<br>Outcomes:<br>• work overload<br>• invasion of privacy<br>• job satisfaction	• IT complexity was not significantly positively associated with teleworkers' perceived work overload<br>• IT presenteeism was not significantly positively associated with teleworkers' perceived work overload<br>• IT presenteeism was positively associated with teleworkers' perceived invasion of privacy (β = .11; $p < .05$)<br>• the pace of IT change was positively associated with teleworkers' perceived work overload (β = .40; $p < .01$)

Author(s), Year of Publication, Study Location(s)	Publication Type, Methodological Approach, Study Design(s)	Sample Size and Population	Study Aim(s)	Main measurements	Important Results
			telework implementation in organisations	• strain<br>• role ambiguity	• the pace of IT change was positively associated with teleworkers' perceived role ambiguity (β = .31; $p < .01$)<br>• job autonomy was not significantly negatively associated with teleworkers' perceived work overload<br>• job autonomy was negatively associated with teleworkers' perceived invasion of privacy (β = -.16; $p < .05$)<br>• task interdependence was positively associated with teleworkers' perceived work overload (β = .15; $p < .05$)<br>• task interdependence was positively associated with teleworkers' perceived invasion of privacy (β = .29; $p < .01$)<br>• task interdependence was not significantly positively associated with teleworkers' perceived role ambiguity<br>• work overload was positively associated with teleworkers' strain (β = .23; $p < .05$)<br>• Invasion of privacy was positively associated with teleworkers' strain (β = .11; $p < .05$)<br>• role ambiguity was positively associated with teleworkers' strain (β = .56; $p < .01$)<br>• strain was negatively associated with teleworkers' job satisfaction (β = -.40; $p < .01$)

Author(s), Year of Publication, Study Location(s)	Publication Type, Methodological Approach, Study Design(s)	Sample Size and Population	Study Aim(s)	Main measurements	Important Results
					• the negative influence of strain on job satisfaction was significantly ($p < .001$) greater in the low intensity of teleworking group ($\beta = -.44$; $t = -14.67$) than in the high intensity of teleworking group ($\beta = -.38$; $t = -7.17$)<br>• the influence of role ambiguity on strain was significantly ($p < .001$) greater in the high intensity of teleworking group ($\beta = .68$; $t = 7.20$) than in the low intensity of teleworking group ($\beta = .43$; $t = 6.14$)<br>• the influence of work overload on strain was significantly ($p < .001$) greater in the low intensity of teleworking group ($\beta = .23$; $t = 7.67$) than in the high intensity of teleworking group ($\beta = .12$; $t = 3.00$)<br>• invasion of privacy significantly ($p < .05$) influenced strain in the low intensity of teleworking group ($\beta = .19$; $t = 2.53$) but not in the high intensity of teleworking group<br>• IT complexity significantly ($p < .05$) increased work overload in the low intensity of teleworking group ($\beta = .13$; $t = 2.17$), whereas it does not have any influence on work overload in the high intensity of teleworking group

Author(s), Year of Publication, Study Location(s)	Publication Type, Methodological Approach, Study Design(s)	Sample Size and Population	Study Aim(s)	Main measurements	Important Results
					• IT presenteeism significantly ($p < .05$) increased invasion of privacy in the low intensity of teleworking group (β = .12; t = 2.40) but not in the high intensity of teleworking group<br>• the positive influence of the pace of IT change on role ambiguity was significantly ($p < .001$) greater in the low intensity of teleworking group (β = .48; t = 7.73) than in the high intensity of teleworking group (β = .30; t = 7.5)<br>• the extent to which job autonomy reduces invasion of privacy was significantly ($p < .001$) greater in the high intensity of teleworking group (β = −.20; t = −2.50) than in the low intensity of teleworking group (β = −.11; t = −2.20)
Tarafdar, Tu, and Ragu-Nathan (2010), USA	Published article, quantitative, cross-sectional survey	233 employees of the public sector	• to study technostress-related strains from the end-user computing perspective<br>• to understand the (adverse) effects of technostress on the extent to which end users perceive	Independent Variables:<br>• innovation support<br>• involvement facilitation<br>• techno-overload<br>• techno-invasion<br>• techno-complexity<br>• techno-insecurity<br>• techno-uncertainty<br>Outcomes:<br>• involvement facilitation<br>• end user satisfaction	• technostress creators (i.e. techno-overload, techno-invasion, techno-complexity, techno-insecurity, echno-uncertainty) negatively influenced end-user satisfaction (β = −.18; $p < .05$)<br>• technostress creators negatively influenced end-user performance (β = −.33; $p < .01$)<br>• involvement facilitation negatively influenced technostress creators (β = −.17; $p < .05$)

Author(s), Year of Publication, Study Location(s)	Publication Type, Methodological Approach, Study Design(s)	Sample Size and Population	Study Aim(s)	Main measurements	Important Results
			the applications they use to be satisfactory and can utilise them to improve their performance at work<br>• to identify mechanisms that can mitigate these effects	• techno-overload<br>• techno-invasion<br>• techno-complexity<br>• techno-insecurity<br>• techno-uncertainty<br>• end user performance	• involvement facilitation positively influenced end-user satisfaction (β = .18; $p < .05$)<br>• innovation support positively influenced end-user satisfaction (β = .24; $p < .01$)<br>• innovation support positively influenced involvement facilitation (β = .56; $p < .01$)
Tarafdar, Pullins, and Ragu-Nathan (2015), United Kingdom	Published article, quantitative, cross-sectional survey	237 sales professionals	• to examine the impact of technostress creators on the sales professional's innovation and performance	Independent Variables:<br>• technology competence<br>• technology-enabled innovation<br>• literacy facilitation<br>• technical support provision<br>• involvement facilitation<br>• techno-overload<br>• techno-invasion<br>• techno-complexity<br>• techno-insecurity<br>Moderators:<br>• technology self-efficacy<br>Mediators:<br>• techno-overload<br>• techno-invasion	• technostress creators (i.e. techno-overload, techno-invasion, techno-complexity, techno-insecurity, techno-uncertainty) were negatively related to sales performance (β = -.147; $p < .05$)<br>• technostress creators were negatively related to technology-enabled innovation (β = -.148; $p < .05$)<br>• technology-enabled innovation was positively related to technology-enabled performance (β = .451; $p < .01$)<br>• technology self-efficacy did not negatively moderate the relationship between technostress creators and sales performance<br>• technostress inhibitors (i.e. literacy facilitation, technical support provision,

Author(s), Year of Publication, Study Location(s)	Publication Type, Methodological Approach, Study Design(s)	Sample Size and Population	Study Aim(s)	Main measurements	Important Results
				• techno-complexity<br>• techno-insecurity<br>Outcomes:<br>• technology-enabled performance<br>• technology-enabled innovation<br>• sales performance<br>• techno-overload<br>• techno-invasion<br>• techno-complexity<br>• techno-insecurity	involvement facilitation) were negatively related to technostress creators (β = -.151; *p* < .05)<br>• technology competence was positively related to technology-enabled performance (β = .262; *p* < .01)<br>• technology competence was positively related to technology-enabled innovation (β = .357; *p* < .01)<br>• technology self-efficacy was positively related to sales performance (β = .209; *p* < .01)<br>• technostress inhibitors were positively related to technology-enabled innovation (β = .347; *p* < .01)
Tarafdar, Pirkkalainen, Salo, and Makkonen (2020), United Kingdom and USA	Published article, mixed methods, interviews and cross-sectional survey	Qualitative part: 30 executives (knowledge workers) in the United Kingdom<br>Quantitative part: 846 US employees	• to explain how organisational IT users can cope with technostress<br>• to provide practical recommendations for how organizations can help their employees do so	Qualitative part:<br>• IT use autonomy<br>• demarcation: separating work and private life<br>• time management: setting aside time for tasks and switch off ICT<br>• developing IT use skills and IT competence<br>• taking things with humour<br>• distress venting<br>• distancing from IT<br>• techno-overload	Qualitative part:<br>• reducing IT-related emotions and distress helped individuals to work off their anger and frustration by venting or expressing negative emotions and by distancing or diverting from the IT use situations and switching to other activities<br>• developing IT capacity by a positive IT outlook i.e. being optimistic about technostress situations or by IT use skills i.e. developing competence in IT use

Author(s), Year of Publication, Study Location(s)	Publication Type, Methodological Approach, Study Design(s)	Sample Size and Population	Study Aim(s)	Main measurements	Important Results
				• techno-invasion<br>• techno-complexity<br>• techno-insecurity<br>• techno-uncertainty<br>• IT-enabled productivity<br><br>Quantitative part:<br>Independent Variables:<br>• techno-overload<br>• techno-invasion<br>• techno-complexity<br>• techno-insecurity<br>• techno-uncertainty<br>• IT use autonomy<br>• demarcation: separating work and private life<br>• time management: setting aside time for tasks and switch off ICT<br>• developing IT use skills and IT competence<br>• taking things with humour<br>• distress venting<br>• distancing from IT<br>Moderators:<br>• IT use autonomy<br>• demarcation: separating work and private life	• developing IT use demarcations by IT use autonomy or having control over IT use, by time related demarcations i.e. setting aside specific times for particular types of IT use, and by separating work and non-work IT use<br>Quantitative part:<br>• distress venting decreased the negative effect of technostress creating conditions on IT-enabled productivity (β = -.3; p < .001)<br>• distress venting reduced IT-enabled productivity (β = -.24; p < .001)<br>• distancing from IT decreased the negative effect of technostress creating conditions on IT-enabled productivity (β = -.22; p < .001)<br>• distancing from IT was not significantly related to IT-enabled productivity (β = -.04; n.s.)<br>• positive IT outlook did not change the negative effect of technostress creating conditions on IT-enabled productivity (β = .13; n.s.)<br>• positive IT outlook increased IT-enabled productivity (β = .47; p < .001)

Author(s), Year of Publication, Study Location(s)	Publication Type, Methodological Approach, Study Design(s)	Sample Size and Population	Study Aim(s)	Main measurements	Important Results
				• time management: setting aside time for tasks and switch off ICT<br>• developing IT use skills and IT competence<br>• taking things with humour<br>• distress venting<br>• distancing from IT<br>Outcomes:<br>• IT-enabled productivity	• IT use skills decreased the negative effect of technostress creating conditions on IT-enabled productivity (β = -.15; $p < .1$)<br>• IT use skills increased IT-enabled productivity (β = .53; $p < .001$)<br>• IT use autonomy decreased the negative effect of technostress creating conditions on IT-enabled productivity (β = -.22; $p < .001$)<br>• IT use autonomy increased IT-enabled productivity (β = .38; $p < .001$)<br>• time related demarcations decreased the negative effect of technostress creating conditions on IT-enabled productivity (β = -.20; $p < .01$)<br>• time related demarcations increased IT-enabled productivity (β = .39; $p < .001$)<br>• work and non-work IT use separation decreased the negative effect of technostress creating conditions on IT-enabled productivity (β = -.29; $p < .001$)<br>• work and non-work IT use separation increased IT-enabled productivity (β = .20; $p < .001$)
Turel and Gaudioso (2018), USA and Italy	Published article, quantitative, cross-sectional survey	Sample/case 1: 175 US state government employees and	• to investigate how competitive and leadership climates can	Independent Variables:<br>• competitive climate<br>• positive leadership climate<br>• job distress	• techno-stressors increased employees' levels of distress on the job in both samples (US sample: β = .49; $p < .001$; Italian sample: β = .45; $p < .001$)

Author(s), Year of Publication, Study Location(s)	Publication Type, Methodological Approach, Study Design(s)	Sample Size and Population	Study Aim(s)	Main measurements	Important Results
		sample/case 2: 178 Italian IT professionals	influence how employees experience technostress	• techno-overload<br>• techno-invasion<br>• techno-complexity<br>• techno-insecurity<br>• techno-uncertainty<br>Moderators:<br>• competitive climate<br>• positive leadership climate<br>Outcomes:<br>• job distress<br>• work exhaustion	• the distress employees experience increased their work exhaustion levels in both samples (US sample: β = .62; *p* < .001; Italian sample: β = .60; *p* < .001)<br>• positive leadership climate reduced employees' levels of distress on the job in the US sample (β = -.38; *p* < .001)<br>• leadership climate moderated (i.e. reduced/buffered) the effect of techno-stressors on employees' levels of distress on the job in the US sample (β = -.19; *p* < .01)<br>• competitive climate increased employees' levels of distress on the job in the Italian sample (β = .32; *p* < .001)<br>• competitive climate moderated (i.e. increased) the effect of techno-stressors on employees' levels of distress on the job in the Italian sample (β = .26; *p* < .001)
Wang, Shu, and Tu (2008), China	Published article, quantitative, cross-sectional survey	951 employees	• to investigate the effects of different organisational environment settings on employee technostress levels<br>• to provide a foundation for	Independent Variables:<br>• power centralisation<br>• organisational culture of innovation<br>Outcomes:<br>• techno-overload<br>• techno-invasion<br>• techno-complexity<br>• techno-insecurity	• the extent of power centralisation in an organisation had a positive relationship with the level of employee technostress (β = .242; *p* < .001)<br>• the organisational culture of innovation increased the level of employee technostress (β = .098; *p* = .015)

Author(s), Year of Publication, Study Location(s)	Publication Type, Methodological Approach, Study Design(s)	Sample Size and Population	Study Aim(s)	Main measurements	Important Results
			organisations to understand and alleviate technostress, thus improving employee performance	• techno-uncertainty	• a low centralisation/low innovation organisation had the lowest level of overall technostress<br>• in organisations with high centralisation/high innovation, the level of overall technostress was the highest<br>• techno-invasion had no significant difference, while the others did have a significant difference under different organisational internal environments
Wu, Wang, Mei, and Liu (2017), China	Conference paper, quantitative, cross-sectional survey	374 managers and general staff in manufacturing and IT industry	• to enhance our understanding of whether and how techno-invasion may influence employees' IT-enabled job anxiety<br>• to identify boundary conditions under which the detrimental effects of techno-invasion on job anxiety can be weakened<br>• to investigate whether computer	Independent Variables:<br>• techno-invasion<br>Moderators:<br>• perceived organisational support<br>• computer self-efficacy<br>Outcomes:<br>• job anxiety	• techno-invasion significantly positively affected job anxiety (β_1 = .675; β_2 = .604; p < .001)<br>• computer self-efficacy negatively moderated relationship between techno-invasion and job anxiety (β = -.193; p < .01)<br>• perceived organisational support negatively moderated relationship between techno-invasion and job anxiety (β = -.146; p < .001)<br>• perceived organisational support moderated the moderating effect of computer self-efficacy on the relationship between techno-invasion and job anxiety: at low level of perceived organisational support, the interaction between computer self-efficacy and techno-

Author(s), Year of Publication, Study Location(s)	Publication Type, Methodological Approach, Study Design(s)	Sample Size and Population	Study Aim(s)	Main measurements	Important Results
			self-efficacy and perceived organizational support moderate the relationship between techno-invasion and job anxiety, and further whether there is interaction between two moderators		invasion was significant (β = -.404; $p < .05$); but at high level of perceived organisational support, the moderating effect of computer self-efficacy was not significant (β = -.218; $p > .1$)
Yener, Arslan, and Kilinç (2020), Turkey	Published article, quantitative, cross-sectional survey	319 employees	• to contribute to the gaps in the literature on harmful effects of using technology at work and indirect effects on work performance by analysing a model with two moderators	Independent Variables:<br>• techno-overload<br>• techno-invasion<br>• techno-complexity<br>• techno-insecurity<br>• techno-uncertainty<br>Moderators:<br>• technology self-efficacy<br>Mediators:<br>• burnout<br>Outcomes:<br>• burnout<br>• task performance<br>• contextual performance	• technostress affected task performance in a negative and significant way (β = -.25; $p < .001$)<br>• technostress affected contextual performance in a negative and significant way (β = −.19; $p < .001$)<br>• burnout did not have a mediating role between technostress and task performance (β = -.022; n.s.)<br>• burnout did not have a mediating role between technostress and contextual performance (β = −.023; n.s.)<br>• technological self-efficacy had a moderating role between technostress and burnout (β =-.26; $p < .001$)

Author(s), Year of Publication, Study Location(s)	Publication Type, Methodological Approach, Study Design(s)	Sample Size and Population	Study Aim(s)	Main measurements	Important Results
Yu, Lin, and Liao (2017), Taiwan	Published article, quantitative, cross-sectional survey	875 employees	• to discover the psychological factors that influence information and communication technology (ICT) adoption behaviour<br>• to confirm whether "information literacy" and "digital skills" have moderator effects in the research model	Independent Variables:<br>• media technostress<br>Moderators:<br>• information literacy<br>• digital skill<br>Outcomes:<br>• ICT adoption behaviour	• a medium level of technostress was negatively related to a user's ICT (β = .139; *p* < .01) adoption behaviour of ICT<br>• the relationship between media technostress and ICT adoption behaviour was not significantly moderated by the level of digital skill i.e. the relationship was weaker under conditions of low digital skill and stronger under conditions of high digital skill (β = -.045; n.s.)<br>• the relationship between media technostress and ICT adoption behaviour was not significantly moderated by the level of information literacy i.e. the relationship was weaker under conditions of low information literacy and stronger under conditions of high information literacy (β = -.031; n.s.)
Zainun, Johari, and Adnan (2019), Malaysia	Published article, quantitative, cross-sectional survey	225 administrative employees in public higher education institutions	• to determine the influence of technostress on commitment to change<br>• to determine the moderating effect of internal communication on	Independent Variables:<br>• techno-overload<br>• techno-invasion<br>• techno-complexity<br>• techno-insecurity<br>• techno-uncertainty<br>Moderators:<br>• internal communication<br>Outcomes:	• techno-overload was not significantly negatively related to commitment to change (β = .057; n.s.)<br>• techno-invasion was negatively related to commitment to change (β = -.119; *p* < .05)<br>• techno-complexity was not significantly negatively related to commitment to change (β = -.034; n.s.)

Author(s), Year of Publication, Study Location(s)	Publication Type, Methodological Approach, Study Design(s)	Sample Size and Population	Study Aim(s)	Main measurements	Important Results
			the relationship between technostress and commitment to change	• commitment to change	• techno-insecurity was negatively related to commitment to change (β = -.180; $p < .05$)<br>• techno-uncertainty was not significantly negatively related to commitment to change (β = .125; n.s.)<br>• the negative relationship between techno-overload and commitment to change was not weaker if internal communication was high (β = .044; $t = .497$; n.s.)<br>• the negative relationship between techno-invasion and commitment to change was not weaker if internal communication was high (β = −.027; $t = .208$; n.s.)<br>• the negative relationship between techno-complexity and commitment to change was not weaker if internal communication was high (β = .055; $t = .415$; n.s.)<br>• the negative relationship between techno-insecurity and commitment to change was not weaker if internal communication was high (β = −.048; $t = .336$; n.s.)<br>• the negative relationship between techno-uncertainty and commitment to

Author(s), Year of Publication, Study Location(s)	Publication Type, Methodological Approach, Study Design(s)	Sample Size and Population	Study Aim(s)	Main measurements	Important Results
					change was weaker if internal communication was high (β = .203; t = 2.638; p < .001)
Zhao, Xia, & Huang (2020), China	Published journal article, quantitative, cross-sectional survey	513 employees using ICT	• to examine associations of technostress creators with challenge and hindrance appraisal outcomes<br>• to analyse mediation effects of problem- and emotion-focused coping strategies between challenge and hindrance appraisal outcomes on ICT-enabled productivity	Independent Variables:<br>• techno-overload<br>• techno-invasion<br>• techno-complexity<br>• techno-insecurity<br>• techno-uncertainty<br>• challenge appraisal outcome<br>• hindrance appraisal outcome<br>• problem-focused coping strategies (seeking instrumental support)<br>• emotion-focused coping strategies (psychological distancing and venting)<br>Mediators:<br>• challenge appraisal outcome<br>• hindrance appraisal outcome<br>• problem-focused coping strategies (seeking instrumental support)	• techno-overload was significantly positively associated with challenge appraisal outcome (β = .21; p < .05), but not significantly associated with hindrance appraisal outcome (β = -.05; p > .05)<br>• techno-invasion was neither significantly related to challenge (β = -.08; p > .05) nor hindrance appraisal outcome (β = .03; p > .05)<br>• techno-uncertainty was significantly positively related to challenge (β = .25; p < .05) and hindrance appraisal outcome (β = .16; p < .05)<br>• techno-complexity was significantly negatively associated with challenge appraisal outcome (β = -.23; p < .05) and significantly positively related to hindrance appraisal outcome (β = .28; p < .05)<br>• techno-insecurity was significantly negatively related to challenge appraisal outcome (β = -.11; p < .05) and significantly positively related to

Author(s), Year of Publication, Study Location(s)	Publication Type, Methodological Approach, Study Design(s)	Sample Size and Population	Study Aim(s)	Main measurements	Important Results
				• emotion-focused coping strategies (psychological distancing and venting)<br>Outcomes:<br>• challenge appraisal outcome<br>• hindrance appraisal outcome<br>• problem-focused coping strategies (seeking instrumental support)<br>• emotion-focused coping strategies (psychological distancing and venting)<br>• ICT-enabled productivity	hindrance appraisal outcome (β = .15; $p < .05$)<br>• problem-focused coping strategies mediated the influence of challenge appraisal outcome on ICT-enabled productivity (β = .03; 95% CI = .0029 - .0546)<br>• emotion-focused coping strategies mediated the influence of hindrance appraisal outcome on ICT-enabled productivity (indirect effect through psychological distancing was not significant (β = .002; 95% CI = -.0042 - .0116), but was significant through venting (β = -.04; 95% CI = -.0687 - -.0065))<br>• challenge appraisal outcome was significantly positively related to ICT-enabled productivity (β = .55; $p < .05$)<br>• hindrance appraisal outcome was significantly negatively related to ICT-enabled productivity (β = -.12; $p < .05$)<br>• challenge appraisal outcome was significantly positively associated with seeking instrumental support (β = .36; $p < .05$), which was significantly positively related to ICT-enabled productivity (β = .09; $p < .05$)

Author(s), Year of Publication, Study Location(s)	Publication Type, Methodological Approach, Study Design(s)	Sample Size and Population	Study Aim(s)	Main measurements	Important Results
					• hindrance appraisal outcome was significantly positively associated with psychological distancing (β = .11; $p < .05$) and venting (β = .42; $p < .05$), but whereas venting had a significant negative effect on ICT-enabled productivity (β = -.10; $p < .05$), psychological distancing did not (β = .03; $p > .05$)

Note. If no information on the study location was provided, the first author's location is given. [1] One dyad consists of one employee and two to three corresponding customers. [2] Authors' location: Switzerland. [3] Authors' location: Germany. [4] Authors' location: South Africa. [5] Authors' location: Malaysia.
