# Supplementary material for: Overcoming the “Dark Side” of Technology—A Scoping Review on Preventing and Coping with Work-Related Technostress
Source: Int J Environ Res Public Health. 2022 Mar 18;19(6):3625. doi: 10.3390/ijerph19063625 (PMC8954507; doi:10.3390/ijerph19063625)
Supplement: Supplementary file 1 [file ijerph-19-03625-s001.zip › Supplementary Tables S1-S5.pdf]

Table S1. Search strategy for the medical database PubMed.

	PubMed Search String
#1	work [MeSH] OR workplace [MeSH] OR job [ALL] OR employment [MeSH] OR vocation [ALL] OR occupation [ALL] OR employee* [ALL] OR "organisational context" [ALL] OR "organizational context" [ALL] OR "working space" [ALL]
AND	
#2	"digital stress" [ALL] OR "technology-induced stress" [ALL] OR "technological stress" [ALL] OR technostress* [ALL] OR techno-stress* [ALL] OR "techno-overload" [ALL] OR "techno-complexity" [ALL] OR "techno-uncertainty" [ALL] OR "techno-insecurity" [ALL] OR "techno-invasion" [ALL]
AND	
#3	"workplace intervention" [ALL] OR "health promotion" [MeSH] OR "Primary Prevention" [MeSH] OR "Secondary Prevention" [MeSH] OR "Adaptation, Psychological" [MeSH] OR coping [ALL] OR "stress reduction" [ALL] OR "stress management" [ALL] OR mitigat* [ALL] OR mediat* [ALL] OR moderat* [ALL] OR training [ALL] OR inhibitor* [ALL] OR alleviat* [ALL] OR prevent* [ALL] OR combat* [ALL] OR "situational factor" [ALL] OR intervention [ALL] OR influence [ALL] OR protection [ALL] OR "positive technology" [ALL] OR "Mindfulness-Based Interventions" [ALL] OR mindfulness [MeSH] OR "technical support" [ALL] OR "literacy facilitation" [ALL] OR involvement [ALL] OR Personality [MeSH] OR usefulness [ALL] OR "social support" [ALL] OR "Self Efficacy" [Mesh] OR "innovation support" [ALL] OR venting [ALL] OR disengagement [ALL] OR control [ALL]

Table S2. Search strategy for the medical database MEDLINE.

	MEDLINE Search String
#1	work.sh. or workplace.sh. or job.af. or employment.sh. or vocation.af. or occupation.af. or employee*.af. or organi?ational context.af. or "working space".af.
AND	
#2	"digital stress".af. or "technology-induced stress".af. or "technological stress".af. or technostress*.af. or techno-stress*.af. or "techno-overload".af. or "techno-complexity".af. or "techno-uncertainty".af. or "techno-insecurity".af. or "techno-invasion".af.
AND	
#3	"workplace intervention".af. or health promotion/ or primary prevention/ or Secondary Prevention/ or Adaptation, Psychological/ or coping.af. or "stress reduction".af. or "stress management".af. or mitigat*.af. or mediat*.af. or moderat*.af. or training.af. or inhibitor*.af. or alleviat*.af. or prevent*.af. or combat*.af. or "situational factor".af. or intervention.af. or influence.af. or protection.af. or "positive technology".af. or "Mindfulness-Based Interventions".af. or exp mindfulness/ or "technical support".af. or "literacy facilitation".af. or involvement.af. or Personality/ or usefulness.af. or "social support".af. or exp Self-Efficacy/ or "innovation support".af. or venting.af. or disengagement.af. or control.af.

Table S3. Search strategy for the psychological database PSYNDEX.

	PSYNDEX Search String
#1	work.af. or workplace.af. or job.af. or employment.af. or vocation.af. or occupation.af. or employee*.af. or organi?ational context.af. or "working space".sh.
AND	
#2	"digital stress".af. or "technology-induced stress".af. or "technological stress".af. or technostress*.af. or techno-stress*.af. or "techno-overload".af. or "techno-complexity".af. or "techno-uncertainty".af. or "techno-insecurity".af. or "techno-invasion".af.
AND	
#3	workplace intervention/ or health promotion/ or prevention/ or exp coping behavior/ or coping.af. or "stress reduction".af. or exp stress management/ or mitigat*.af. or mediat*.af. or moderat*.af. or training.af. or inhibitor*.af. or alleviat*.af. or prevent*.af. or combat*.af. or "situational factor".af. or intervention.af. or influence.af. or protection.af. or "positive technology".af. or exp Mindfulness-Based Interventions/ or exp mindfulness/ or "technical support".af. or "literacy facilitation".af. or involvement.af. or exp Personality Traits/ or usefulness.af. or "social support".af. or exp Self-Efficacy/ or "innovation support".af. or venting.af. or disengagement.af. or control.af.

Table S4. Search strategy for the psychological database PsycInfo.

	PsycInfo Search String
#1	work.af. or workplace.af. or job.af. or employment.af. or vocation.af. or occupation.af. or employee*.af. or organi?ational context.af. or "working space".sh.
AND	
#2	"digital stress".af. or "technology-induced stress".af. or "technological stress".af. or technostress*.af. or techno-stress*.af. or "techno-overload".af. or "techno-complexity".af. or "techno-uncertainty".af. or "techno-insecurity".af. or "techno-invasion".af.
AND	
#3	workplace intervention/ or health promotion/ or prevention/ or exp coping behavior/ or coping.af. or "stress and coping measures".sh. or "stress reduction".af. or "stress management".af. or mitigat*.af. or mediat*.af. or moderat*.af. or training.af. or inhibitor*.af. or alleviat*.af. or prevent*.af. or combat*.af. or "situational factor".af. or intervention.af. or influence.af. or protection.af. or "positive technology".af. or exp Mindfulness-Based Interventions/ or exp mindfulness/ or "technical support".af. or "literacy facilitation".af. or involvement.af. or exp Personality Traits/ or usefulness.af. or "social support".af. or exp Self-Efficacy/ or "innovation support".af. or venting.af. or disengagement.af. or control.af.

Table S5. Search strategy for the interdisciplinary database Web of Science.

	Web of Science Search String
#1	TS=(work OR workplace OR job OR employment OR vocation OR occupation OR employee* OR organi?ational context OR "working space")
AND	
#2	TS=("digital stress" OR "technology-induced stress" OR "technological stress" OR technostress* OR techno-stress* OR "techno-overload" OR "techno-complexity" OR "techno-uncertainty" OR "techno-insecurity" OR "techno-invasion")
AND	
#3	TS=("workplace intervention" OR "health promotion" OR prevention OR "coping behavior" OR coping OR "stress reduction" OR "stress management" OR mitigat* OR mediat* OR moderat* OR training OR inhibitor* OR alleviat* OR prevent* OR combat* OR "situational factor" OR intervention OR influence OR protection OR "positive technology" OR "Mindfulness-Based Interventions" OR mindfulness OR "technical support" OR "literacy facilitation" OR involvement OR personality OR usefulness OR "social support" OR Self-Efficacy OR "innovation support" OR venting OR disengagement OR control)
