# Supplementary material for: Overcoming the “Dark Side” of Technology—A Scoping Review on Preventing and Coping with Work-Related Technostress
Source: Int J Environ Res Public Health. 2022 Mar 18;19(6):3625. doi: 10.3390/ijerph19063625 (PMC8954507; doi:10.3390/ijerph19063625)
Supplement: Supplementary file 1 [file ijerph-19-03625-s001.zip › Supplementary Tables S7-S9.pdf]

Table S7. Critical Appraisal of Qualitative Studies.

	Andreou (2019)	Kwanya et al. (2012)	Lorrain (2020)	Luoma et al. (2020)	Penn (2016)	Pflügner et al. (2020)	Saxena & Lamest (2018)	Stadin et al. (2020)	Tarafdar et al. (2020)[1]
Is there congruity between the stated philosophical perspective and the research methodology?	✓	×	✓	✓	✓	✓	✓	✓	×
Is there congruity between the research methodology and the research question or objectives?	✓	✓	✓	✓	✓	✓	✓	✓	✓
Is there congruity between the research methodology and the methods used to collect data?	✓	✓	✓	✓	✓	✓	✓	✓	✓
Is there congruity between the research methodology and the representation and analysis of data?	✓	✓	✓	✓	✓	✓	✓	✓	✓
Is there congruity between the research methodology and the interpretation of results?	✓	×	✓	✓	✓	○	✓	✓	✓
Is there a statement locating the researcher culturally or theoretically?	✓	✓	✓	✓	✓	✓	×	✓	✓
Is the influence of the researcher on the research, and vice- versa, addressed?	✓	×	✓	×	✓	×	×	×	×
Are participants, and their voices, adequately represented?	✓	×	✓	✓	✓	×	✓	✓	✓
Is the research ethical according to current criteria or, for recent studies, and is there evidence of ethical approval by an appropriate body?	×	×	×	×	×	×	×	✓	×
Do the conclusions drawn in the research report flow from the analysis, or interpretation, of the data?	✓	✓	✓	✓	✓	✓	✓	✓	✓

Note. ✓= fulfilled, × = not fulfilled, ○ = partly fulfilled criteria. [1] Qualitative part of mixed-methods study.

Table S8. Critical Appraisal of Longitudinal Studies.

	Benlian (2019)	Cho et al. (2020)	Hauk et al. (2019)	Ma et al. (2021)[1]	Maier et al. (2019)
Were the two groups similar and recruited from the same population?	✓	✓	✓	✓	✓
Were the exposures measured similarly to assign people to both exposed and unexposed groups?	⃠	⃠	⃠	⃠	⃠
Was the exposure measured in a valid and reliable way?	○	✓	✓	✓	✓
Were confounding factors identified?	✓	✓	✓	✓	✓
Were strategies to deal with confounding factors stated?	✓	✓	×	✓	×
Were the groups/participants free of the outcome at the start of the study (or at the moment of exposure)?	✓	×	✓	✓	✓
Were the outcomes measured in a valid and reliable way?	○	✓	✓	✓	✓
Was the follow up time reported and sufficient to be long enough for outcomes to occur?	✓	✓	×	×	✓
Was follow up complete, and if not, were the reasons to loss to follow up described and explored?	×	✓	×	✓	○
Were strategies to address incomplete follow up utilized?	✓	✓	×	✓	✓
Was appropriate statistical analysis used?	✓	✓	✓	✓	○

Note. ✓= fulfilled, × = not fulfilled, ○ = unclear or partly fulfilled criteria, ⃠= criteria not applicable. [1] Study 2 in the paper.

Table S9. Critical Appraisal of Cross-Sectional Studies.

	Alnakhli (2020)	Bauwens et al. (2021)	Becker et al. (2021)	Brown et al. (2014)	Califf & Brooks (2020)	Choi et al. (2020)	Christ-Brende-mühl & Schaar-schmidt (2020)	D'Arcy et al. (2014)	Day et al. (2012)	Delpe-chitre et al. (2019)
Were the criteria for inclusion in the sample clearly defined?	✓	×	✓	×	✓	×	×	✓	×	✓
Were the study subjects and the setting described in detail?	✓	✓	✓	✓	✓	✓	✓	✓	✓	✓
Was the exposure measured in a valid and reliable way?	✓	✓	✓	✓	✓	○	✓	✓	✓	✓
Were objective, standard criteria used for measurement of the condition?	✓	✓	✓	○	✓	✓	✓	○	✓	✓
Were confounding factors identified?	×	✓	✓	✓	✓	✓	✓	✓	✓	✓
Were strategies to deal with confounding factors stated?	⊘	×	×	✓	×	✓	✓	✓	✓	✓
Were the outcomes measured in a valid and reliable way?	✓	✓	✓	✓	✓	○	✓	✓	✓	✓
Was appropriate statistical analysis used?	✓	✓	✓	✓	✓	✓	✓	✓	✓	✓

	Fieseler et al. (2014)	Fischer & Riedl (2020)	Gaudi-oso et al. (2017)	Gimpel et al. (2019)	Goetz & Böhm (2020)	Harris et al. (2013)	Harris et al. (2015)	Hung et al. (2011)	Hung et al. (2015)	Hwang & Cha (2018)
Were the criteria for inclusion in the sample clearly defined?	×	×	×	✓	✓	✓	✓	✓	✓	✓
Were the study subjects and the setting described in detail?	✓	✓	✓	✓	✓	✓	✓	✓	✓	✓
Was the exposure measured in a valid and reliable way?	✓	✓	✓	×	✓	✓	✓	×	✓	✓
Were objective, standard criteria used for measurement of the condition?	✓	✓	✓	×	✓	✓	✓	○	✓	✓
Were confounding factors identified?	×	✓	✓	×	✓	✓	✓	×	×	✓
Were strategies to deal with confounding factors stated?	⊘	✓	✓	⊘	✓	✓	✓	⊘	⊘	×
Were the outcomes measured in a valid and reliable way?	✓	✓	✓	×	✓	✓	✓	×	✓	✓
Was appropriate statistical analysis used?	✓	✓	✓	○	✓	✓	✓	✓	✓	✓

	Ioannou & Papaza- [illegible]eiropoulou (2017)	Jena (2015)	Kim & Lee (2021)	Koo & Wati (2011)	Li & Wang (2020)	Liu et al. (2019)	Ma et al. (2021)[1]	Mellner (2016)	Okolo et al. (2018)	Pfaffinger et al. (2020)
Were the criteria for inclusion in the sample clearly defined?	✓	×	×	×	✓	○	○	○	×	✓
Were the study subjects and the setting described in detail?	✓	✓	✓	✓	✓	×	✓	✓	×	✓
Was the exposure measured in a valid and reliable way?	✓	✓	✓	✓	✓	✓	✓	✓	✓	✓
Were objective, standard criteria used for measurement of the condition?	✓	✓	✓	×	✓	✓	✓	✓	✓	✓
Were confounding factors identified?	×	×	×	×	○	×	✓	✓	×	✓
Were strategies to deal with confounding factors stated?	⦸	⦸	⦸	⦸	×	⦸	✓	✓	⦸	✓
Were the outcomes measured in a valid and reliable way?	✓	✓	✓	✓	✓	✓	✓	✓	✓	✓
Was appropriate statistical analysis used?	✓	✓	✓	×	✓	✓	✓	✓	✓	✓

	Pflügner et al. (2021)	Pirkka-lainen et al. (2017)	Pirkka-lainen et al. (2019)	Pullins et al. (2020)	Ragu-Nathan et al. (2008)	Rayburn et al. (2021)	Shu et al. (2011)	Shirish (2021)	Suh & Lee (2017)	Tarafdar et al. (2010)	Tarafdar et al. (2015)
Were the criteria for inclusion in the sample clearly defined?	✓	✓	✓	✓	○	○	✓	✓	○	✓	✓
Were the study subjects and the setting described in detail?	✓	✓	✓	✓	✓	✓	✓	✓	✓	✓	✓
Was the exposure measured in a valid and reliable way?	✓	✓	✓	✓	✓	✓	✓	✓	○	✓	✓
Were objective, standard criteria used for measurement of the condition?	✓	✓	✓	✓	○	✓	✓	✓	✓	✓	✓
Were confounding factors identified?	✓	✓	✓	×	✓	✓	✓	✓	×	×	✓
Were strategies to deal with confounding factors stated?	✓	✓	✓	⊘	✓	✓	×	✓	⊘	⊘	×
Were the outcomes measured in a valid and reliable way?	✓	✓	✓	✓	✓	✓	✓	✓	○	✓	✓
Was appropriate statistical analysis used?	✓	✓	✓	✓	✓	✓	✓	✓	✓	✓	✓

	Tarafdar et al. (2020)[2]	Turel & Gaudioso (2018)	Wang et al. (2008)	Wu et al. (2017)	Yener et al. (2020)	Yu et al. (2017)	Zainun et al. (2020)	Zhao et al. (2020)
Were the criteria for inclusion in the sample clearly defined?	×	○	○	○	×	✓	×	✓
Were the study subjects and the setting described in detail?	✓	✓	✓	✓	✓	✓	✓	✓
Was the exposure measured in a valid and reliable way?	✓	✓	○	✓	✓	✓	✓	✓
Were objective, standard criteria used for measurement of the condition?	✓	✓	✓	✓	✓	○	✓	✓
Were confounding factors identified?	×	✓	×	○	✓	×	×	✓
Were strategies to deal with confounding factors stated?	⃠	✓	×	×	×	⃠	⃠	✓
Were the outcomes measured in a valid and reliable way?	✓	✓	✓	✓	✓	✓	✓	✓
Was appropriate statistical analysis used?	✓	✓	✓	✓	✓	✓	✓	✓

Note. ✓= fulfilled, × = not fulfilled, ○ = unclear or partly fulfilled criteria, ⃠= criteria not applicable. [1] Study 1 in the paper. [2] Quantitative part of mixed-methods study.
